# Simultaneous Generation of Methyl Esters and CO in Lignin Transformation

**DOI:** 10.1002/anie.202209093

**Published:** 2022-09-01

**Authors:** Mingyang Liu, Buxing Han, Paul J. Dyson

**Affiliations:** ^1^ Beijing National Laboratory for Molecular Sciences Key Laboratory of Colloid and Interface and Thermodynamics CAS Research/Education Center for Excellence in Molecular Sciences Institute of Chemistry Chinese Academy of Sciences Beijing 100190 P. R. China; ^2^ Institute of Chemical Sciences and Engineering Swiss Federal Institute of Technology (EPFL) 1015 Lausanne Switzerland; ^3^ School of Chemistry and Chemical Engineering University of Chinese Academy of Sciences Beijing 100049 P. R. China

**Keywords:** Biomass, Carbonylation, Methylation, Cascade reactions, Lignin

## Abstract

Lignin is an abundant renewable carbon source. Due to its complex structure, utilization of lignin is very challenging. Herein, we describe an efficient strategy for the simultaneous utilization of lignin, in which the methoxy groups in lignin react with carboxylic acids to generate methyl carboxylates and the other alkyl and phenyl carbons react with oxygen to predominantly form CO that can be used directly in carbonylation reactions. The method was applied to the methylation of various functionalized aryl and alkyl carboxylic acids, including natural compounds, to produce valuable chemicals, including pharmaceuticals. No solid or liquid residues remain after the reaction. Mechanistic studies demonstrate that a well‐ordered C−C and C−O bond activation sequence takes place to realize total transformation of lignin. This work opens a way for transformation of the entire lignin polymer into valuable products, exemplified by the synthesis of the pharmaceutical, Ramipril, on a gram scale.

## Graphical abstract

Complete utilization of lignin: the methoxy groups in lignin react with carboxylic acids to generate methyl carboxylates (applied to the synthesis of pharmaceuticals), and the remaining carbon atoms of lignin react with oxygen to form CO predominantly, which may be used directly in carbonylation reactions.

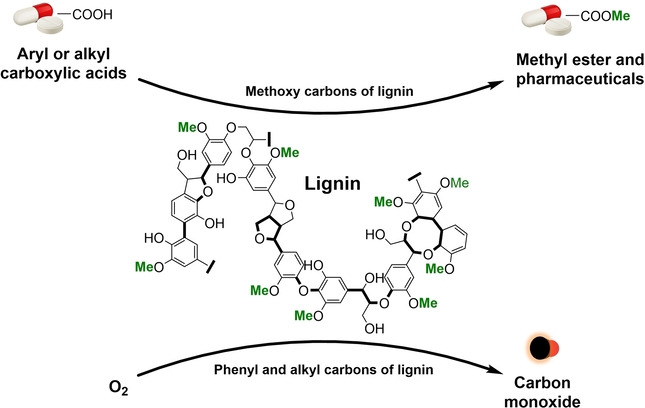

## Introduction

Lignin is an abundant renewable carbon resource that is currently under‐utilized as a source of chemicals.^[1]^ Lignin is used as precursor in the synthesis of solid materials, including carbon fibers^[2]^ and composites,^[3]^ and can be transformed to produce liquid chemicals and fuels, such as aromatics,[^1^, ^4^] carboxylic acids,^[5]^ and their derivatives.^[6]^ Although considerable progress has been made with these transformations, the development of new highly efficient methods that transform lignin into value‐added chemicals is required to enhance the sustainability, diversity, and economic viability of the chemical and pharmaceutical industries.[^4a^, ^7^]

Lignin is predominantly constructed from methoxy and propylbenzene (C9) units, but utilization of the methoxy groups is under‐developed compared with the common transformation of C9 units to value‐added chemicals.[^1^, ^4^] Several strategies that utilize the methoxy groups in lignin have been reported previously. These strategies include metal‐catalyzed hydrodeoxygenation of lignin to generate CH_3_OH at high temperatures (>450 K) and pressures (>10 bar).^[8]^ Noble‐metal‐catalyzed coupling of methoxy groups with CO^[9]^ or CO_2_ ^[10]^ affords small molecules such as acetic acid[^9^, ^10^] or ethanol.^[11]^ The methoxy groups in lignin have also been exploited in the *N*‐methylation of amines using stoichiometric amounts of LiI as a sacrificial reagent.^[12]^ Moreover, in these reported routes only the methoxy group was utilized and the majority of the lignin remained unused.[^9^, ^10^, ^11^, ^12^] Therefore, strategies that totally valorize lignin (the methoxy groups as well as other carbons in lignin) would be advantageous.

Esters have wide applications in the synthesis of both bulk and commodity chemicals. In particular, methyl esters exhibit unique biological properties and are widely present in pharmaceuticals.^[13]^ Well‐established esterification processes tend to involve non‐renewable, toxic reagents.^[14]^ Hence, the production of esters using renewable carbon resources (e.g., lignin) is highly desirable from both environmental and industrial viewpoints as they are benign and inexpensive. However, it would be highly advantageous if the entire lignin structure could be valorized under mild condition.

Based on the classification of lignin as being constructed from methoxy and C9 units,^[1a]^ we designed a Cu‐based catalytic system that promotes methylation of carboxylic acids with the methoxy carbons to generate methyl esters as liquid products and the reaction of oxygen with the remaining carbons to form CO as the predominant gas product. The catalytic system comprises CuO nanopowder, I_2_, and base, thereby enabling the reaction to proceed under mild conditions using air as the oxidant. The unpurified gas stream (containing CO) may be directly used in carbonylation reactions. We believe that this method, which simultaneously transforms lignin into value‐added liquid and gas products under mild conditions, expands the scope of lignin chemistry for the preparation of high‐value products such as pharmaceuticals and other commodity chemicals.

## Results and Discussion

Cu‐based catalysts have been shown to catalyze the oxygenation of the C−C bonds in lignin (leading to depolymerization of lignin).^[15]^ Therefore, we hypothesized that performing the reaction in the presence of a carboxylic acid would result in capture of the methoxy groups to afford methyl esters. Initial studies employing *p*‐phenylbenzoic acid **1** **a** and beech wood lignin as the methyl source, which is rich in methoxy groups,^[1a]^ were conducted to screen different Cu catalysts, co‐catalysts, and additives (Table 1). Commercially available CuO nanopowder was more effective than Cu_2_O and various Cu salts when applied in the presence of phenanthroline (ligand, L1) and I_2_, although the methyl *p*‐phenylbenzoate product **1** **b** was initially obtained in only moderate yield (yield 46 %, entry 5). With the exception of CuSO_4_ and Cu(OAc)_2_, the Cu catalysts evaluated displayed similar activity in the decomposition of lignin to CO (yields 68–77 %, entries 1, 4, and 5; 1 g of beech lignin affords 453 mg of CO, yield 72 %, entry 5). Various ligands were evaluated that were less effective than phenanthroline (entries 6 and 7), with the exception of 4,7‐dimethoxyphenanthroline (Ophen, L4) with the yield of **1** **b** increasing to 71 % (entry 8). The electron‐rich Ophen ligand L4 increases the electron density at the surface of the CuO nanocatalyst, which influences the adsorption/desorption of the substrates, intermediates, and products,^[16]^ and promotes the activation of O_2_.^[17]^ The reaction hardly proceeds in the absence of I_2_ (entry 9), and other iodine‐containing additives are relatively inefficient for promoting the reaction (entries 10–12). A significant enhancement in the yield of **1** **b** was achieved by increasing the amount of I_2_ to 60 mol % (yield 97 %, entry 13). It should be noted that stoichiometric quantities of methoxy groups (based on the amount of lignin used) affords **1** **b** in 90 % yield (entry 14, see Table S1 for the carbon mass balance of this entry). Moreover, the reaction hardly proceeds under a N_2_ atmosphere (yield 3 %, entry 15) and no reaction occurred in the absence of lignin (yield 0 %, entry 16), confirming that the methoxy groups on ligand L4 do not participate in the reaction. Optimization of various solvents and their combinations, temperatures, bases, and feed ratios are described in detail in the Supporting Information (Tables S2–S13). The CuO catalyst is insoluble in the reaction solvent and is easily isolated after reaction by filtration, and was reused several times without showing a loss of activity (Figure S1). No soluble Cu species were detected using mass spectrometry (Figure S2), which further confirms the insolubility of the CuO catalyst. Ligand L4 could also be regenerated (Figure S3).

**Table 1:**
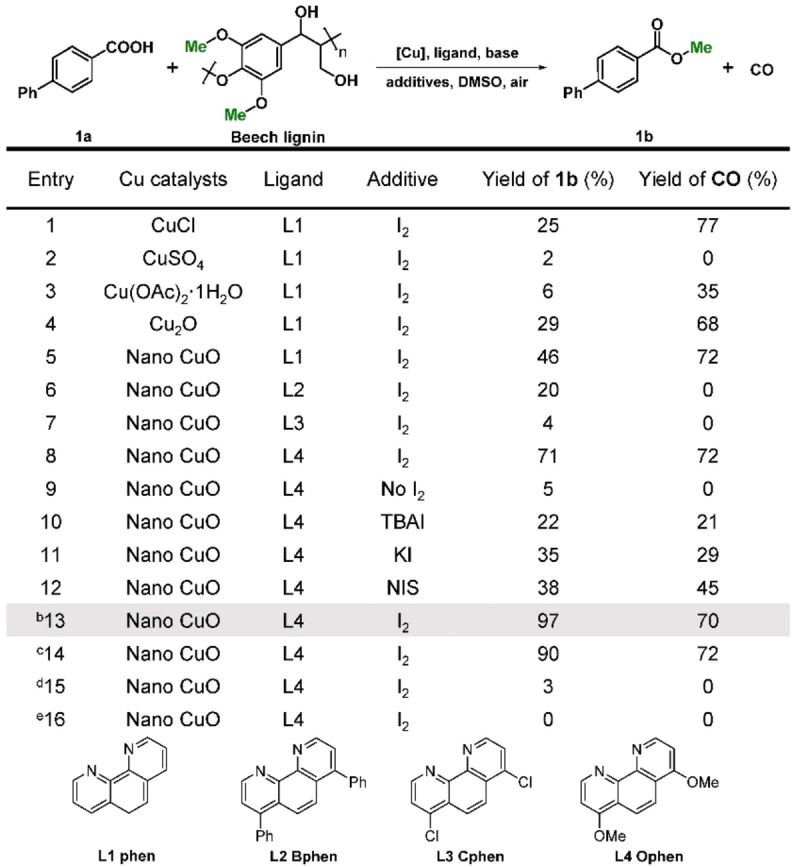
Optimization of reaction parameters.^[a]^

Reaction conditions: [a] *p*‐phenylbenzoic acid (0.2 mmol), beech lignin (1.5 equiv based on content of methoxy groups, 45 mg, content of methoxy group=6.70 mmol g^−1^ lignin), Cu catalyst (0.06 mmol), ligand (0.02 mmol), K_2_CO_3_ (0.4 mmol), additive (0.08 mmol), DMSO (2 mL), 140 °C, 10 h, air balloon (1 atm). [b] I_2_ (0.12 mmol). [c] 1 equiv based on methoxy groups of beech lignin (30 mg). [d] Reaction in a sealed tube. Before sealing the tube, the reaction mixture was purged with N_2_ gas for 2 h. [e] No lignin. Yields were calculated based on the *p*‐phenylbenzoic acid used and determined by gas chromatography (GC). Details of quantitative methods are provided in the Supporting Information. TBAI=tetrabutylammonium iodide. NIS=N‐Iodosuccinimide.

**Figure 1:**
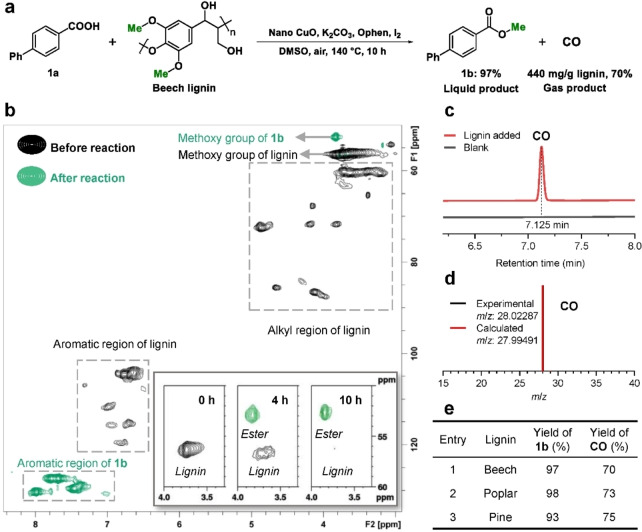
Characterization of the products generated from the controlled catalytic degradation of lignin in the presence of *p*‐phenylbenzoic acid. a) Reaction scheme. b) Selected regions of the HSQC NMR spectra of the reaction mixture before (black contour, 0 h), during (4 h) and after reaction (10 h) and liquid reaction mixture (green contour). The insert shows HSQC NMR spectra of the methoxy groups as a function of time. c) CO detected by GC. Control experiment without lignin. d) Mass spectrum of the gas confirming the formation of CO. e) Table giving the product yields obtained with other types of lignin. Reaction conditions: *p*‐phenylbenzoic acid (0.2 mmol), lignin (1.5 equiv based on content of methoxy groups), nano CuO (0.06 mmol), Ophen L4 (0.02 mmol), K_2_CO_3_ (0.4 mmol), I_2_ (0.12 mmol), DMSO (2 mL), air balloon (1 atm), 140 °C, 10 h. Liquid and gas products were quantified by GC.

The reaction was investigated using 2D short‐range ^13^C‐^1^H correlation (HSQC) NMR spectroscopy (Figure 1b; Figure S7). Characteristic resonances of lignin, including the methoxy groups, alkyl region, and aromatic region are apparent before reaction (black contours). After reaction (green contours), all the characteristic resonances of native lignin are essentially no longer present, indicating near‐complete decomposition of lignin. The new signals may be assigned to methyl *p*‐phenylbenzoate, corroborating the GC results. A HSQC NMR spectrum was also recorded after 4 h (insert in Figure 1b), revealing the progressive consumption of the methoxy groups in lignin and concomitant formation of methyl *p*‐phenylbenzoate. Full HSQC spectra are shown in Figure S7. Analysis of gaseous products was conducted using GC and GC‐MS (Figure 1c, d; Figures S8 and S9), with CO obtained in a yield of 70 % (440 mg/g beech lignin).

The chemical composition and structure of lignin depends on the type of plant and on the extraction method. Gymnosperm and angiosperm have different compositions of coniferyl and sinapyl units, which directly determine the amount of methoxy groups in lignin.[^1^, ^18^] Hence, another hardwood lignin was employed (i.e., poplar), which has a similar composition and structure to beech lignin (Figure 1e). *p*‐Phenylbenzoic acid **1** **a** was successfully methylated using poplar lignin and gave the corresponding ester product **1** **b** and CO in similarly high yields to beech lignin (i.e., 98 % and 73 %; 460 mg/g poplar lignin), respectively. Pine wood, a representative of a gymnosperm, mainly contains the coniferyl unit. Nonetheless, pine lignin also affords **1** **b** and CO in 93 % yield and 75 % (471 mg/g pine lignin), respectively. Thus, different kinds of lignin can be totally decomposed and upgraded to the methyl ester as the only liquid product and CO as the main gas‐phase product. In contrast to many oxidative reactions of lignin,[^4d^, ^6a^] solid residues were not observed after reaction, indicating complete conversion of the lignin substrate (also evidenced by HSQC NMR spectroscopy).

Encouraged by the successful application of lignin as a selective methylation reagent for the transformation of **1** **a** into **1** **b**, the reaction was expanded to other carboxylic acids (Table 2). Initially the substrate scope of other aryl acids was investigated with a diverse range of substituted benzoic acids undergoing methylation in very high yield to produce the corresponding ester products (yields 90–99 %, **2** **b**–**4** **b**). However, *o*‐anisic acid (**5** **a**) was transformed to the ester product (**5** **b**) with a yield of only 71 %. The high degree of functional group tolerance allows privileged scaffolds present in pharmaceuticals to be methylated, such as indole‐substituted (**6** **a**), *p*‐cyanophenoxy‐substituted (**7** **a**), and polyhalogenated (**8** **a**) benzoic acids, to afford the expected esters in high yield (yields 85–98 %). Terephthalic acid (**9** **a**) also reacts efficiently under the reaction conditions to afford dimethyl terephthalate in high yield (yield 83 %). Various heterocyclic aromatic compounds, including furan (**10** **a**), benzothiophene (**11** **a**), and an indole derivative (**12** **a**) were evaluated in the reaction and generated the expected methyl ester products in excellent yields (yields 96–98 %).

**Table 2:**
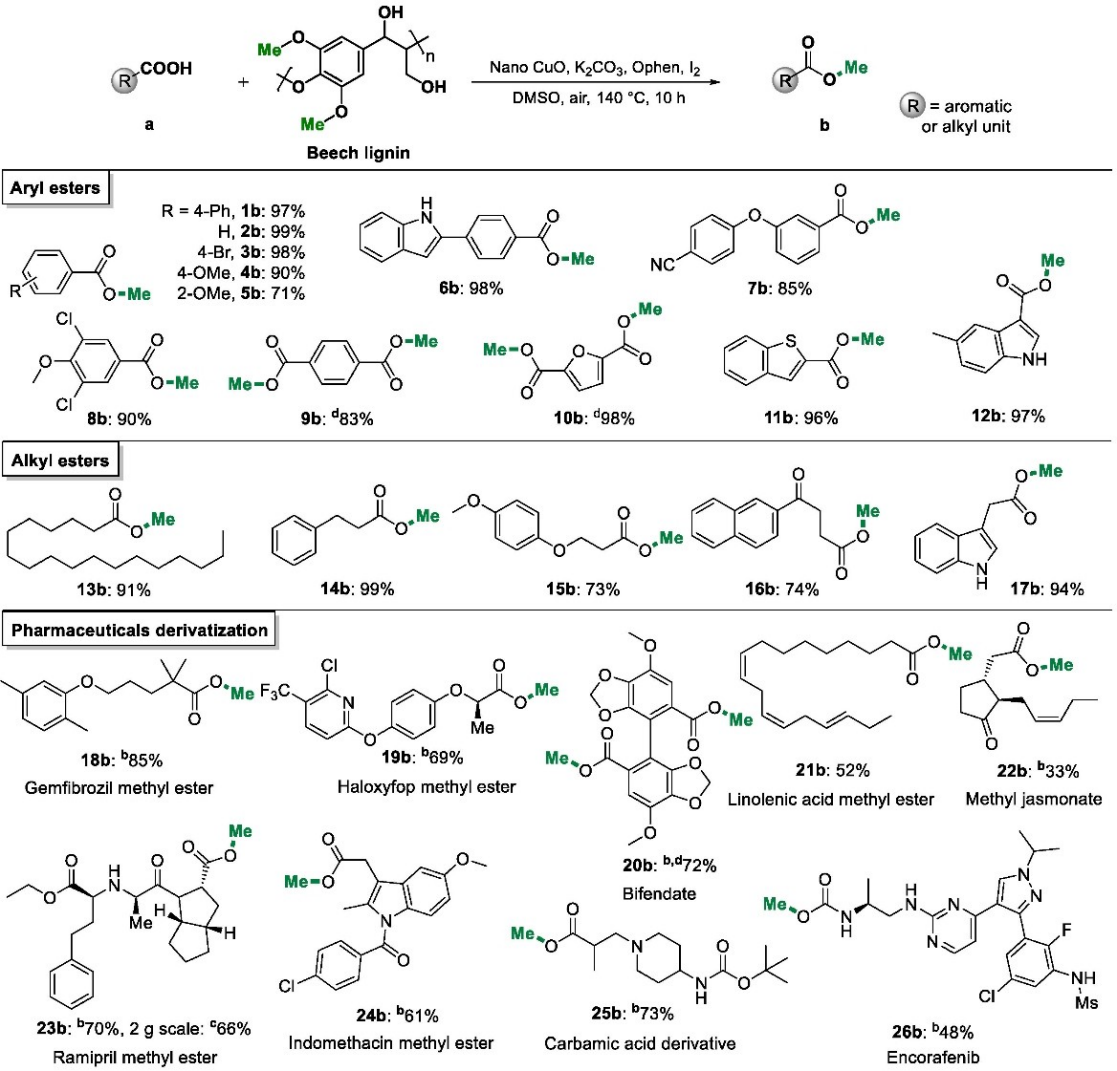
Evaluation of the substrate scope.^[a]^

Reaction conditions: [a] carboxylic acid (0.2 mmol), beech lignin (45 mg), nano CuO (0.06 mmol), Ophen L4 (0.02 mmol), K_2_CO_3_ (0.4 mmol), I_2_ (0.12 mmol), DMSO (2 mL), 140 °C, 10 h, air balloon (1 atm). Yields were determined by GC. [b] Isolated yields. [c] Scaled up to 2 g of carboxylic acid substrate. [d] Beech lignin (90 mg). Bn=benzyl; Ms=methanesulfonyl.

Next, a series of alkyl carboxylic acids, some of which have important biological functions, were evaluated. Methylation of the simple alkyl carboxylic acid (**13** **a**) and those with various aromatic functional groups, including benzyl (**14** **a**), phenoxy (**15** **a**), naphthoyl (**16** **a**), and indole (**17** **a**) proceed well under the reaction conditions (yields 73–99 %). The application of lignin as a methylation reagent was then applied to more challenging natural products and pharmaceutical compounds. Pharmaceuticals with alkyl or aryl ether linkages (genfibrozil, **18** **a**; haloxyfop, **19** **a**) tolerate the reaction conditions to afford the corresponding esters in isolated yields of up to 85 %. The natural rigid carboxylic acid (bifendate acid, **20** **a**) affords the corresponding ester with an isolated yield of 72 %. Linolenic acid (**21** **a**) and jasmonic acid (**22** **a**), natural alkenyl‐group‐containing compounds, react to produce the ester products in lower yields of 52 and 33 %, respectively. The lower yields may be attributed to the sensitivity of alkenyl groups to the oxidative conditions. Modified amide and amine derivatives (i.e., ramipril (**23** **a**), indomethacin (**24** **a**), and carbamic acid (**25** **a**)) were efficiently transformed to their corresponding ester products in good yield (yields 61–70 %). In addition, a 2 g scale reaction was successfully carried out on ramipril (**23** **a**), an angiotensin converting enzyme (ACE) inhibitor used to treat various diseases including heart failure and high blood pressure,^[19]^ affording the desired product in 66 % yield. Encorafenib (**26** **b**) is a BRAF inhibitor used to treat certain melanomas and contains a methyl ester unit.^[20]^ From the corresponding carboxylic acid precursor, encorafenib was isolated in 48 % yield. Overall, the broad scope of the lignin‐based methylation reaction, especially when applied to complex organic structures, implies that the route could be used in sustainable pharmaceutical synthesis since several approved drugs contain methyl ester groups.^[21]^

During the methylation reaction the lignin decomposes into gaseous products. The gas stream was collected and found to contain O_2_, N_2_, CO, and CO_2_ (Figures S8 and S9). The unpurified gas stream can be used directly for the carbonylation of alkyne, borate, and diol substrates, and for the carbonylative homocoupling of boronic acid (Figure S10), further valorizing the lignin carbon atoms.

**Figure 2:**
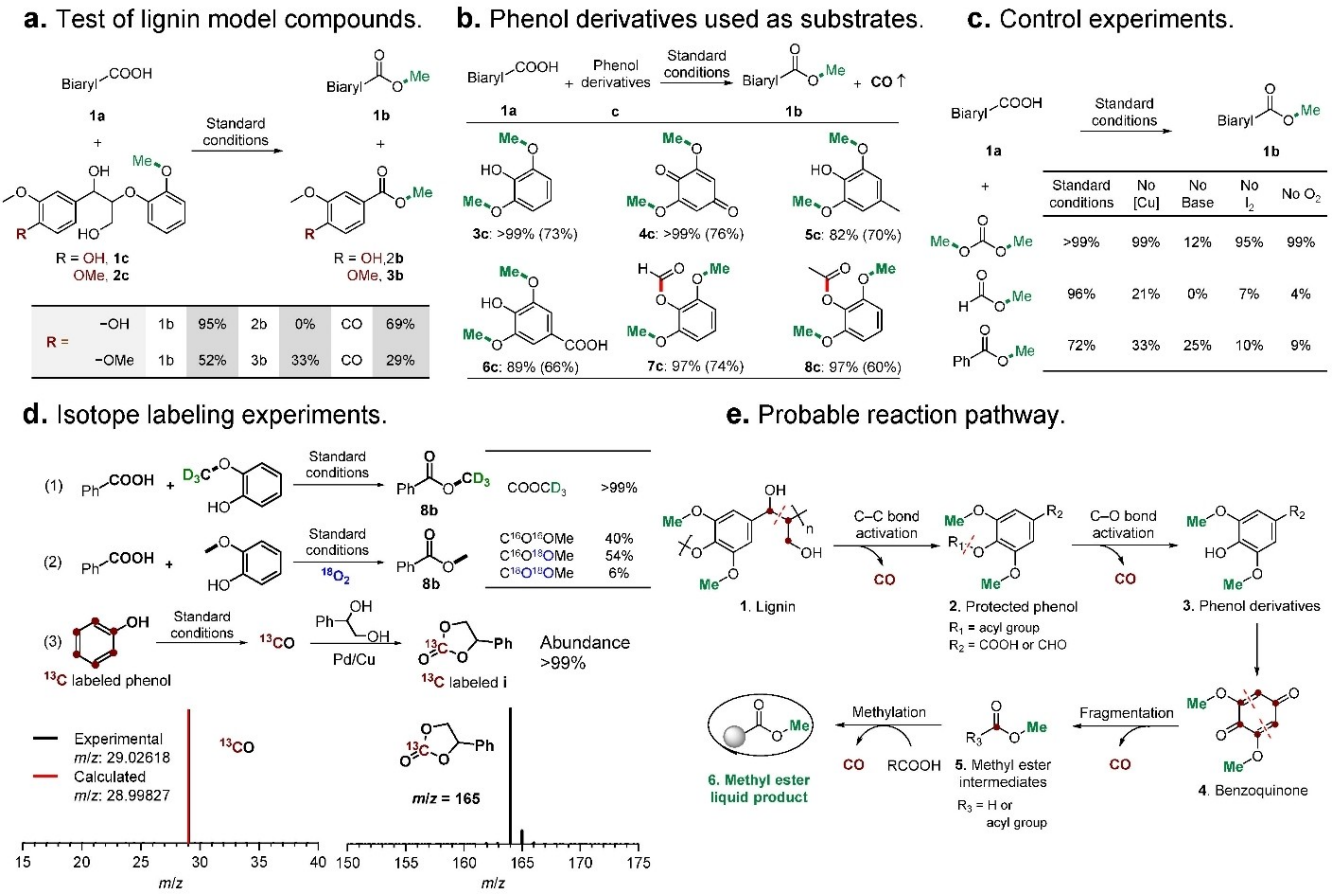
Mechanistic studies. a) Use of β–O–4 lignin model compounds as methylation reagents, 2 equiv. were used. b) Using phenol derivatives as methylation reagents, 1 equiv was used. Yields of the ester product **1** **b** and CO (in brackets) are given. c) Using esters as methylation reagents instead of lignin, 3 equiv were used. Methyl formate and dimethyl carbonate are volatile so the reaction was conducted in an autoclave charged with air (1 MPa). The yields of **1** **b** shown in the table (insert) demonstrate the role of the additives. d) Isotope‐labeling experiments were carried out using deuterated guaiacol (1), ^18^O_2_ instead of O_2_ (2), and ^13^C‐labeled phenol (3). EI‐MS of ^13^C‐labeled products. Abundance (%) of isotope is listed in the table (insert). e) Proposed reaction pathway. Standard reaction conditions: carboxylic acid (0.2 mmol), methylation reagent (1–3 equiv), nano CuO (0.06 mmol), Ophen L4 (0.02 mmol), K_2_CO_3_ (0.4 mmol), I_2_ (0.12 mmol), DMSO (2 mL), 140 °C, 10 h, air balloon (1 atm). Yield of ester is based on acid substrate and yield of CO is based on all carbons (alkyl and aromatic carbons) of compounds **3** **c**–**8** **c**, except the methoxy groups.

The reaction mechanism was initially probed using two types of β–O–4 lignin model compounds, one containing a phenolic hydroxyl group (**1** **c**) and the other with a methoxy group (**2** **c**). Both model compounds are effective methylation reagents (Figure 2a). Model compound **1** **c** affords methyl *p*‐phenylbenzoate **1** **b** as the only liquid product in 95 % yield and CO in 69 % yield, which implies that both phenyl rings in **1** **c** decompose during the reaction. When the methoxy group is at the *para* position (**2** **c**), two methyl esters, methyl veratrate (**3** **b**, 33 %) and **1** **b** (52 %), were detected as the liquid products. Previous reports on C−C bond activation using Cu catalysts under aerobic conditions indicate that the *p*‐methoxy‐substituted β–O–4 lignin model compound (**2** **c**) may be regarded as comprising two different aromatic fragments (benzyl ethanol and phenol), and following cleavage of the C_α_−C_β_ bond, afford veratric acid and substituted guaiacol as the oxidative products. The carbon atoms of the alkyl side chain react further to afford CO (Figure S11).[^15a^, ^15b^] Presumably, the substituted guaiacol is the methylation reagent. Two carboxylic acids (veratric acid generated from the cleavage of the C_α_−C_β_ bond in **2** **c** and the substrate **1** **a**) capture the methoxy group of substituted guaiacol to produce two kinds of methyl esters (**1** **b** and **3** **b**) with guaiacol then decomposing to form additional CO. Fragmentation and intramolecular esterification of **2** **c** was further confirmed as the methyl ester products were generated from β–O–4 lignin model compounds, which do not contain a phenolic hydroxyl group (similar to **2** **c**) in the absence of **1** **a** (Figure S12).

When the hydroxyl group is at the *para* position (**1** **c**), a benzyl ethanol fragment was also considered as a guaiacol derivative and used as a methylation reagent and for production of additional CO. Lignin contains **1** **c** units in the structure and transforms to methoxyphenol derivatives via C_α_−C_β_ bond activation, which corresponds to the methylation agents. Various types of methoxyphenol derivatives were reacted with **1** **a** under the standard reaction conditions (Figure 2b; Figure S13). Unprotected (**3** **c**–**6** **c**) and protected (**7** **c**–**8** **c**) phenol derivatives react smoothly with **1** **a** to afford **1** **b** and CO in high yield, confirming that highly active phenol derivatives generated from decomposition of lignin are key intermediates in the reaction.

Over‐oxidation of methoxy‐substituted phenols has been shown to lead to fragmentation of the aromatic ring to produce CO, with the methoxy groups forming methyl formate intermediates.^[22]^ Three representative esters (i.e., dimethyl carbonate, methyl formate, and methyl benzoate), were used as model methylation reagents to further probe the mechanism (Figure 2c). All three methyl esters react with **1** **a** to afford **1** **b** in high yield (yields 72–99 %; further details are provided in Figure S14). In principle, they could undergo three different reaction pathways (Figure S15). Detailed control experiments were conducted to verify the reaction pathways under the standard reaction conditions. It was found that dimethyl carbonate acts as the methylation reagent and a catalyst and other reagents are not required. With methyl formate and methyl benzoate, Cu catalyst, base, I_2_, and O_2_ are all indispensable for efficient reaction. Taken together, these results imply that methylation of carboxylic acids with methyl formate or methyl benzoate, as well as lignin, may share a similar mechanism involving CH_3_−O bond activation. For relatively stable methyl benzoate, activation of the CH_3_−O bond is not easily achieved by decomposition, excluding the methylation pathway involving decomposition of methyl formate. However, the CH_3_−O bond in methyl benzoate could be activated by oxidative addition to the Cu catalyst (pathway a, Figure S15), which is consistent with previous reports concerning heterogeneous Cu‐catalyzed cross‐coupling reactions.[^16b^, ^23^]

To confirm the reaction mechanism, isotope‐labeling experiments were carried out to probe the fragmentation of phenols (Figure 2d; Figures S16–S19). Using guaiacol with a deuterated methoxy group, the methyl group in methyl benzoate (**8** **b**) is deuterated (>99 %), confirming that the origin of the methyl group is from the methoxy group of guaiacol, which may be extrapolated to the abundant methoxy groups in lignin (equation 1, Figure 2d). Unexpectedly, ^18^O‐labeled guaiacols are transferred to non‐labeled ester products (Figures S16 and S17). Hence, the oxygen atoms in **8** **b** originate from the substrate or O_2_, further ruling out that methyl formate intermediates undergo decomposition pathway b (Figure S15), mediated by methanol intermediates. Using ^18^O_2_ as the oxidant affords a mixture of ^18^O‐labeled and non‐labeled products (equation 2, Figure 2d), demonstrating that oxygen exchange with the substrates and/or intermediates occurs. The isotopic abundance of the unreacted acids was investigated at the midpoint of the reaction with ca. 50 % of the oxygen atoms being labeled with ^18^O (Figures S18e and S19e). Hence, oxygen exchange between O_2_ and the substrate takes place prior to reaction with the methylation reagent and does not originate from the methylation reagent. This finding agrees with previous studies, which show that Cu catalysts promote oxygen exchange between O_2_ and carboxylic acids (Figure S20).^[24]^ To confirm the source of CO, ^13^C‐labeled phenol was employed in the reaction and ^13^CO was identified by GC‐EI‐MS (Eq. (3), Figure 2d). The ^13^CO‐containing gas mixture was also upgraded by carbonylation of a diol substrate and ^13^C‐labeled carbonate **i** was produced, which further shows that the carbon atoms of CO are derived from the phenyl carbon of phenol. Since lignin monomers comprise C9 units, propane‐1,3‐diol (**l**) and 1‐phenylpropane‐1,3‐diol (**m**) were introduced as model substrates into the standard reaction and were shown to afford CO in high yield (yields 80–82 %, Figure S21). These results demonstrate that both the phenyl and alkyl carbons of lignin can be transformed into CO.

Based on these studies and relevant literature, a tentative mechanism (Figure 2e) may be proposed. The first step involves the cleavage of propyl linkages to depolymerize lignin **1** into methoxy‐phenol monomers **3** via a well‐ordered, C−C and C−O bond activation and functionalization cascade. The carbon atoms of the propyl side chains are then transformed into CO. The resulting phenol monomers undergo further oxidative decomposition to form additional CO and methyl ester intermediates **5** mediated via benzoquinone intermediate **4** (Figures S22 and S23). The methyl ester intermediates formed are captured by the carboxylic acid substrate to yield the methyl ester product **6**. Further details of the reaction mechanism are provided in the Supporting Information (also see Figures S11, S22, and S23).

## Conclusion

An efficient catalytic system has been developed that enables the methoxy groups in lignin to methylate carboxylic acids to afford methyl esters. Notably, the C9 units in lignin decompose to afford CO, and no solid or liquid residues are observed after reaction. The method was used to methylate a wide and diverse range of carboxylic acids, including aryl, alkyl, and complex pharmaceutical substrates. The CO produced could be used in carbonylation reactions without further purification. Detailed mechanistic studies demonstrated that a well‐ordered oxidative cascade of the C−C and C−O bonds takes place. We believe that this new strategy for lignin valorization could find applications in the production of many valuable organic compounds, including pharmaceuticals.

## Conflict of interest

The authors declare no conflict of interest.

1

## Supporting information

As a service to our authors and readers, this journal provides supporting information supplied by the authors. Such materials are peer reviewed and may be re‐organized for online delivery, but are not copy‐edited or typeset. Technical support issues arising from supporting information (other than missing files) should be addressed to the authors.

Supporting Information

## Data Availability

The data that support the findings of this study are available in the Supporting Information of this article.
