# Supplementary material for: Simultaneous Generation of Methyl Esters and CO in Lignin Transformation
Source: Angew Chem Int Ed Engl. 2022 Sep 1;61(40):e202209093. doi: 10.1002/anie.202209093 (PMC9826404; doi:10.1002/anie.202209093)
Supplement: Supplementary file 1 — Supporting Information [file ANIE-61-0-s001.pdf]

Angewandte Chemie
Eine Zeitschrift der Gesellschaft Deutscher Chemiker

Supporting Information

Simultaneous Generation of Methyl Esters and CO in Lignin Transformation

M. Liu, B. Han, P. J. Dyson**

Experimental part

Chemicals

All chemicals were used directly without any post-processing unless otherwise specified. Cu salts and ligands were purchased from Acros or Sigma-Aldrich. CuO nano powder (aerodynamic particle sizer, APS, 30-50nm, 99.9% metals basis) was provided by Alfa Aesar. Solid organic bases were purchased from Acros, and liquid organic bases were obtained from Sigma-Aldrich. Additives including iodine, N-iodosuccinimide, N-bromosuccinimide, tetrabutylammonium iodide, tetrabutylammonium bromide and 4A molecular sieve were purchased from Sigma-Aldrich. Organic acids and phenols were obtained from Sigma-Aldrich. Pharmaceuticals were obtained from MedChemExpress. ^{18}O and ^{2}H isotope agents were obtained from Cambridge Isotope Laboratories, Inc. Organsolv lignin was extracted as described below.

General procedure for methylation of carboxylic acids

The carboxylic acid substrate (0.2 mmol), beech lignin (1.5 equiv., 45 mg, content of methoxy groups is 6.70 mmol/g lignin), nano CuO (0.06 mmol), 4,7-dimethoxy-1,10-phenanthroline (Ophen L4, 0.02 mmol), K_2CO_3 (0.4 mmol), I_2 (0.12 mmol), DMSO (2 mL) and internal standard (byphenyl) were added into a two-neck flask equipted with a 1 L air ballon (if volatile ester products were generated the reaction was conducted in autocleave). Then the flask was heated to 140 °C for 10 h. The gaseous products were directly transfered to for gas chromatography (GC) and GC-MS analysis. The liquid reaction mixture was transfered to a two-phase system containing ethyl acetate and saturated aqueous NH_4Cl. Stoichiometric sodium hyposulfide (0.6 mmol) was added to consume the residual iodine. The ester products were extracted with ethyl acetate twice and combined. The organic phase was filtrated through silica gel to remove the ligand and metal salts before analysis by GC. Samples were needed further purified process by silica gel column chromatography to obtain products for NMR spectroscopy or to obtain isolated yields.

For recycling the CuO catalyst, the CuO catalyst is dispersed in the aqueous phase. After centrifuging, the solid CuO could be recycled by washing with water, acetone and ethyl acetate, each for 3 times, followed by drying in oven at 80 °C over night.

Synthesis of lignin model compounds

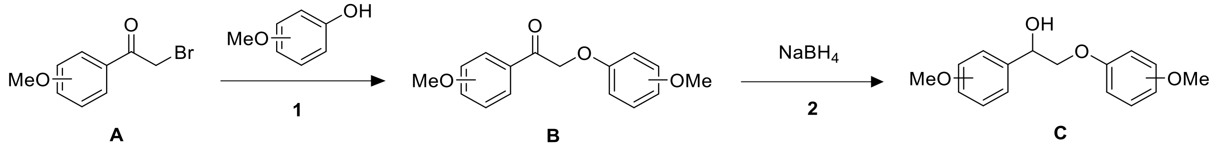

Preparation of β-phenoxy substituted 1-phenyl ethanol type lignin oligomers[1]: (**1**) phenols (65 mmol), 2-bromoacetophenone (**A**, 60 mmol), K_2CO_3 (90 mmol), and acetone (250 mL) were added into a flask (500 mL). The reaction mixture was vigorously stirred and heated to reflux for 3 h. After reaction, the mixture was filtered and concentrated in *vacuo*. Crude ketone product (**B**) was purified by crystallization from ethanol. (**2**) Ketone **B** (15 mmol) was dissolved in MeOH (100 mL). Then the solution was cooled to 0 °C in ice bath, followed by slow addition of $NaBH_4$ (5 mmol). The reaction was stirred at 0 °C for 10 min and warmed to room temperature until the ketone was fully consumed. Then the reaction mixture was concentrated in *vacuo*. After adding saturated aqueous NH_4Cl (100 mL), the aqueous phase was extracted with ethyl acetate (3×100 mL). The organic phases were combined and washed with brine, dried with

anhydrous Mg_2SO_4, and finally concentrated in *vacuo* to give alcohols **C**. Further purification was conducted using silica gel column chromatography if necessary.

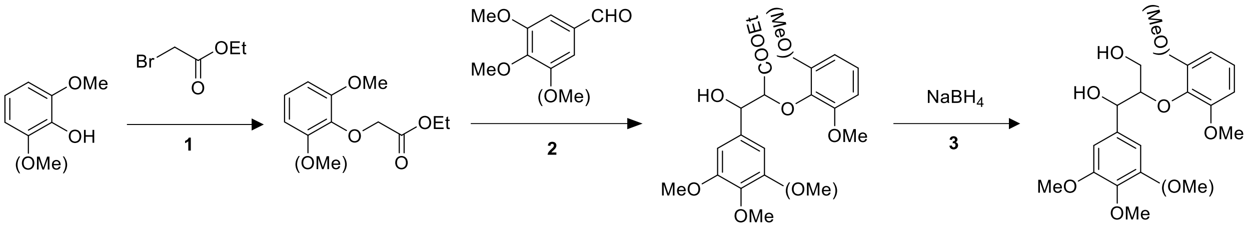

Preparation of the 1,3-diols type lignin oligomers[2]**:** 1,3-diols type lignin oligomers were prepared in three steps according to previous work. For example, 2-(2-methoxyphenoxy)-1-(3,4-dimethoxyphenyl)propane-1,3-diol (**D**) was prepared according to the following procedure, others were prepared in the same way from different starting materials.

(**1**) 2-Methoxyphenol (30 mmol), ethyl bromoacetate (30 mmol) and K_2CO_3 (60 mmol) were added into acetone (50 mL). The mixture was refluxed for 14 h until the substrates were fully consumed. After cooling to room temperature, the mixture was filtered and concentrated in *vacuo* to yield the solid product ketone **B** (ethyl 2-(2-methoxyphenoxy)acetate). (**2**) Veratraldehyde (10 mmol) and **B** (11 mmol) were dissolved in toluene (30 mL) and dried by azeotropic distillation thrice. Then the solid mixture was dissolved in dry THF (12 mL) and cooled to -78 °C in a dry ice/actone bath. Freshly prepared lithium diisopropylamide (LDA) solution (see below) was added dropwise into the mixture at -78 °C, and the reaction mixture was stirred at -78 °C for 2 h. After warming to 0 °C, the reaction was quenched with 100 mL saturated NH_4Cl aqueous solution. The aqueous phase was extracted with ethyl acetate (3×100 mL). The organic phase was washed with brine, dried with anhydrous Mg_2SO_4 and concentrated in *vacuo*. The crude product was purified using silica gel column chromatography, generating colorless oil **C**. (**3**) Product **D** was generated from the reduction of **C** with $NaBH_4$ as described above. 30 mol% $NaBH_4$ was used as reductant to reduce **C** into **D** in methanol solution at 0 °C. Further purification was conducted using silica gel column chromatography to obtain pure 1,3-diol lignin model compound **D**.

Fresh LDA solution was prepared by using the following procedure: when a solution of diisopropylamine (11 mmol) in dry THF (18 mL) was cooled to -78 °C, *n*-butyllithium solution (2.0 M in cyclohexane, 5.5 mL) was added at -78 °C. The LDA solution was used after warming to 0 °C.

Synthesis of functionalized phenols

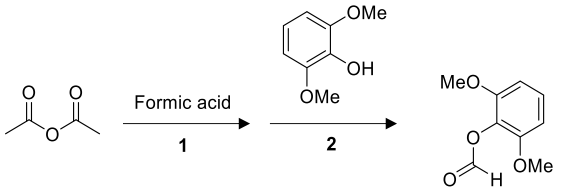

Formylation of phenols[3]: (**1**) formic acid (500 mmol, 5.0 equiv.) was added to acetic anhydride (400 mmol, 4.0 equiv.) and stirred at 60 °C for 1 h. (**2**) Phenol (100 mmol) and NaOAc (8.2 g, 100 mmol, 1.0 equiv.) were added to the mixture solution at room temperature and stirred for 3.5 h. After reaction, the mixture was diluted with toluene (100 mL), washed with H_2O (3×200 mL). The organic phase was

washed with brine, dried with anhydrous Mg_2SO_4, and finally concentrated in *vacuo* to give colorless oil composed of formylated phenols.

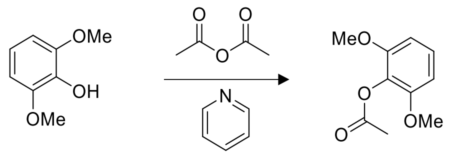

Acetylation of phenols[4]: pyridine was used as base to promote the acetylation of phenol. Phenol (100 mmol), acetic anhydride (200 mmol, 2.0 equiv.) and pyridine (60 mmol, 0.6 equiv.) were stirred for 2 h at 25 °C. The purification process was the same as that used for the formylation of phenols.

Synthesis of isotope labeled phenols

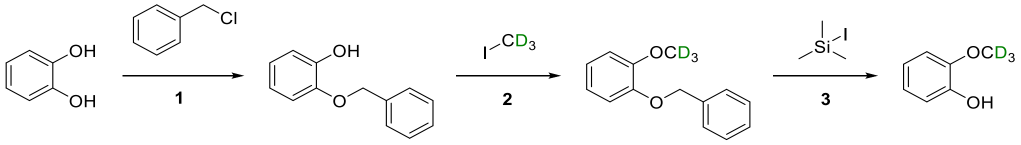

A B C D

Preparation of guaiacol with deuterated methoxy group[5]: (**1**) catechol **A** (20 mmol), benzyl chloride (20 mmol), anhydrous K_2CO_3 (30 mmol), and a catalytic amount of KI (1 mmol) were added to anhydrous acetone (100 mL) and refluxed for 10 h. After reaction, the reaction mixture was filtered, washed with brine, dried with anhydrous Mg_2SO_4, and concentrated in *vacuo* to give partially substituted phenol **B**. (**2**) Phenol **B** (10 mmol), CD_3I (30 mmol), and K_2CO_3 (30 mmol) were added to anhydrous acetone (100 mL) and refluxed for 10 h. After reaction, the mixture was filtered and concentrated in *vacuo* to give ether **C**. (**3**) Iodotrimethylsilane was used as a reductant to remove the benzyl protecting group. Ether **C** (10 mmol) was dissolved in CH_3CN (50 mL), heated to 50 °C under dry N_2. Iodotrimethylsilane (40 mmol) was added to the solution. The reaction was stirred at 50 °C for 20 min and quenched with methanol (10 mL). The reaction mixture was transfered to a two-phase system containing ethyl acetate (100 mL) and H_2O (100 mL). The aqueous phase was extracted with ethyl acetate (3×100 mL). The organic phase was washed with brine, dried with anhydrous Mg_2SO_4, and concentrated in *vacuo* to afford guaiacol **D**.

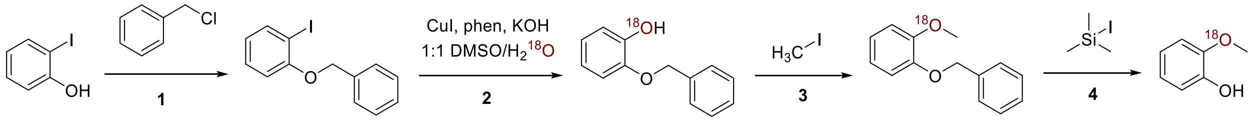

A B C D E

Preparation of guaiacol ^{18}O labeled methoxy group[5-6]: (**1**) the process to protect the hydroxyl group with benzyl chloride is the same as step 1 described above. (**2**) Ether **B** (3 mmol), CuI (0.3 mmol), 1,10-phenanthroline (phen, 0.9 mmol) and KOH (10 mmol) were added to DMSO/H_2O (1:1, 3 mL) under N_2, and reacted at 100 °C for 24 h in a sealed tube. After reaction, the mixture was transfered to two-phase system containing ethyl acetate (20 mL) and H_2O (20 mL). The aqueous phase was extracted with ethyl

acetate (3×20 mL). The organic phase was washed with brine, dried with anhydrous Mg_2SO_4, and finally concentrated in *vacuo* to give crude product. Further purification was conducted using silica gel column chromatography to afford pure phenol **C**. (**3**) Methylation of phenol **C** and (**4**) deprotection of **D** was the same as steps 2 and 3 described above.

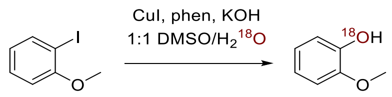

Preparation of guaiacol ^{18}O labeled hydroxyl group[6]: hydroxylation of 2-Iodoanisole to produce the labeled guaiacol is the same as step 2 above.

Extraction of organosolv lignin

Organic lignin was extracted according to previous reports.[2] Specifically, wood powders (<0.5 mm) were dried in *vacuo* at 80 °C for 24 h. The dried wood powder (200 g), 1,4-dioxane (1.5 L), and 2 M HCl (160 mL) were added to a three-necked flask. The reaction mixture was maintained under N_2 and heated to 110 °C and held for 60 min. After cooling to room temperature, the mixture was filtered and washed with 1,4-dioxane. The filtrate was concentrated into a sticky liquid (ca. 200 mL), which was subsequently mixed with acetone/water solution (9:1, ~250 mL). The resulting mixture was slowly added into rapidly stirred water (2.5 L) to genarate crude lignin powder. The resulting precipitate was flitered and re-dissolved in minimal acetone/methanol (9:1) solution and precipitated by slowly pouring it into rapidly stirred diethyl ether (2 L). The lignin precipitate was dried in *vacuo* at room temperature.

Characterization methods

The qualitative analysis of liquid products was conducted using GC-MS (Agilent 5975C-7890A, equipped with electron impact ionization mass spectrometer) and by comparison with authentic samples. The conversion of carboxylic acids and GC yields of corresponding ester products were quantitatively analyzed using GC (Agilent 7820, equipped with a hydrogen flame-ionization detector, full electric pneumatic control, inlet temperature 280 °C) based on internal standard curves and areas of integrated peak area. Quantitative analysis of gaseous products was conducted using GC (Agilent 7820, equipped with thermal conductivity detector) by comparison with authentic gas samples.

NMR spectra were recorded on Bruker Avance 400, 500 or 600 spectrometers equipped with 5 mm pulsed-field-gradient (PFG) probes. Pulse program of the Heteronuclear Single Quantum Coherence (HSQC) experiments was hsqcetgp. The spectral width of ^{13}C is from -10−210 ppm. The resonance band of tetramethylsilane (TMS) or solvents (DMSO or CD_3Cl) was used as the internal standard. Spectra were recorded at 298 K. For preparation of samples for HSQC experiments, DMSO-d_6 was used as solvent. After reaction, stoichiometric sodium hyposulfide (0.6 mmol) was added to consume the residual iodine. Then the mixture was flitered through silica gel to remove any undisolved Cu catalyst, base, and ligand. The filtrate was used directly for HSQC experiments. For quantitative ^{13}C NMR spectroscopy, chromium(III) acetylacetonate ($Cr(acac)_3$, ~2 mg) was added to the lignin solution (~100 mg, 0.5 mL DMSO-d_6) to completely relax the nuclei.

Quantitative methods

The molar concentration of the methoxy groups and other phenyl and alkyl carbons in lignin is determatined by quantitative ^{13}C NMR spectroscpy using trioxane as internal standard (Figs. S4-6), calculated with the specified weights and integration of the ^{13}C NMR spectra. In a typically reaction, lignin containing 1.5 equiv. of methoxy groups was used as the methylation reagent. For example, for a 0.2 mmol scale reaction, 45 mg beech lignin (methoxy group, 0.3 mmol; other carbons, 1 mmol) was used.

Yield (%) of liquid ester products is based on the carboxylic acid substrate:

$$Yield_{ester} = \frac{n_{ester}}{n_{acid}} \times 100\%$$

n_{ester} was determatined by GC based on internal standard curves and areas of integrated peak areas of internal standard and esters, or from the weight of the isolated products (specified high boiling point products).

Yield (%) of CO gas product was caculated using:

$$Yield_{CO} = \frac{n_{CO}}{n_{Carbon}} \times 100\%$$

n_{CO} was determatined by GC using an external standard method obtained from the measurement of several standard CO sample with different concentrations.

n_{ester}: mole of ester products. For typical reaction, ester methyl *p*-phenylbenzoate **1b** (0.195 mmol) was detected.

n_{acid}: mole of carboxylic acid. For typical reaction, *p*-phenylbenzoic acid **1a** (0.2 mmol) was used.

n_{CO}: mole of CO. For typical reaction, CO (19.5 mg, 0.70 mmol) was detected.

n_{Carbon}: mole of other carbons in lignin used. For typical reaction, lignin (45 mg) was used, which contains other alkyl and phenyl carbons (1.0 mmol).

Supporting results and discussions

Table S1. Carbon balance of reaction process.

	Lignin	Ester 1b	CO	CO_2	Others
Weight (mg)	30.2	38.4	13.6	6.1	
Carbon (mmol)	0.876	0.181	0.486	0.139	
C percentage (%)	100.0	20.7	55.5	15.8	8.0
Yield (%)		90.0	72.0	20.6	

Reaction conditions (Table 1, Entry 14): *p*-phenylbenzoic acid (0.2 mmol), beech lignin (1 equiv., 30 mg), nano CuO (0.06 mmol), Ophen (0.02 mmol), K_2CO_3 (0.4 mmol), I_2 (0.12 mmol), DMSO (2 mL), (1 atm), 140 °C, 10 h.

Table S2. Optimization of reaction conditions by screening Cu catalysts.

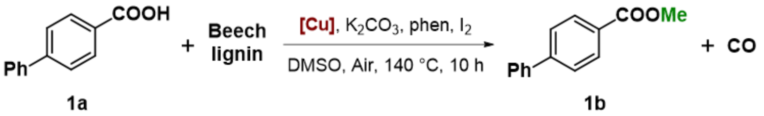

Entry	[Cu]	Yield of **1b** (%)	Yield of **CO** (%)
1	CuCl	25	78
2	CuBr·SMe_2	32	60
3	CuI	26	62
4	CuAc	7	9
5	Cu_2O	29	68
6	Cu_2S	31	42
7	CuO	36	67
8	$CuCl_2$	30	77
9	$Cu(OAc)_2 \cdot 1H_2O$	6	35
10	$Cu(NO_3)_2 \cdot 6H_2O$	28	67
11	$CuSO_4$	2	0
12	Nano CuO	46	72

Reaction conditions: *p*-phenylbenzoic acid (0.2 mmol), beech lignin (1.5 equiv., 45 mg, content of methoxy group is 6.70 mmol/g lignin), **Cu catalyst** (0.06 mmol), 1, 10-phenanthroline (phen, L1, 0.02 mmol), K_2CO_3 (0.4 mmol), I_2 (0.08 mmol), DMSO (2 mL), air balloon (1 atm), 140 °C, 10 h.

Nearly all the Cu salts catalyze the methylation of *p*-phenylbenzoic acid. However, CuO nano powder (aerodynamic particle sizer, APS, 30-50 nm, purchased from Alfa Aesar) was the most efficient catalyst.

Table S3. Optimization of reaction conditions by screening ligands.

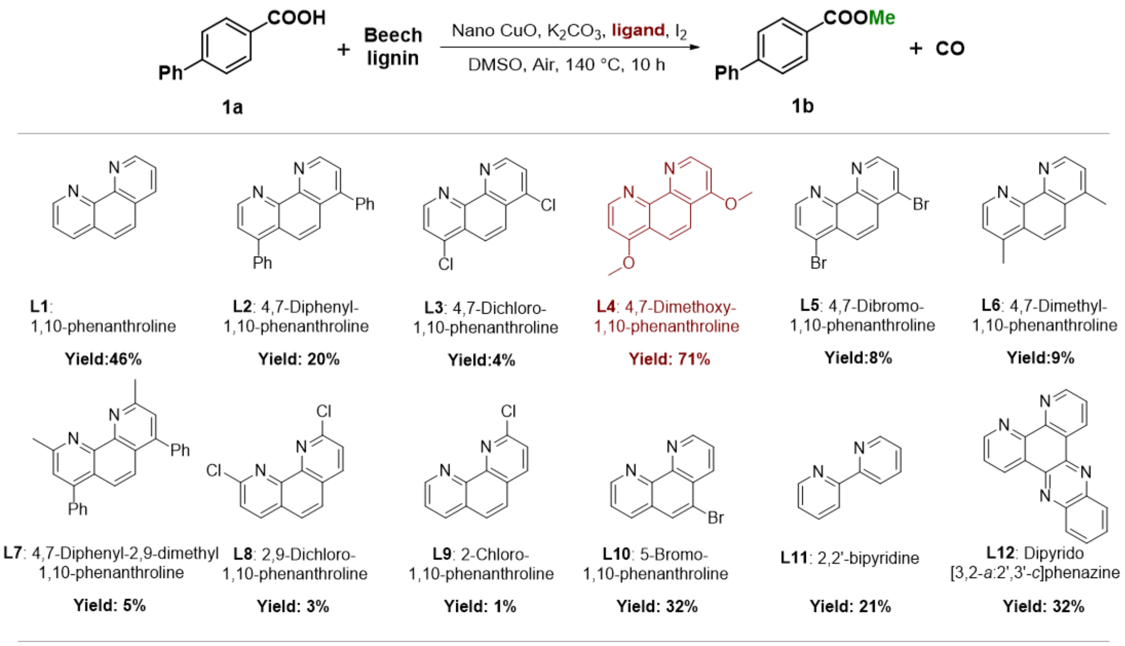

Reaction conditions: *p*-phenylbenzoic acid (0.2 mmol), beech lignin (1.5 equiv., 45 mg), nano CuO (0.06 mmol), **ligand** (0.02 mmol), K_2CO_3 (0.4 mmol), I_2 (0.08 mmol), DMSO (2 mL), air balloon (1 atm), 140 °C, 10 h.

Several modified phenanthroline ligands were tested. Methoxy group substituted phenanthroline (Ophen, L4) showed higher activity compared to the others (L1-3, L5-6). Bulky bathophenanthroline (L7) and other bidentate nitrogen ligands (L8-L12) showed poor catalytic activity.

Table S4. Optimization of reaction conditions by screening different bases.

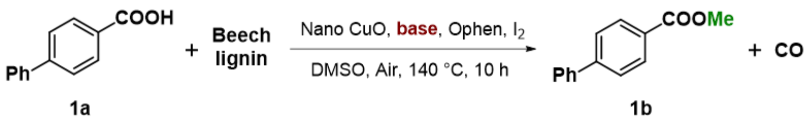

Entry	Base	Yield of **1b** (%)	Yield of **CO** (%)
1	K_2CO_3	71	72
2	Na_2CO_3	61	67
3	Cs_2CO_3	20	53
4	K_3PO_4	70	62
5	$NaHCO_3$	40	26
6	NaOH	35	9
7	KOH	16	3
8	KF	19	7
9	Triethylamine	8	0
10	Pyridine	0	0
11	1,8-Diazabicyclo[5.4.0]undec-7-ene	0	0

Reaction conditions: *p*-phenylbenzoic acid (0.2 mmol), beech lignin (1.5 equiv., 45 mg), nano CuO (0.06 mmol), Ophen (0.02 mmol), **base** (0.4 mmol), I_2 (0.08 mmol), DMSO (2 mL), air balloon (1 atm), 140 °C, 10 h.

Overall, inorganic bases are more efficient than organic bases. K_2CO_3, and K_3PO_4 have similar efficiency and K_2CO_3 was chosen as it also affords a higher yield of CO, has a lower hygroscopicity and is the least expensive.

Table S5. Optimization of reaction conditions by screening different solvents.

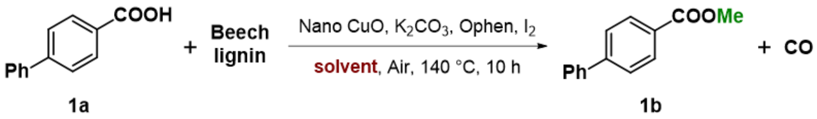

Entry	Solvent	Yield of **1b** (%)	Yield of **CO** (%)
1	DMF	70	42
2	DMSO	71	72
3	Dimethylacetamide	54	46
4	Chlorobenzene	17	0
5	Toluene	23	0
6	Dioxane	22	0
7	Anisole	14	38
8	CH_3CN	0	0
9	N-Methyl pyrrolidone	51	35
10	1,3-Dimethyl-2-imidazolidinone	43	33

Reaction conditions: *p*-phenylbenzoic acid (0.2 mmol), beech lignin (1.5 equiv., 45 mg), nano CuO (0.06 mmol), Ophen (0.02 mmol), K_2CO_3 (0.4 mmol), I_2 (0.08 mmol), **solvents** (2 mL), air balloon (1 atm), 140 °C, 10 h.

Table S6. Optimization of reaction conditions by screening additives.

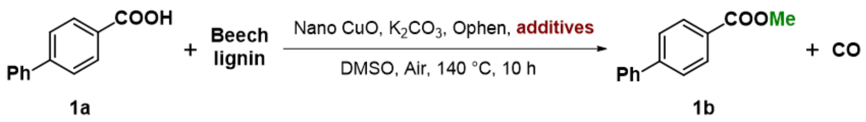

Entry	Additives	Yield of **1b** (%)	Yield of **CO** (%)
1		5	0
2	I_2	71	72
3	60% I_2	97	70
4	I_2 + 50 mg 4A	44	69
5	I_2 + 100 mg 4A	37	71
6	N-Iodosuccinimide	38	43
7	N-Bromosuccinimide	13	9
8	KI	35	63
9	NaI	33	60
10	LiI	38	57
11	Tetrabutylammonium iodide	22	21
12	Tetrabutylammonium bromide	11	8

Reaction conditions: *p*-phenylbenzoic acid (0.2 mmol), beech lignin (1.5 equiv., 45 mg), nano CuO (0.06 mmol), Ophen (0.02 mmol), K_2CO_3 (0.4 mmol), **additive** (0.08 mmol), DMSO (2 mL), air balloon (1 atm), 140 °C, 10 h.

Iodine containing additives have better activity than bromine containing additives. Their influence on the reaction is discussed in the mechanism part. Note that water absorbing agent molecular sieve (4A) decreases the yield of products.

Table S7. Optimization of reaction conditions by adjusting the amount of the nano CuO catalyst.

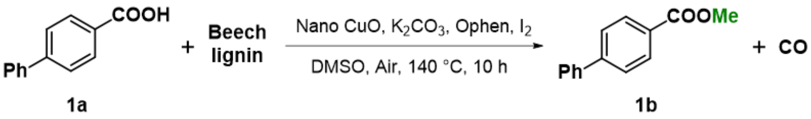

Entry	CuO	Yield of **1b** (%)	Yield of **CO** (%)
1	0%	8	0
2	10%	38	52
3	20%	79	73
4	30%	97	70
5	50%	97	57
6	80%	80	39

Reaction conditions: *p*-phenylbenzoic acid (0.2 mmol), beech lignin (1.5 equiv., 45 mg), nano CuO (**0-0.16** mmol), Ophen (0.02 mmol), K_2CO_3 (0.4 mmol), I_2 (0.12 mmol), DMSO (2 mL), air balloon (1 atm), 140 °C, 10 h.

Table S8. Optimization of reaction conditions by adjusting the amount of Ophen L4.

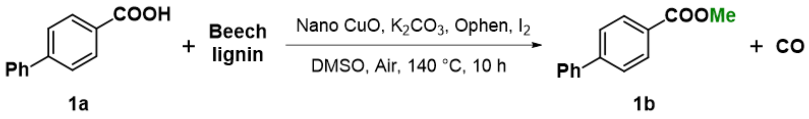

Entry	Ophen	Yield of **1b** (%)	Yield of **CO** (%)
1	0%	11	0
2	10%	97	70
3	30%	80	66
4	50%	59	66

Reaction conditions: *p*-phenylbenzoic acid (0.2 mmol), beech lignin (1.5 equiv., 45 mg), nano CuO (0.06 mmol), Ophen (**0-0.1** mmol), K_2CO_3 (0.4 mmol), I_2 (0.12 mmol), DMSO (2 mL), air balloon (1 atm), 140 °C, 10 h.

Table S9. Optimization of reaction conditions by adjusting the amount of K_2CO_3.

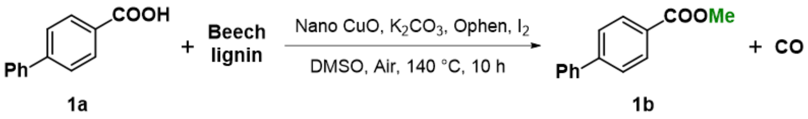

Entry	K_2CO_3	Yield of **1b** (%)	Yield of **CO** (%)
1	1 equiv.	40	57
2	2 equiv.	97	70
3	3 equiv.	86	52
4	4 equiv.	57	21

Reaction conditions: *p*-phenylbenzoic acid (0.2 mmol), beech lignin (1.5 equiv., 45 mg), nano CuO (0.06 mmol), Ophen (0.02 mmol), K_2CO_3 (**0.2-0.8** mmol), I_2 (0.12 mmol), DMSO (2 mL), air balloon (1 atm), 140 °C, 10 h.

Table S10. Optimization of reaction conditions by adjusting the amount of I_2.

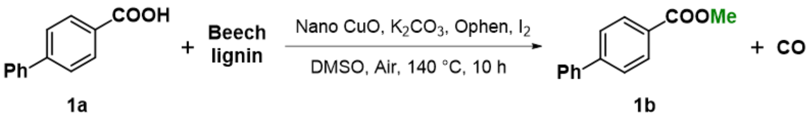

Entry	I_2	Yield of **1b** (%)	Yield of **CO** (%)
1	0%	5	5
2	20%	50	53
3	40%	71	69
4	60%	97	70
5	100%	98	62

Reaction conditions: *p*-phenylbenzoic acid (0.2 mmol), beech lignin (1.5 equiv., 45 mg), nano CuO (0.06 mmol), Ophen (0.02 mmol), K_2CO_3 (0.4 mmol), I_2 (**0-0.2** mmol), DMSO (2 mL), air balloon (1 atm), 140 °C, 10 h.

Table S11. Optimization of reaction temperature.

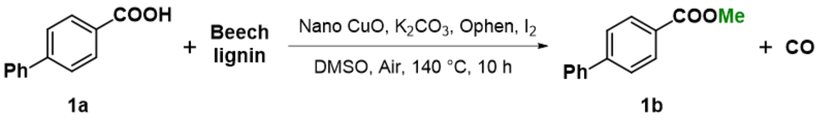

Entry	Temperature	Yield of **1b** (%)	Yield of **CO** (%)
1	100 °C	3	0
2	120 °C	43	58
3	130 °C	94	73
4	140 °C	97	70
5	150 °C	70	16

Reaction conditions: *p*-phenylbenzoic acid (0.2 mmol), beech lignin (1.5 equiv., 45 mg), nano CuO (0.06 mmol), Ophen (0.02 mmol), K_2CO_3 (0.4 mmol), I_2 (0.12 mmol), DMSO (2 mL), air balloon (1 atm), **100-150** °C, 10 h.

Table S12. Optimization of the reaction time.

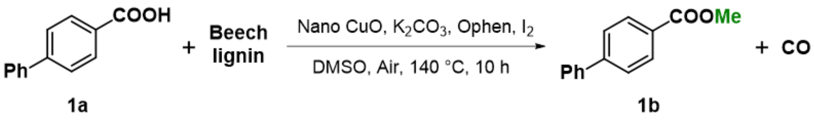

Entry	Reaction time	Yield of **1b** (%)	Yield of **CO** (%)
1	2 h	36	37
2	4 h	71	48
3	6 h	86	57
4	8 h	95	62
5	10 h	97	70
6	12 h	95	69

Reaction conditions: *p*-phenylbenzoic acid (0.2 mmol), beech lignin (1.5 equiv., 45 mg), nano CuO (0.06 mmol), Ophen (0.02 mmol), K_2CO_3 (0.4 mmol), I_2 (0.12 mmol), DMSO (2 mL), air balloon (1 atm), 140 °C, **2-12** h.

Table S13. Optimization of reaction conditions by adjusting the amount of lignin.

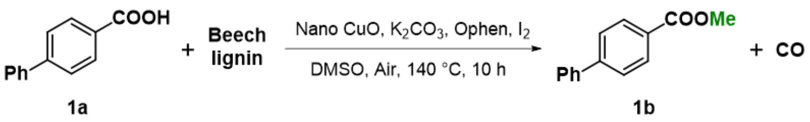

Entry	Lignin	Yield of **1b** (%)	Yield of **CO** (%)
1	1 equiv.	75	73
2	1.5 equiv.	97	70
3	2 equiv.	98	62

Reaction conditions: *p*-phenylbenzoic acid (0.2 mmol), beech lignin (1-2 equiv., calculated based on the content of methoxy group), nano CuO (0.06 mmol), Ophen (0.02 mmol), K_2CO_3 (0.4 mmol), I_2 (0.12 mmol), DMSO (2 mL), air balloon (1 atm), 140 °C, 10 h.

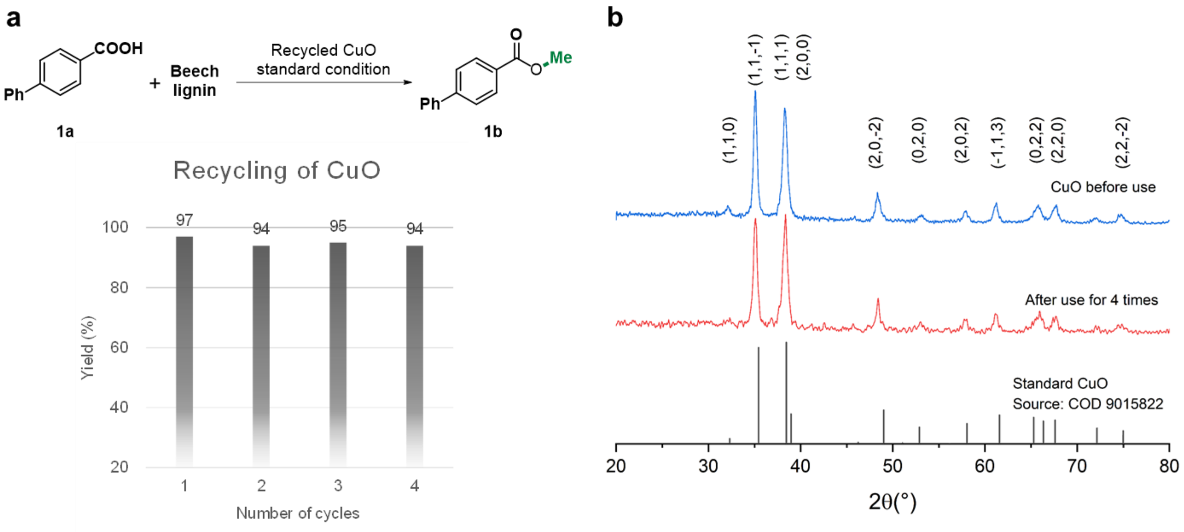

Figure S1. Recycling the CuO catalyst. (a) Reaction used in the catalyst recycling experiments (top) and reaction yields obtained during four cycles (bottom). (b) X-ray powder diffraction (XRD) patterns of the CuO catalyst before (blue) and after 4 consecutive reactions (red), and the standard diffraction peaks of CuO (source: COD 9015822). The peaks remain unchanged demonstrating the stability of the CuO catalyst during recycling. Reaction conditions: recycled CuO (0.06 mmol), **1a** (0.2 mmol), beech lignin (45 mg), Ophen L4 (0.02 mmol), K_2CO_3 (0.4 mmol), I_2 (0.12 mmol), DMSO (2 mL), 140 °C, 10 h, air balloon (1 atm).

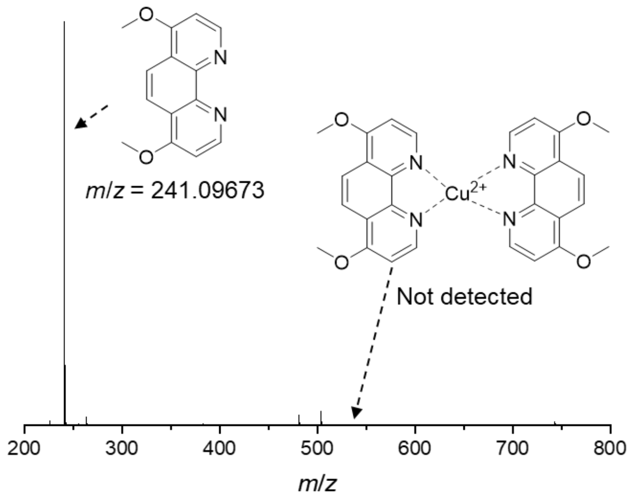

Figure S2. Electrospray ionization mass spectrometry (ESI-MS) spectra of reaction mixture. Reaction conditions: CuO (0.06 mmol), Ophen L4 (0.02 mmol), K_2CO_3 (0.4 mmol), I_2 (0.12 mmol), DMSO (2 mL), 140 °C, 10 h, air balloon (1 atm). After reaction, DMSO was almost removed by rotary evaporator. The mixture was diluted with methanol (20 mL) for the ESI-MS experiment.

No signals of Cu/ligand complex were detected by ESI-MS, which further confirms the insolubility of CuO in our reaction system. Although we cannot exclude the possibility that homogenous species are formed under the reaction conditions, ligands are known to improve the activity and/or selectivity of heterogeneous catalysis as they modify the sterics and electronics at the inorganic-organic interface.[7]

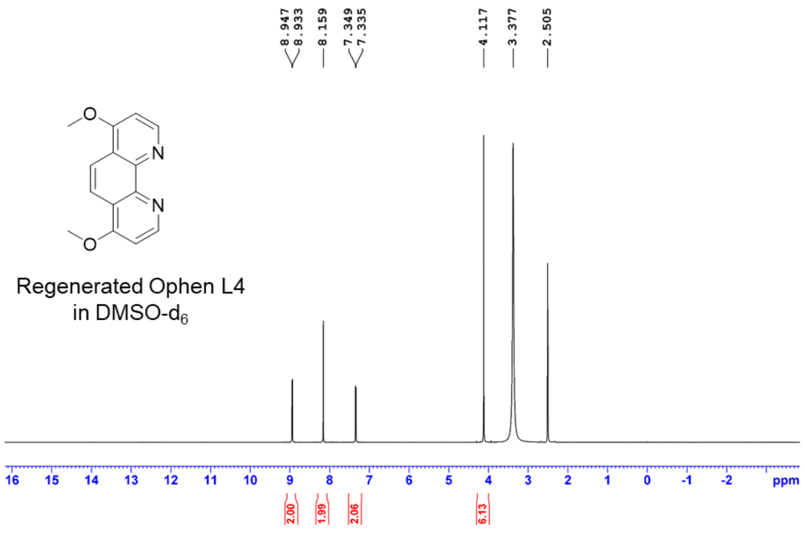

Figure S3. ^{1}H NMR spectrum of regenerated ligand 4,7-dimethoxyphenanthroline (Ophen, L4). Reaction conditions: CuO (0.6 mmol), **1a** (2 mmol), beech lignin (450 mg), Ophen L4 (0.2 mmol), K_2CO_3 (4 mmol), I_2 (1.2 mmol), DMSO (20 mL), 140 °C, 10 h, air balloon (1 atm).

For regeneration of L4, H_2O (50 mL) and CH_2Cl_2 (50 mL) was added to the reaction mixture after reaction. Sodium hyposulfide (3 mmol) was added to consume residual iodine. The CuO catalyst was removed by centrifugation. Then, the aqueous phase was extracted with CH_2Cl_2 (2 × 50 mL). The combined organic

phase was dried with Na_2SO_4, concentrated under vacuum to give dark brown solid organics. The solid organics was washed with (3 × 5 mL) and ethyl acetate (3 × 5 mL) to remove the organic products. Finally, the solid was purified by silica gel flash chromatography (2% MeOH in CH_2Cl_2 as eluent) to afford L4.

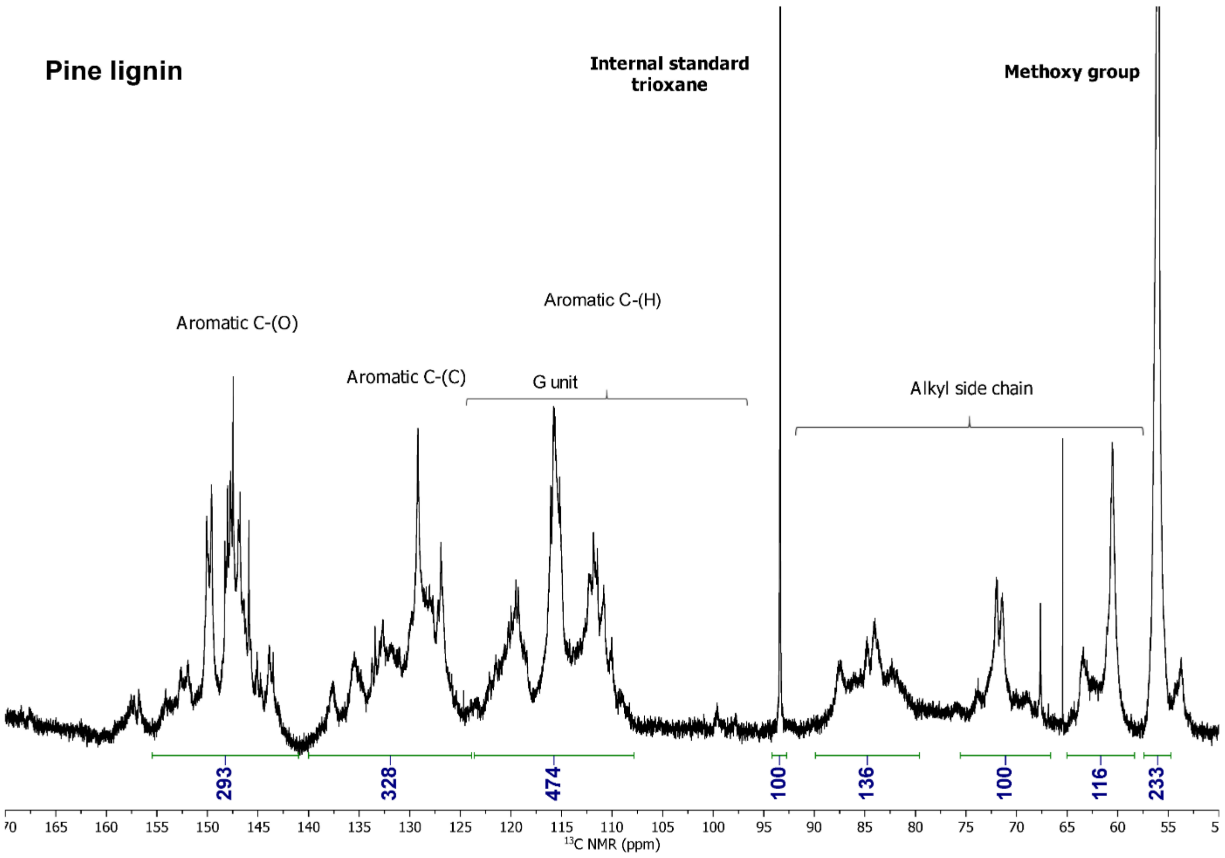

Figure S4. Quantitative ^{13}C NMR spectrum of pine wood lignin. Trioxane was used as an internal standard to calculate the content of the methoxyl groups. Pine lignin (103.6 mg), $Cr(acac)_3$ (2.3 mg), and trioxane (5.0 mg) in DMSO-d_6 (0.5 mL). The content of methoxyl group is 3.75 mmol/g lignin. The content of other carbon atoms is 23.26 mmol/g lignin.

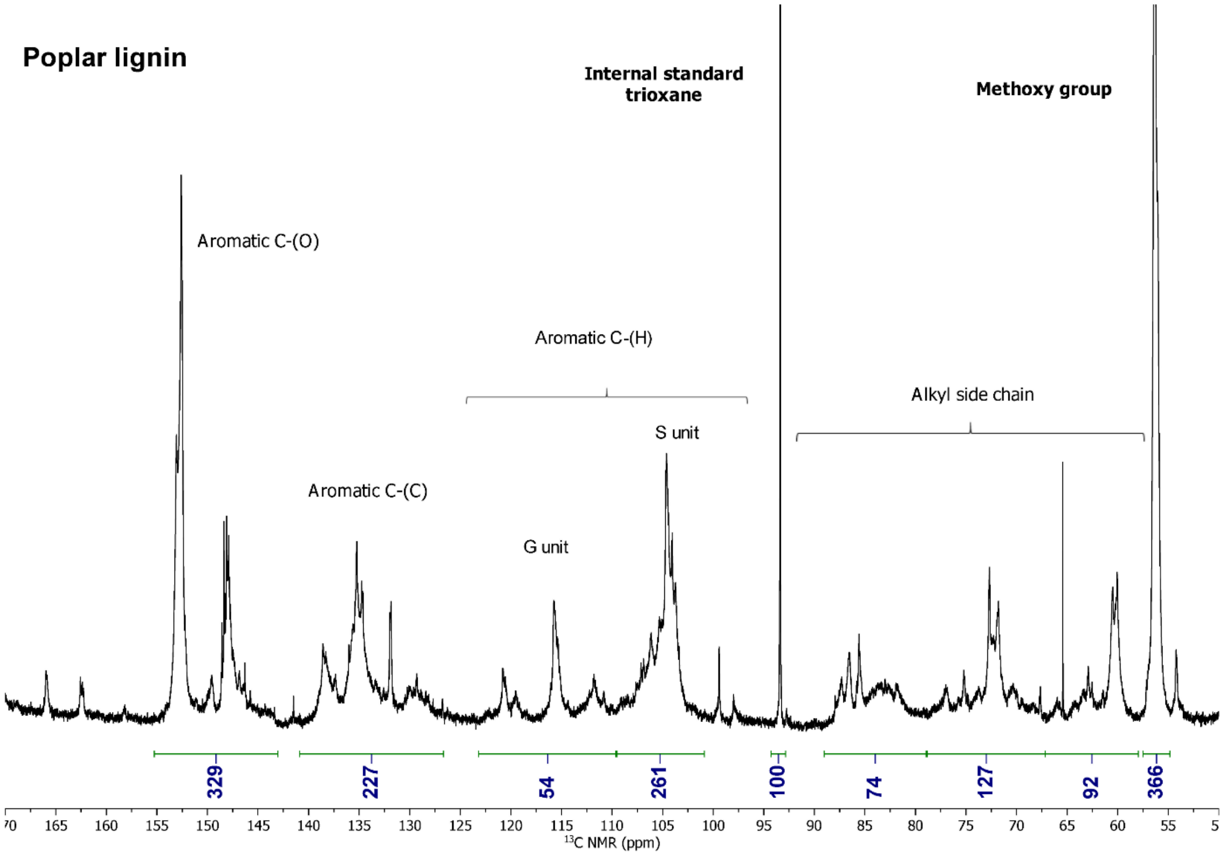

Figure S5. Quantitative ^{13}C NMR spectrum of poplar wood lignin. Trioxane was used as an internal standard to calculate the content of the methoxyl groups. Poplar lignin (103.2 mg), $Cr(acac)_3$ (2.3 mg), and trioxane (5.7 mg) in DMSO-d_6 (0.5 mL). The content of methoxyl group is 6.74 mmol/g lignin. The content of other carbon atoms is 21.43 mmol/g lignin.

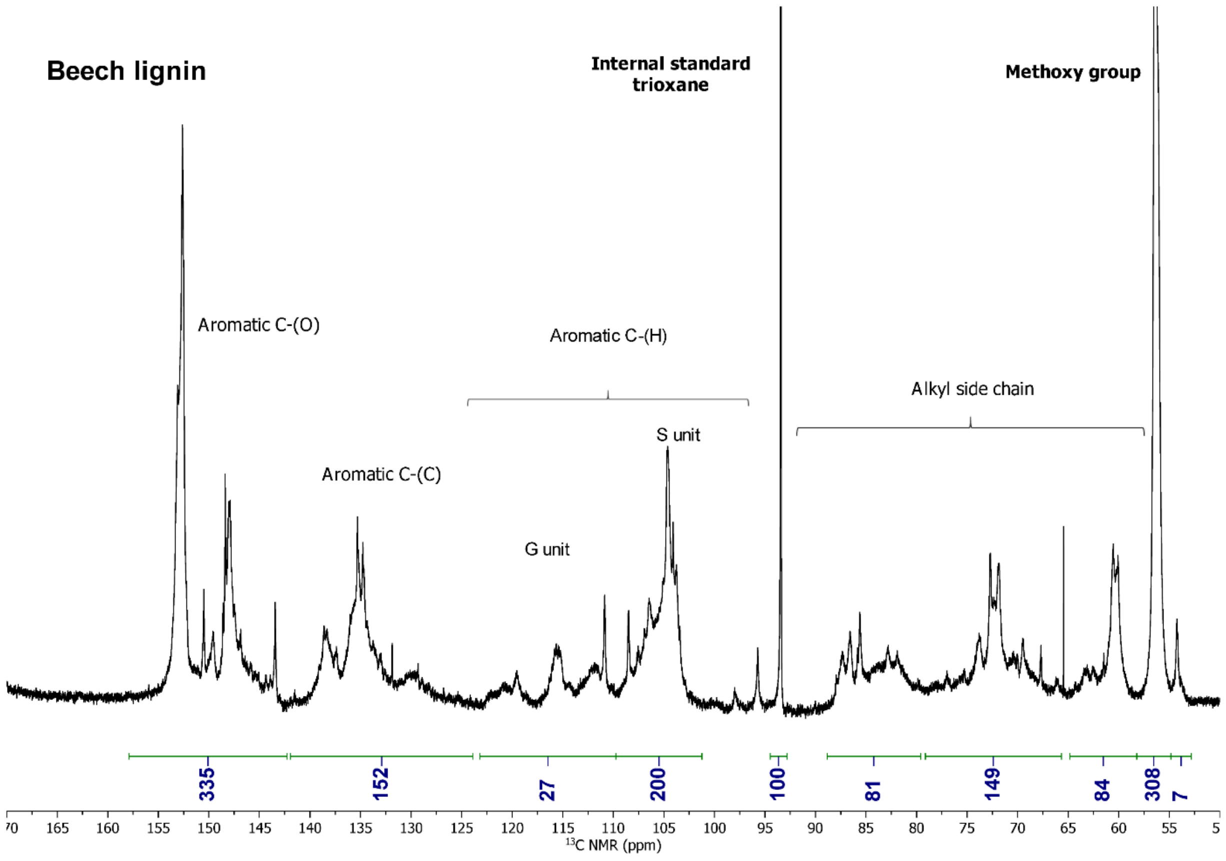

Figure S6. Quantitative ^{13}C NMR spectrum of beech wood lignin. Trioxane was used as an internal standard to calculate the content of the methoxyl groups. Beech lignin (108.8 mg), $Cr(acac)_3$ (3.7 mg), and trioxane (7.1 mg) in DMSO-d_6 (0.5 mL). The content of methoxyl group is 6.70 mmol/g lignin. The content of other carbon atoms is 22.51 mmol/g lignin.

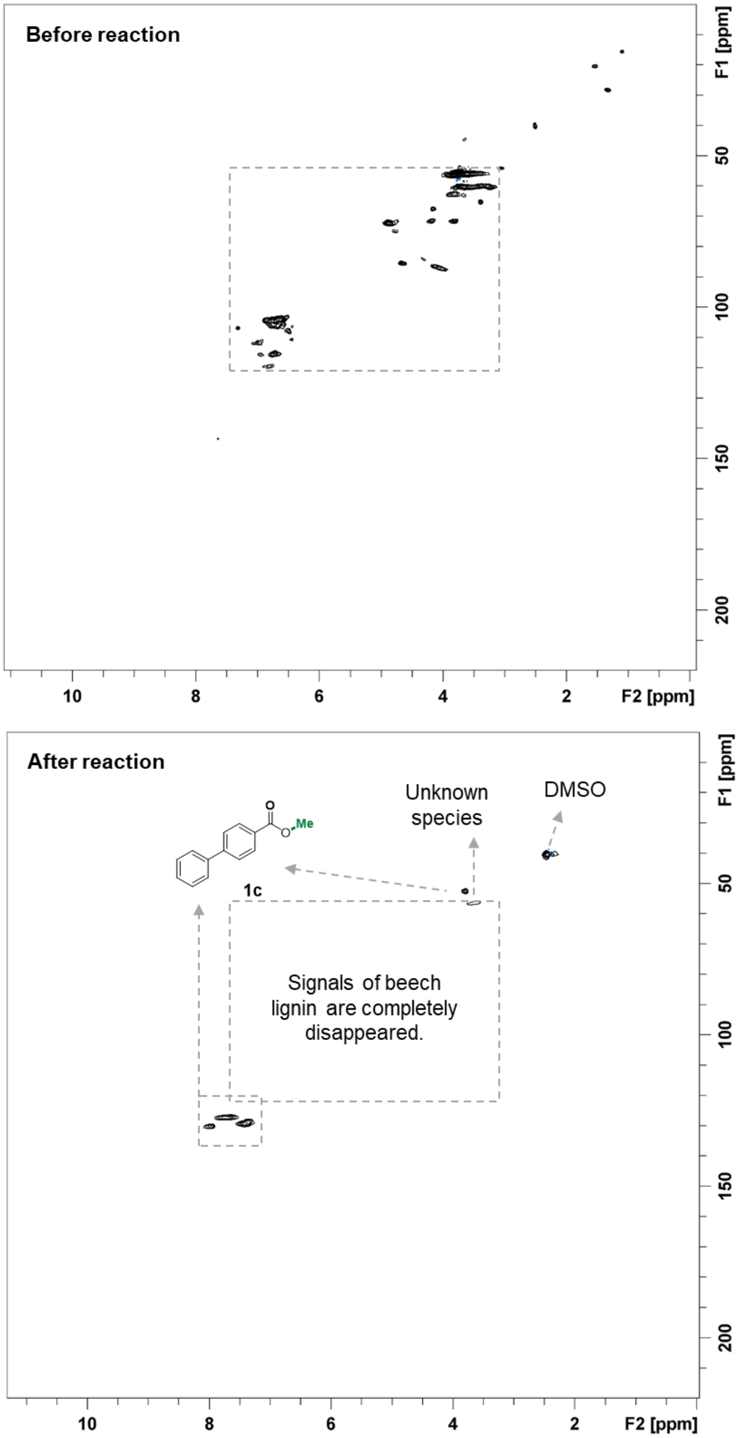

Figure S7. HSQC NMR spectra of beech lignin before reaction and the liquid phase after reaction. Reaction conditions: p-phenylbenzoic acid (0.2 mmol), beech lignin (45 mg), nano CuO (0.06 mmol), Ophen (0.02 mmol), K_2CO_3 (0.4 mmol), I_2 (0.12 mmol), DMSO-d_6 (2 mL), air balloon (1 atm), 140 °C, 10 h. The reaction mixture was flitered to remove the catalyst and undissolved base.

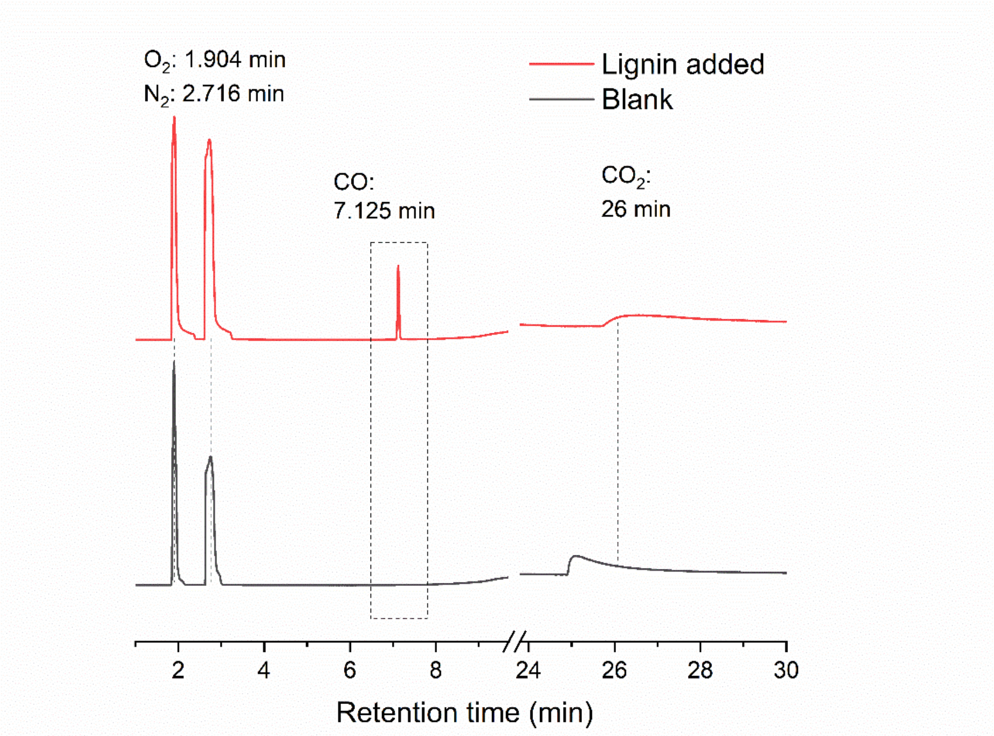

Figure S8. GC spectra of the gaseous products with and without lignin. Blank reaction: nano CuO (0.06 mmol), Ophen (0.02 mmol), K_2CO_3 (0.4 mmol), I_2 (0.12 mmol), DMSO (2 mL), air balloon (1 atm), 140 °C, 10 h.

When lignin was added into the standard reaction, CO is detected confirming that it is derived from lignin decomposition.

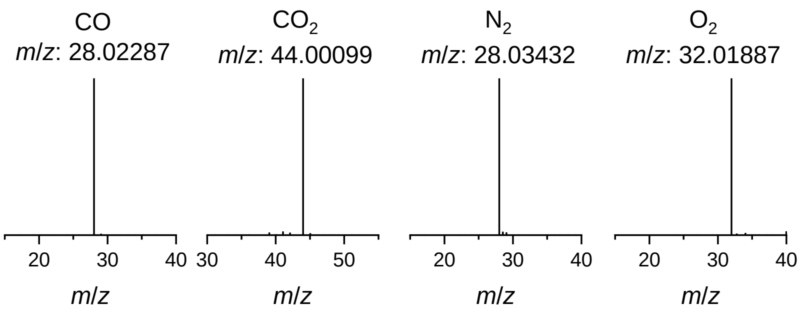

Figure S9. GC-EI-MS spectra of the gas mixture containing CO, CO_2, N_2, and O_2.

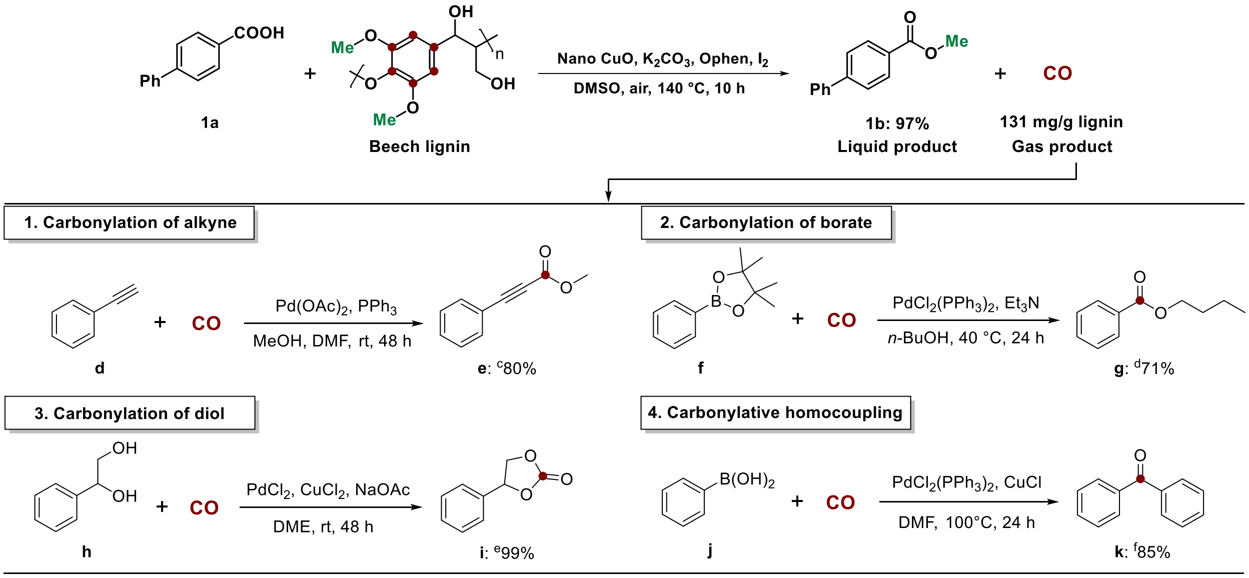

Figure S10. Reaction of the CO-containing gas product. (**1**) Phenylacetylene (0.2 mmol), $Pd(OAc)_2$ (0.02 mmol), triphenylphosphine (PPh_3, 0.04 mmol), methanol (0.4 mL), DMF (4 mL), gas mixture (1 atm), room temperature (RT), 48 h, GC yield.[8] (**2**) Pinacol phenylboronate (0.2 mmol), $PdCl_2(PPh_3)_2$ (0.01 mmol), triethylamine (Et_3N, 0.04 mmol), butanol (2 mL), gas mixture (1 atm), 40 °C, 24 h, GC yield.[9] (**3**) 1-phenylethane-2,3-diol (0.2 mmol), $PdCl_2$ (0.02 mmol), $CuCl_2$ (0.4 mmol), NaOAc (0.04 mmol), dichloromethane (DME, 5 mL), gas mixture (1 atm), RT, 48 h, GC yield.[10] (**4**) Boronic acid (0.2 mmol), $PdCl_2(PPh_3)_2$ (0.005 mmol), CuCl (0.01 mmol), DMF (2 mL), gas mixture (1 atm), 100 °C, 24 h, GC yield.[11]

The gas mixture could be directly used in carbonylation reactions without purification. Four carbonylation reactions were performed, phenylacetylene (**d**), pinacol phenylboronate (**f**), 1-phenylethane-2,3-diol (**h**), and boronic acid (**j**) were tested and successfully generated the corresponding carbonylation products, i.e. alkynoate (**e**), benzoate (**g**), cyclic carbonates (**i**), and biphenyl ketones (**k**) in high yield (71%-99%).

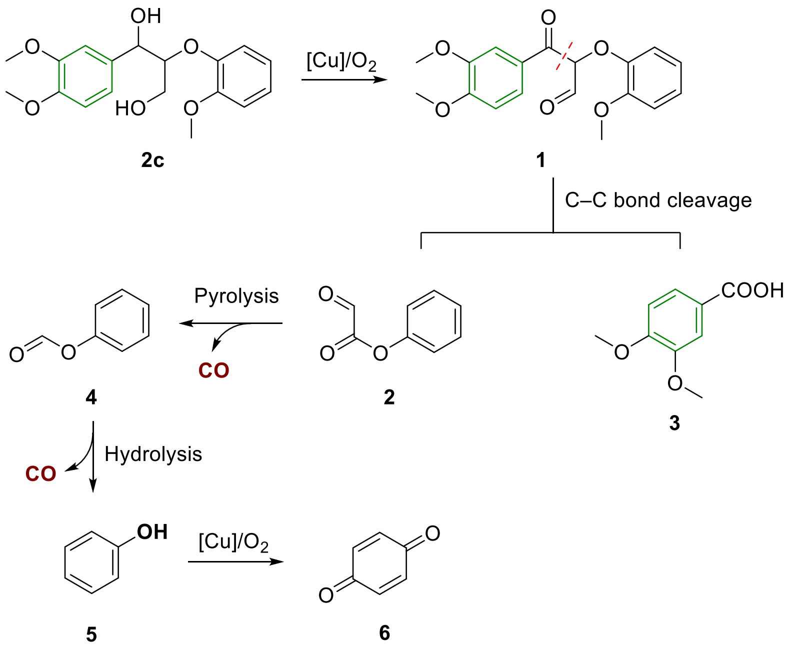

Figure S11. Decomposition pathway of the β−O−4 lignin model compound (**2c**). Decomposition of **2c** involves initial oxidation of the hydroxyl group, followed by C−C bond cleavage to give veratric acid **3** and the phenol derivative **2**. After hydrolysis of **4**, phenol **5** and its oxidative product benzoquinone **6** are formed.

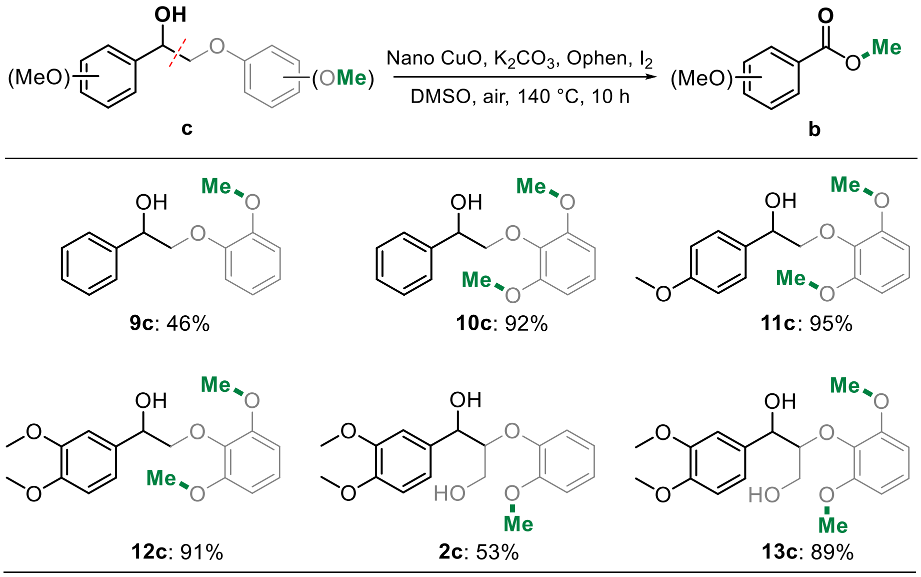

Figure S12. Fragmentation and intramolecular esterification of lignin model compounds to give methyl benzoates.

Standard reaction conditions: lignin model compound (0.2 mmol), nano CuO (0.06 mmol), Ophen (0.02 mmol), K_2CO_3 (0.4 mmol), I_2 (0.12 mmol), DMSO (2 mL), air balloon (1 atm), 140 °C, 10 h.

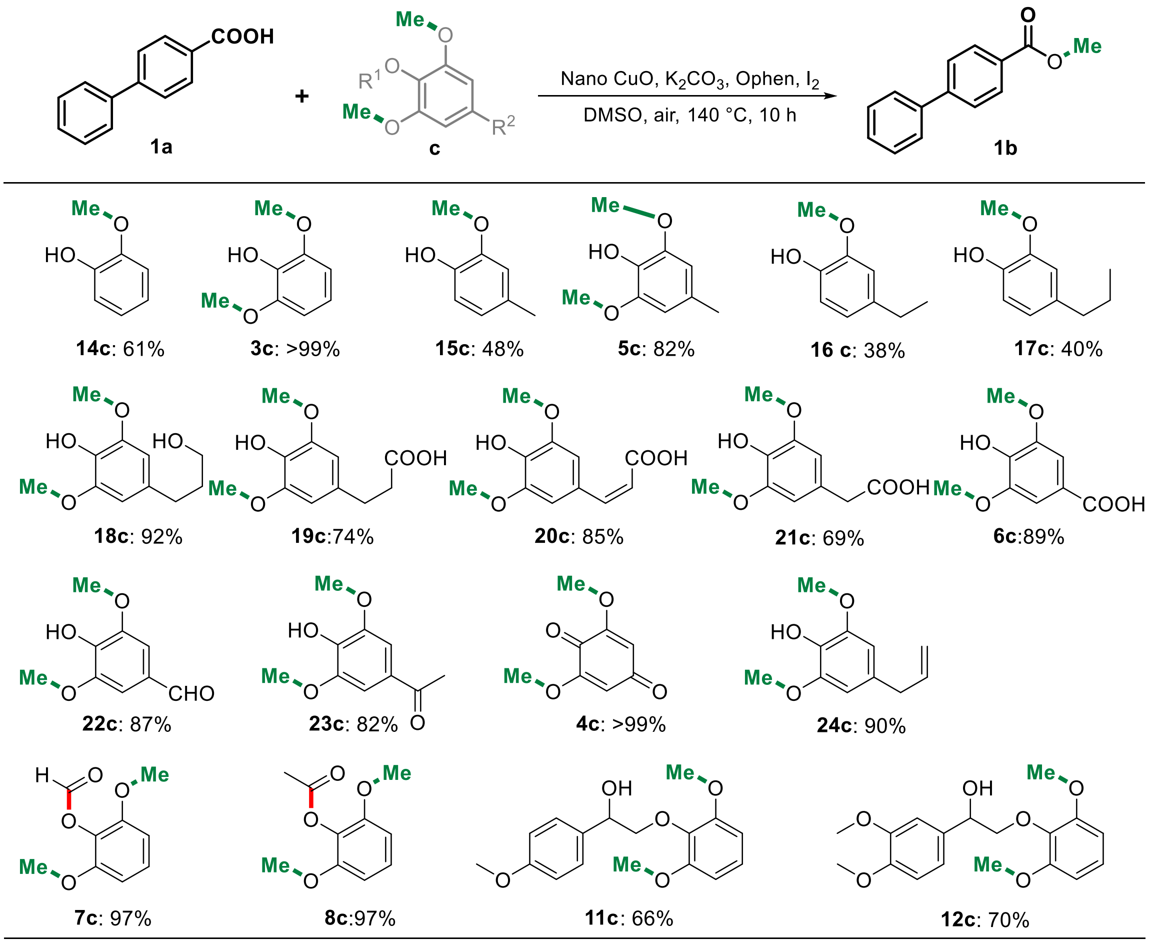

Figure S13. Cu catalyzed methylation of carboxylic acids using phenol derivatives as methylation agents.

Standard reaction conditions: *p*-phenylbenzoic acid (0.2 mmol), lignin models (0.2 mmol), nano CuO (0.06 mmol), Ophen (0.02 mmol), K_2CO_3 (0.4 mmol), I_2 (0.12 mmol), DMSO (2 mL), air balloon (1 atm), 140 °C, 10 h.

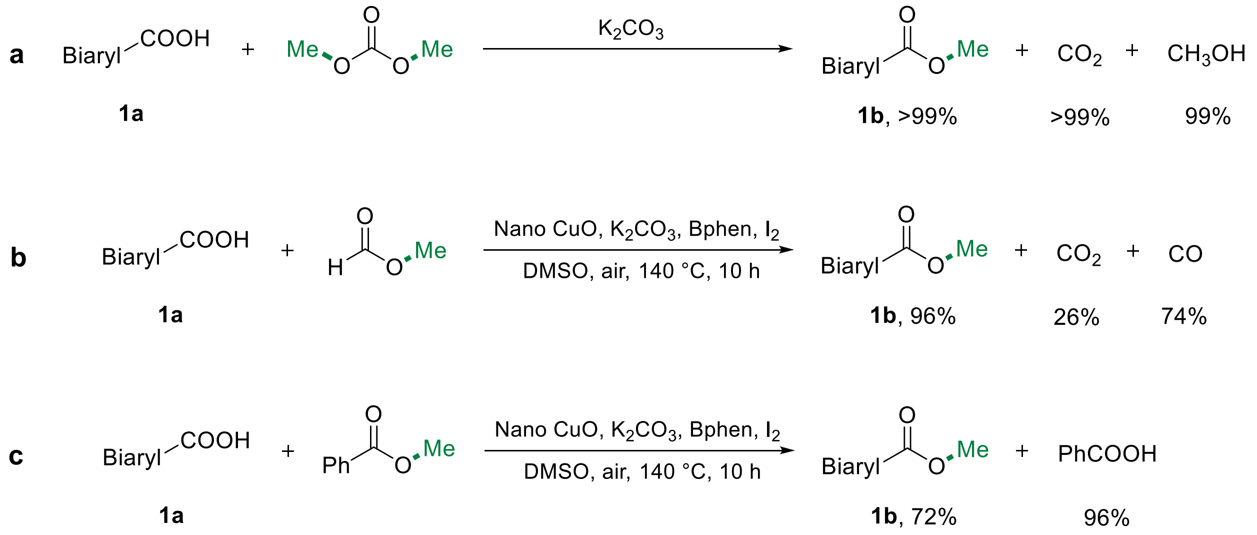

Figure S14. Reaction of *p*-phenylbenzoic acid **1a** with dimethyl carbonate (a), methyl formate (b), and methyl benzoate (c) under the standard reaction conditions.

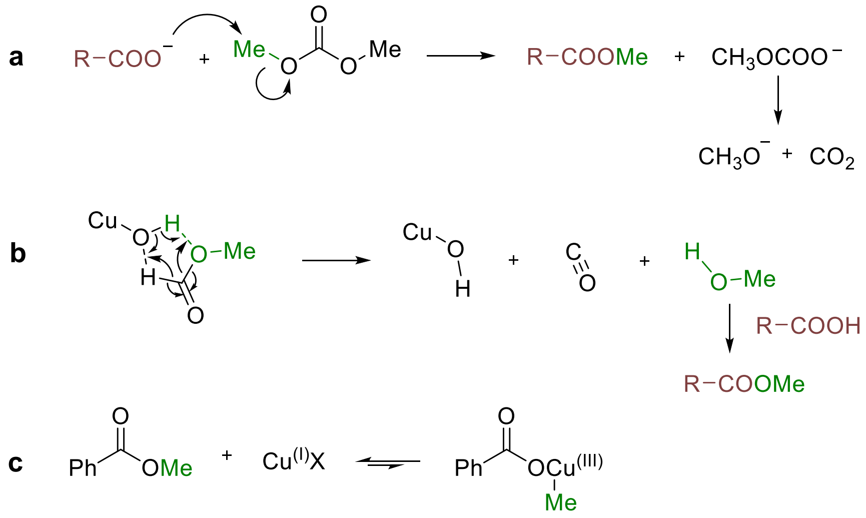

Figure S15. Mechanism of methylation of carboxylic acids with dimethyl carbonate (**a**), methyl formate (**b**), and methyl benzoate (**c**).

Pathway **a** (Fig. S15a): dimethyl carbonate is a well-known methylation agent.[12] Deprotonated acid under alkaline conditions can break the CH_3–O bond by nucleophilic attack to the methyl group and transfer to the methyl ester. Pathway **b** (Fig. S15b): as for methyl formate, Cu catalyzed rearrangement reaction under alkaline conditions can cause its decomposition to methanol.[13] The conversion of methanol and carboxylic acids to produce methyl ester will happen according to a lierature report.[14] Pathway **c** (Fig. S15c): for relatively stable methyl benzoate, the CH_3–O bond of methyl benzoate is not easily cleaved by decomposition, but can be activated via oxidative addition with a metal catalyst in a weak equilibrium.[15] The generated catalytic intermediate is highly active and can react with other intermediates that promote the following reaction.

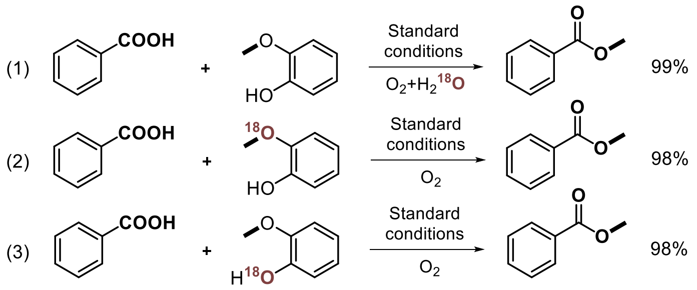

Figure S16. Isotope labeling experiments were carried out using ^{18}O labeled guaiacol, or $H_2{}^{18}O$. Abundance (%) of isotope is listed. ^{18}O labeled oxygen was highlighted in red.

Oxygen exchange between H_2O and the substrates or intermediates was not observed and addition of $H_2{}^{18}O$ did not generate ^{18}O labeled ester products (Fig. S16).

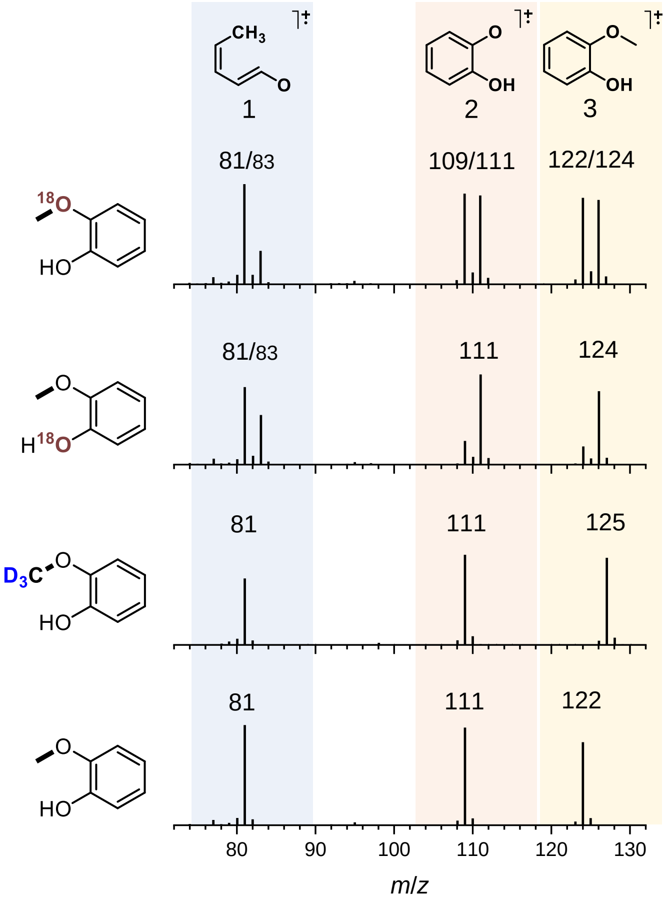

Figure S17. EI-MS of guaiacol with or without ^{2}D or ^{18}O isotope labeling.

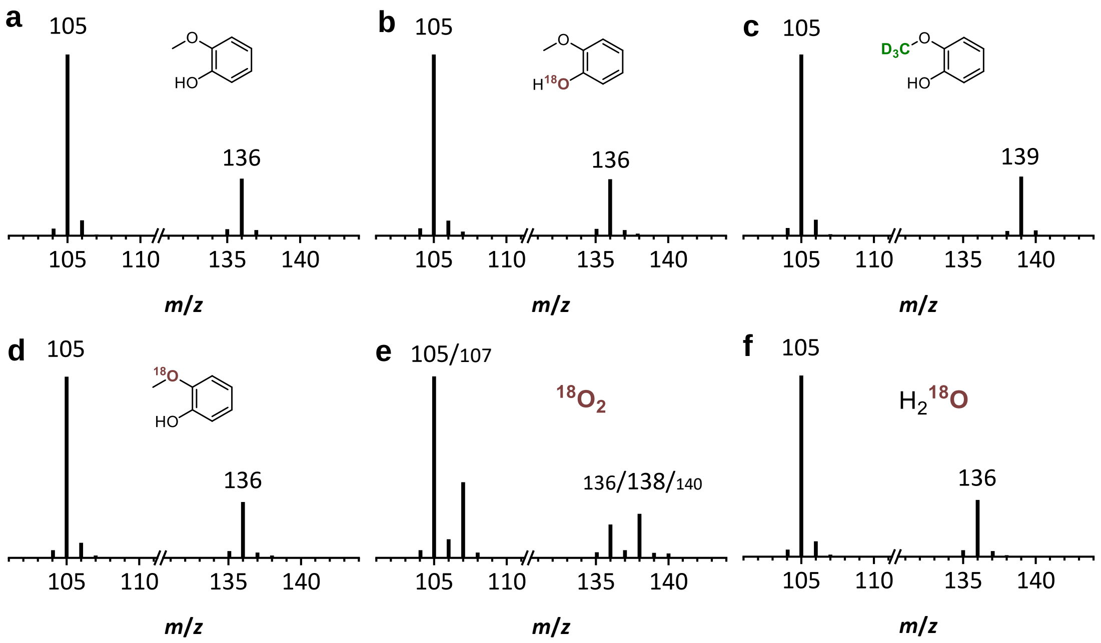

Figure S18. EI-MS of **methyl benzoate products** generated from isotope labeling experiments. (a) Non-labeled guaiacol was used. (b) Guaiacol with ^{18}O labeled on hydroxyl group was used. (c) Guaiacol with deuterated methoxy group was used. (d) Guaiacol with ^{18}O labeled on methoxy group was used. (e) 1 MPa $^{18}O_2$ was used instead of air. (f) $H_2{}^{18}O$ was added.

Standard reaction conditions: benzoic acid (0.2 mmol), guaiacol (0.4 mmol), nano CuO (0.06 mmol), Ophen (0.02 mmol), K_2CO_3 (0.4 mmol), I_2 (0.12 mmol), DMSO (2 mL), air or O_2 balloon (1 atm), 140 °C, 10 h.

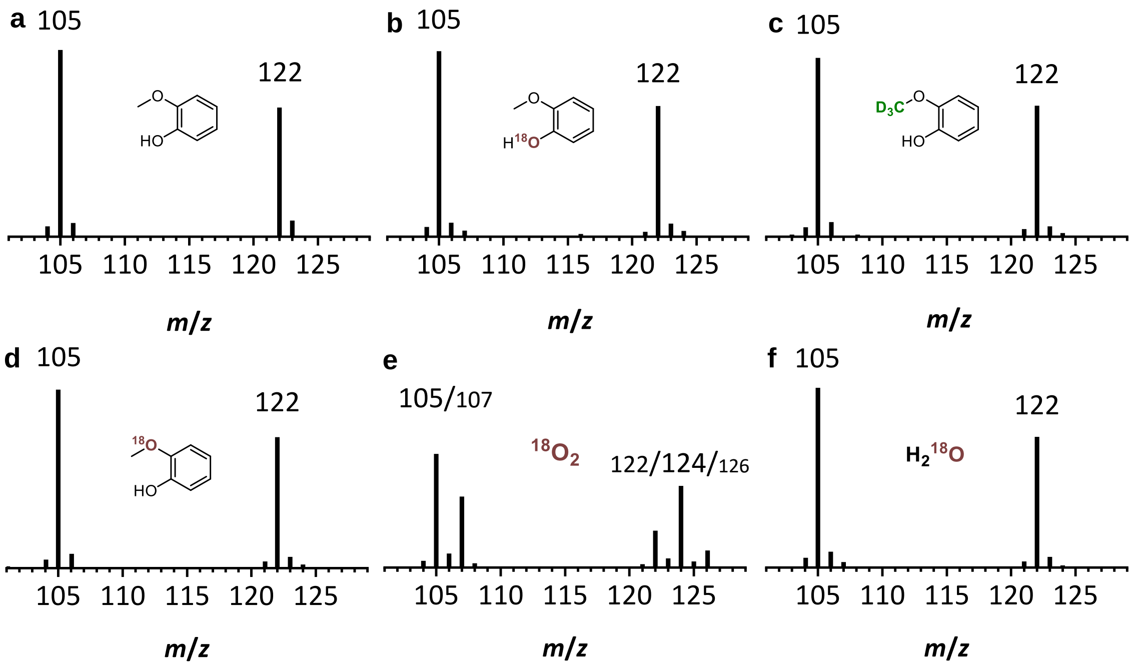

Figure S19. EI-MS of **unreacted benzoic acid substrates** generated from isotope labeling experiments. (a) Non-labeled guaiacol was used. (b) Guaiacol with ^{18}O labeled on hydroxyl group was used. (c) Guaiacol with deuterated methoxy group was used. (d) Guaiacol with ^{18}O labeled on methoxy group was used. (e) 1 MPa $^{18}O_2$ was used instead of air. (f) $H_2{}^{18}O$ was added.

Reaction conditions: benzoic acid (0.2 mmol), guaiacol (0.4 mmol), nano CuO (0.06 mmol), Ophen (0.02 mmol), K_2CO_3 (0.4 mmol), I_2 (0.12 mmol), DMSO (2 mL), air or O_2 balloon (1 atm), 140 °C, 3 h.

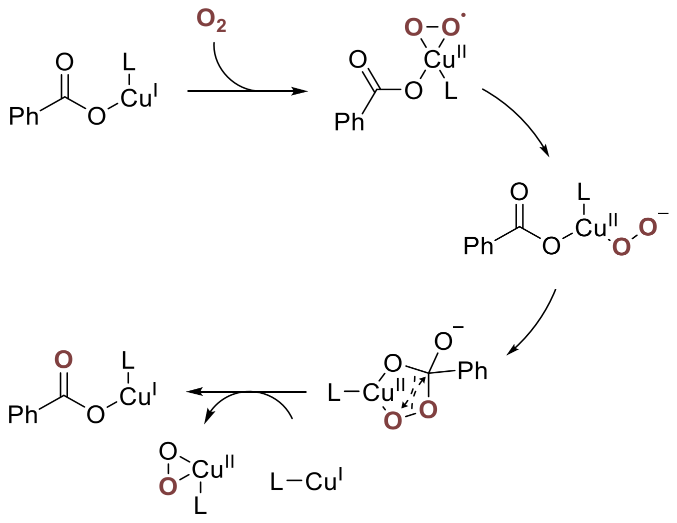

Figure S20. Plausible reaction mechanism of Cu catalyzed oxygen exchange between O_2 and carboxylic group.[16]

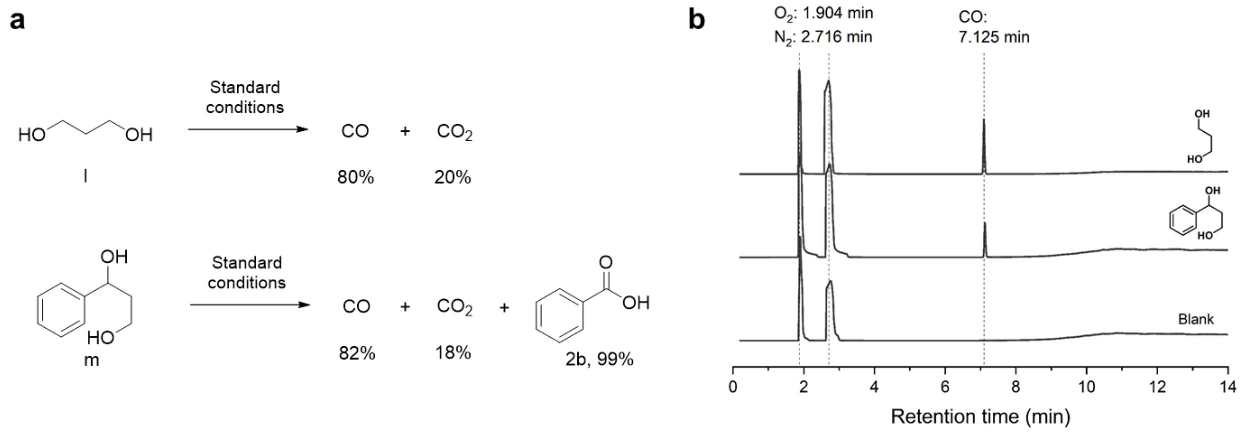

Figure S21. Decomposition of alkyl alcohols to CO. (a) Propane-1,3-diol (**l**) and 1-phenylpropane-1,3-diol (**m**) were employed as model substrates under the standard reaction conditions. Gaseous and liquid products were analyzed. Standard reaction conditions: alcohol (0.4 mmol), nano CuO (0.06 mmol), Ophen (0.02 mmol), K_2CO_3 (0.4 mmol), I_2 (0.12 mmol), DMSO (2 mL), air (10 atm), 140 °C, 10 h. (b) Original GC spectra of the gas phases along with a blank control (without alcohol added).

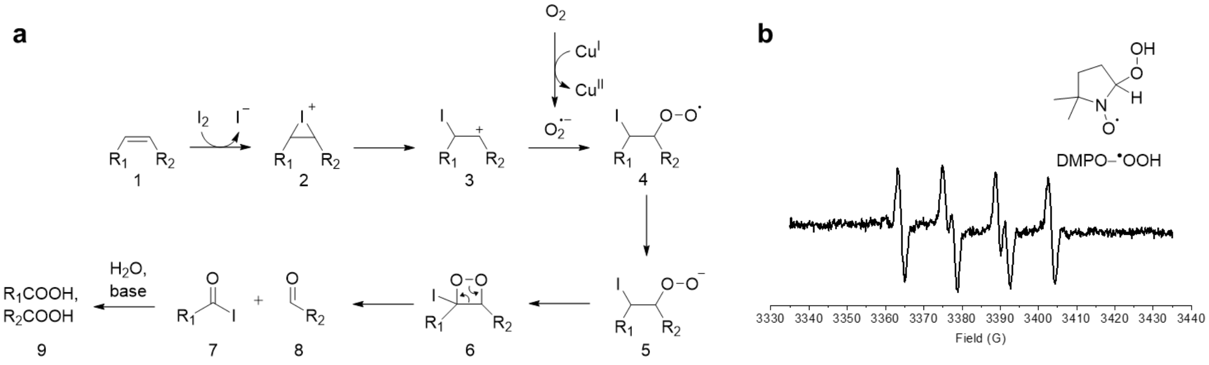

Figure S22. Tentative mechanism by which iodine promotes C=C bond cleavage of benzoquinone. (a) Proposed reaction pathway of C=C bond cleavage. (b) Electron paramagnetic resonance (EPR) spectrum of the reaction mixture (standard reaction conditions, 140 °C, 2 h). The crude reaction mixture (200 μL) was mixed with 5,5-dimethyl-1-pyrroline N-oxide (DMPO, 3 M in DMSO) and an EPR spectrum was recorded. EPR signal (g_0= 2.004) of DMPO-•OOH was identified, which demonstrates the formation of superoxide radical during the reaction of O_2 and Cu catalyst.[17]

The activation of C=C bonds is required for the decomposition of benzoquinone. It has been reported that heterolysis of I_2 affords I^+ and I^- (or I_3^-).[18] I^+ reacts with the C=C bond to form a three-membered iodonium ion containing ring **2**.[19] This process activates C=C bond to afford the carbon-centered cation **3**.[19a] Superoxide radical generated by the oxidation of Cu^I catalyst react with **3** to give superoxide radical intermediate **4**.[20] After reduction of **4** to peroxide anion **5**, intramolecular nucleophilic attack form dioxetane intermediate **6**. Fragmentation of dioxetane **6** lead to the C−C bond cleavage to give **7** and **8**.[21] After hydrolysis or oxidation, carboxylic acid products **9** are obtained.[22]

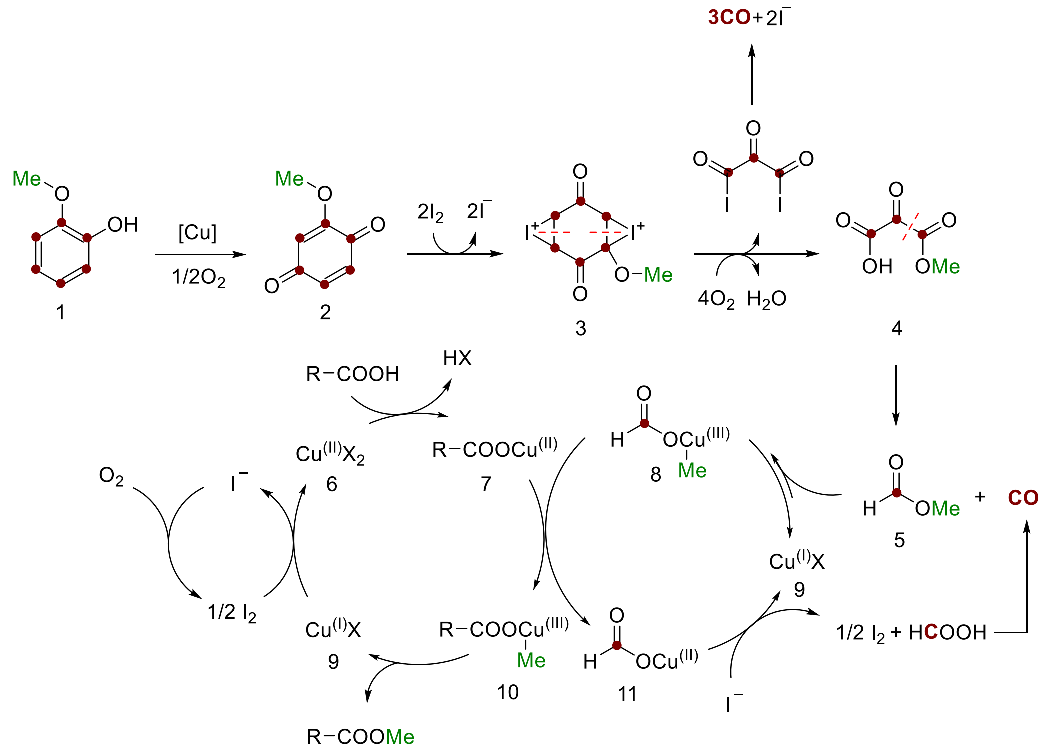

Figure S23. Proposed reaction pathway from phenol to methyl ester and CO products.

The reaction pathway for the depolymerization of lignin to phenols was described in Fig. S11. This figure shows the mechanism from the oxidative fragmentation of phenols. The phenyl carbon and methoxy carbon) atoms were both labelled, and both could be upgraded to CO and methyl ester, respectively. Methoxy substituted phenol **1** decomposes to methyl formate **5** and CO mediated by benzoquinone **2**,[23] followed by fragmentation to polycarbonyl compound **4** catalyzed by iodine catalyst.[14, 19a] Thermal decomposition of **4** gives methyl formate **5** and CO. The following step is similar as the Chan−Lam type cross coupling reaction for alkylation of heteroatom nucleophiles such as carboxylic acids and amines catalyzed by Cu Catalysts.[24] Organic Cu^{III} species **8** may be formed by the oxidative addition of **5** to the Cu^{I} catalyst **9**. Carboxylic acid is activated by the Cu^{II} catalyst **6**. Transmetallation between the Cu^{III} intermediate **8** and carboxylate Cu^{II} **7** generates intermediate **10**, followed by reductive elimination to produce methyl ester products. Iodine promotes the redox reaction between the Cu catalyst and O_2 and enables recycling of Cu catalyst during the reaction.[14, 25]

NMR spectra

Isotope labeling compounds:

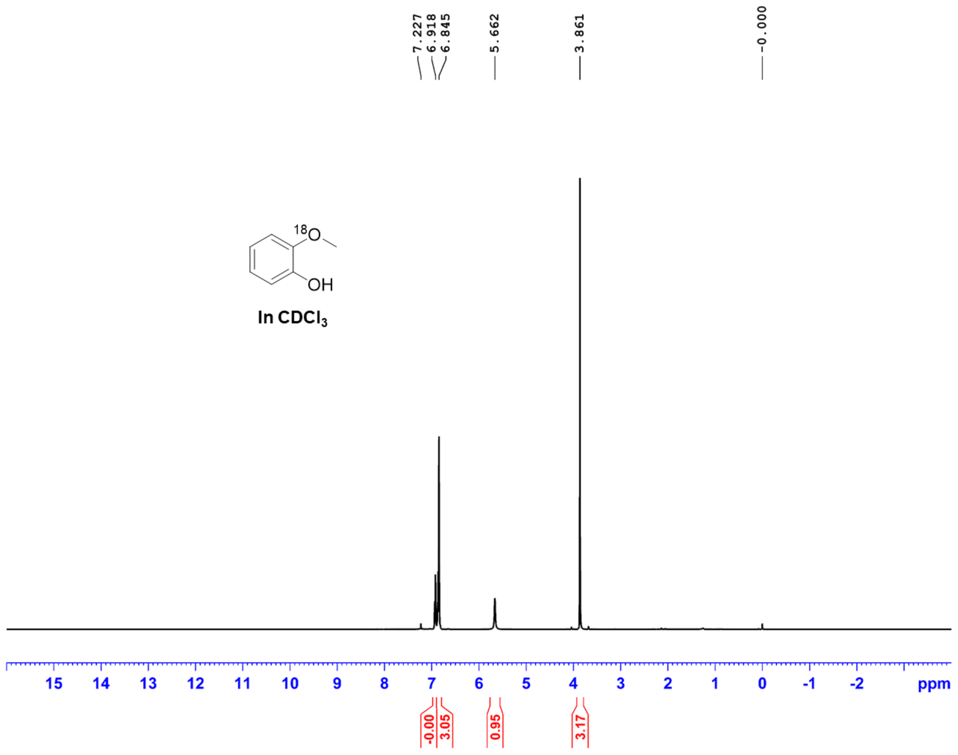

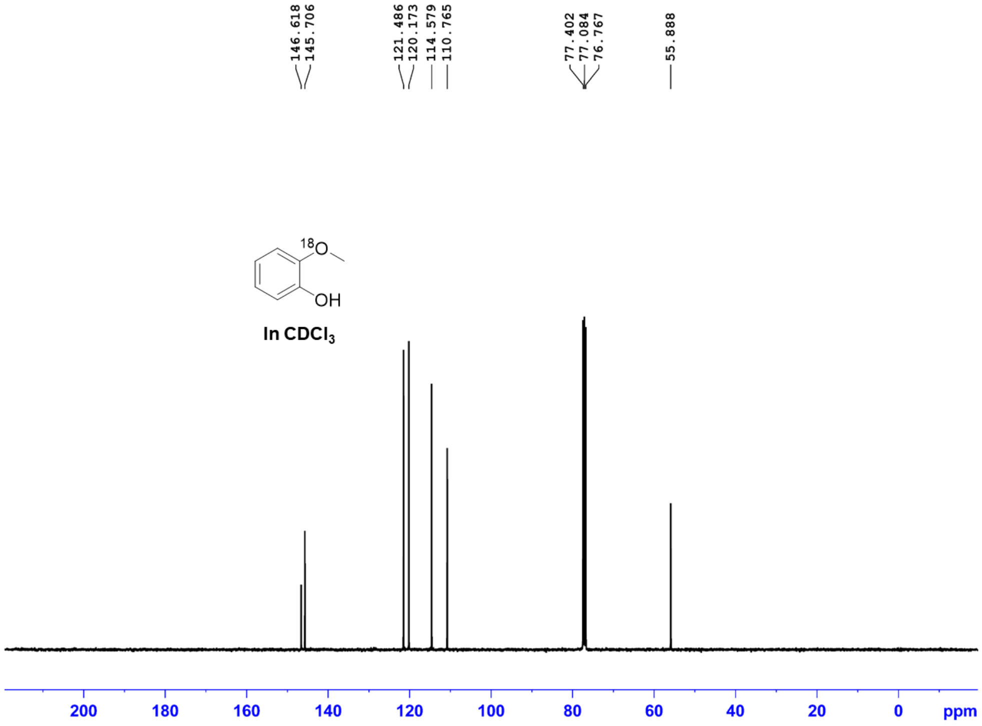

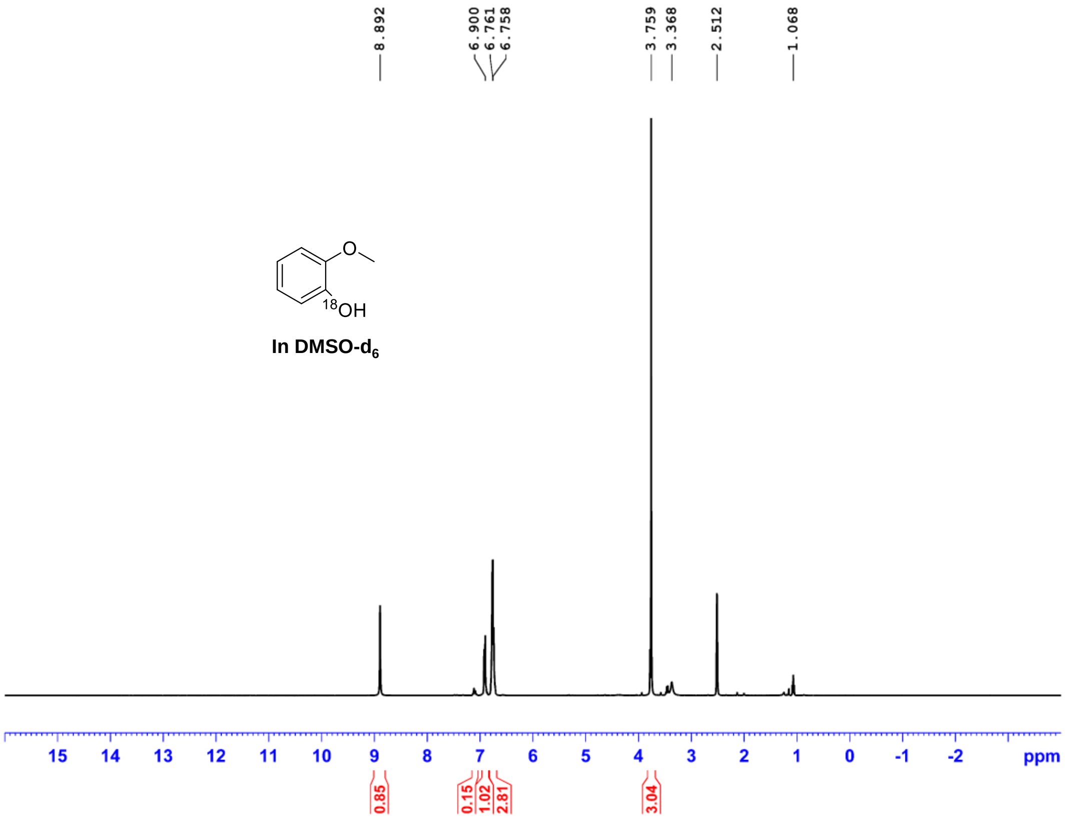
8.892
6.900
6.761
6.758
3.759
3.368
2.512
1.068
O
18OH
In DMSO-d6
15
14
13
12
11
10
9
8
7
6
5
4
3
2
1
0
-1
-2
ppm
0.85
0.15
1.02
2.81
3.04

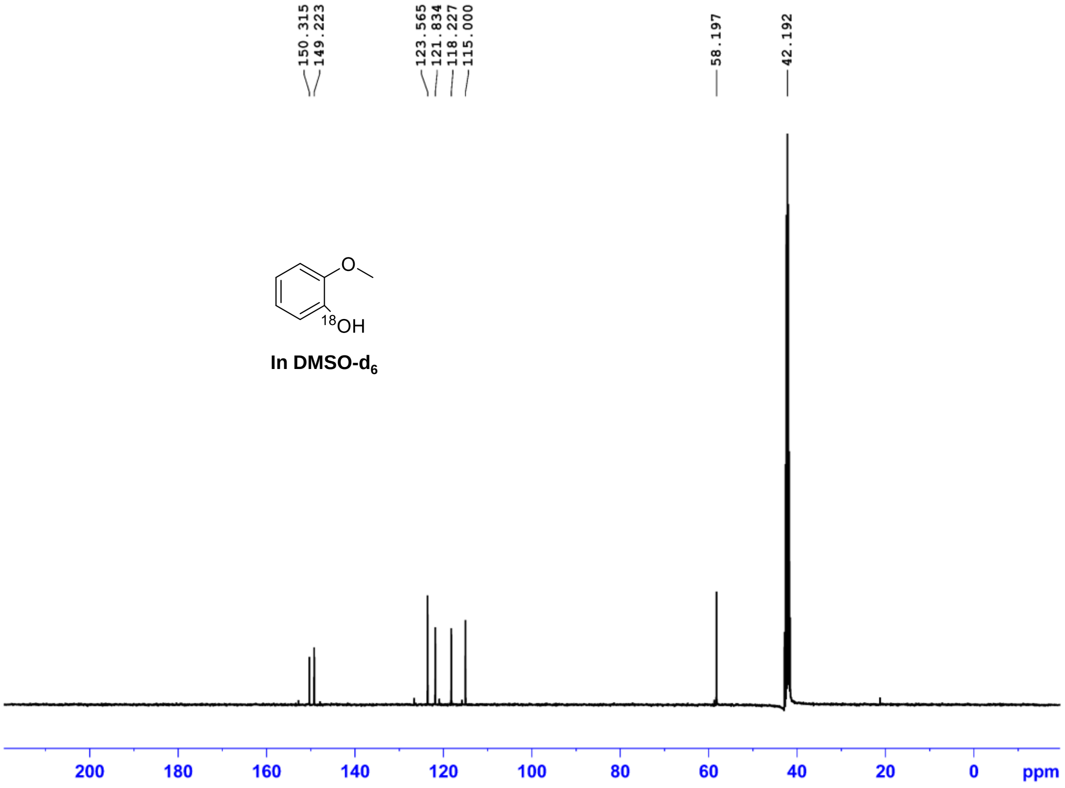
150.315
149.223
123.565
121.834
118.227
115.000
58.197
42.192
O
18OH
In DMSO-d6
200
180
160
140
120
100
80
60
40
20
0
ppm

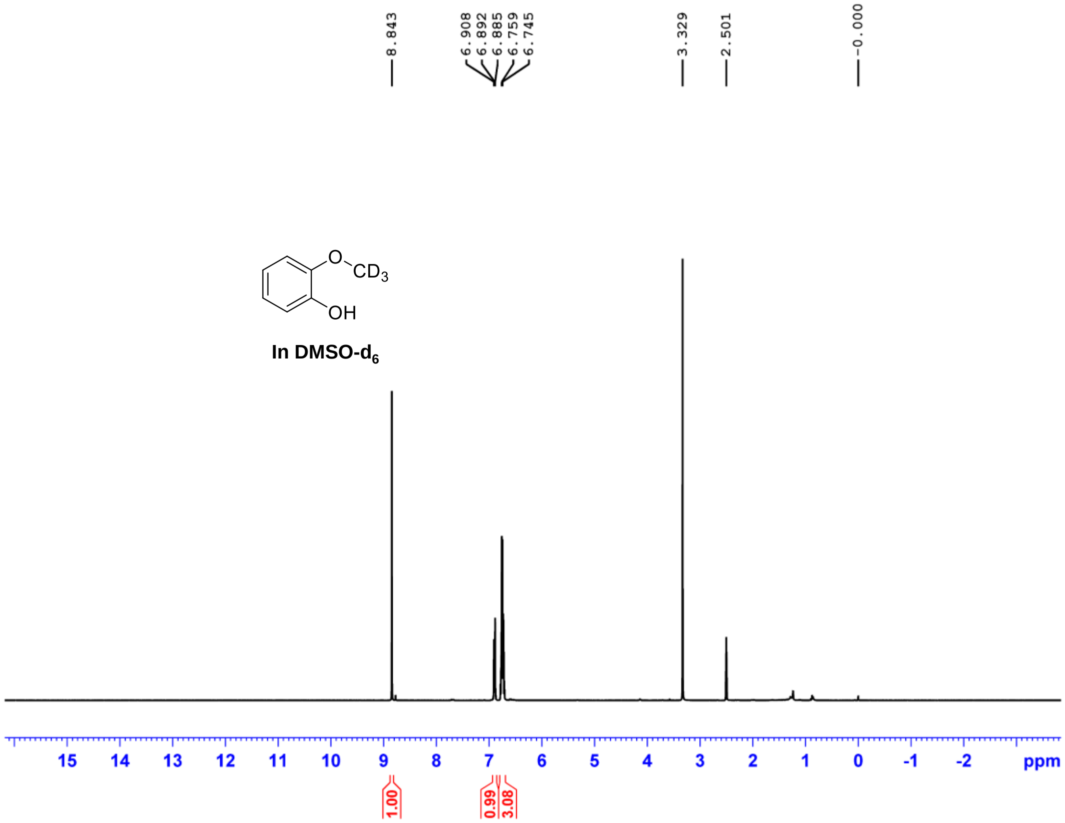
8.843
6.908
6.892
6.885
6.759
6.745
3.329
2.501
-0.000
O
CD3
OH
In DMSO-d6
15 14 13 12 11 10 9 8 7 6 5 4 3 2 1 0 -1 -2 ppm
1.00
0.99
3.08

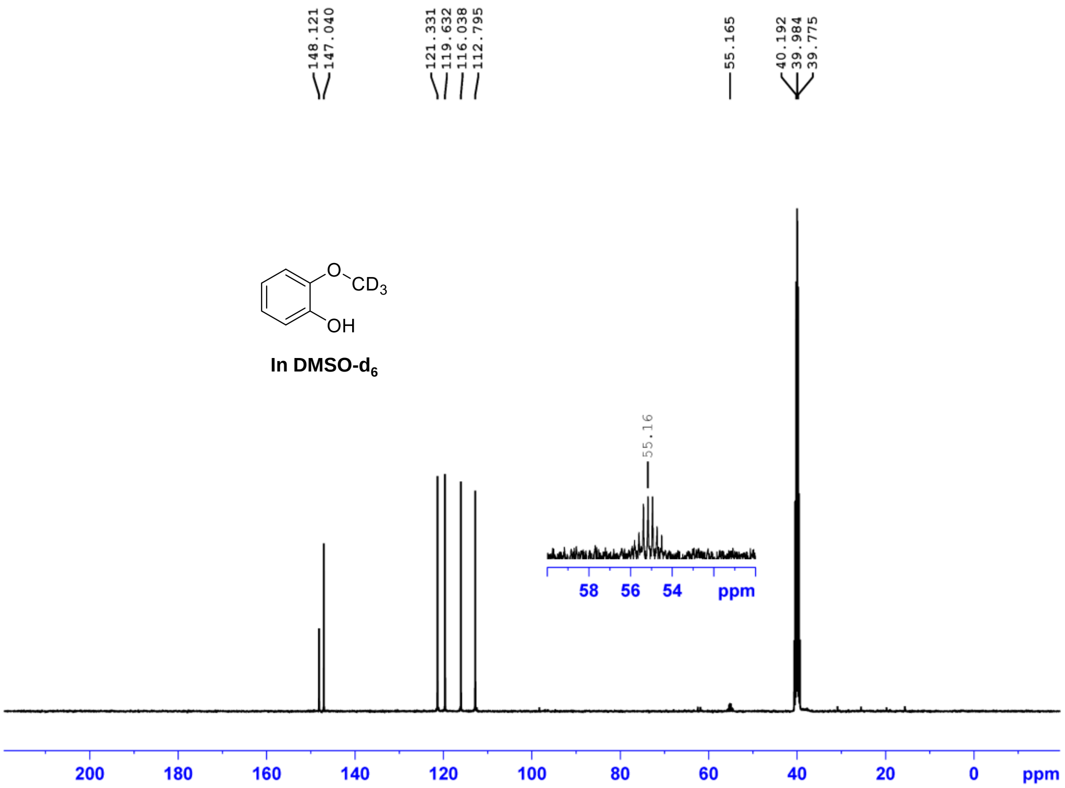
148.121
147.040
121.331
119.632
116.038
112.795
55.165
40.192
39.984
39.775
O
CD3
OH
In DMSO-d6
55.16
58 56 54 ppm
200 180 160 140 120 100 80 60 40 20 0 ppm

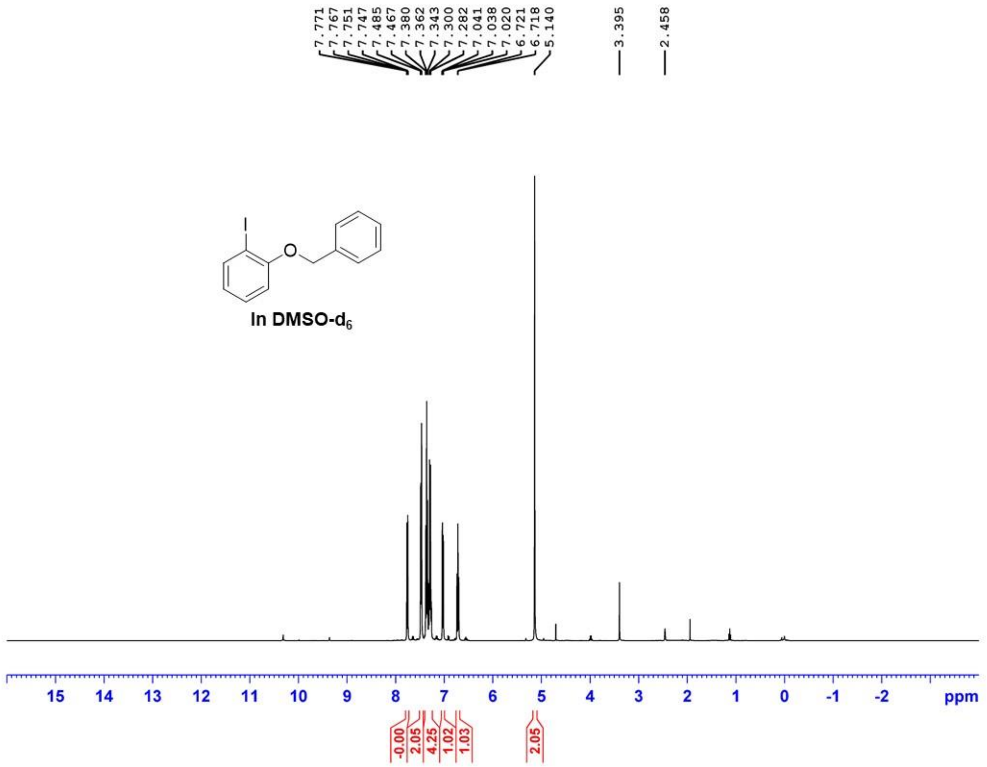
7.771
7.767
7.751
7.747
7.485
7.467
7.380
7.362
7.343
7.300
7.282
7.041
7.038
7.020
6.721
6.718
5.140
3.395
2.458
I
O
In DMSO-d6
15 14 13 12 11 10 9 8 7 6 5 4 3 2 1 0 -1 -2 ppm
-0.00
2.05
4.25
1.02
1.03
2.05

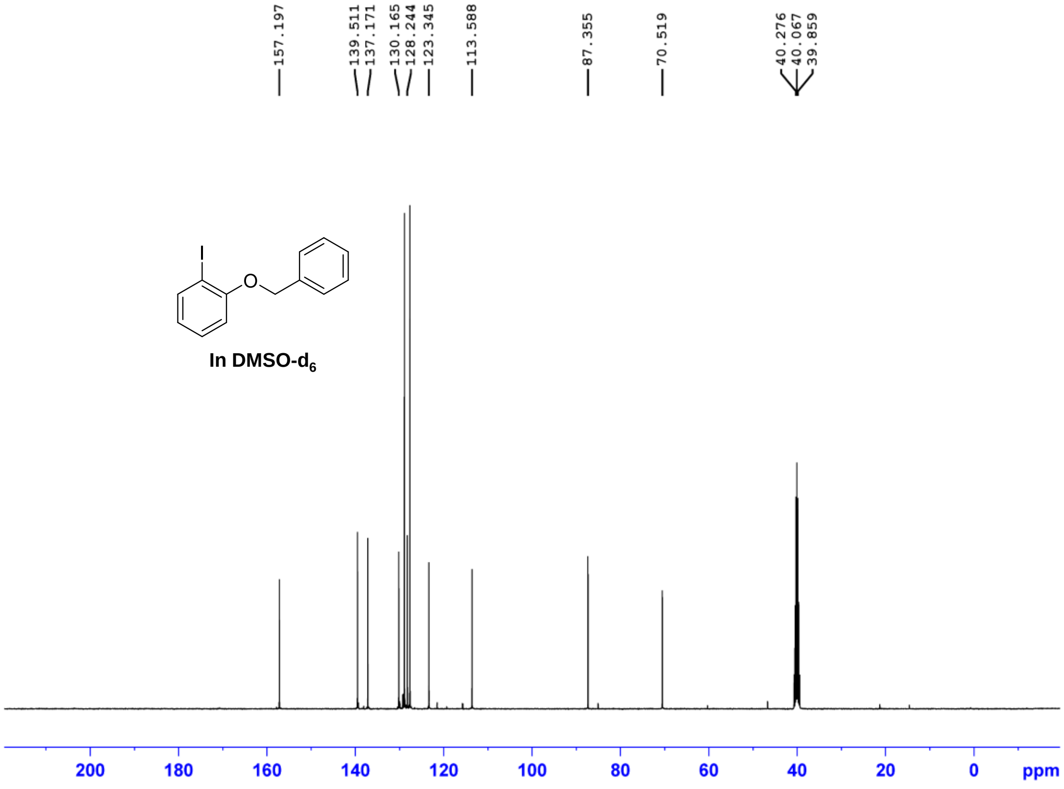
157.197
139.511
137.171
130.165
128.244
123.345
113.588
87.355
70.519
40.276
40.067
39.859
I
O
In DMSO-d6
200 180 160 140 120 100 80 60 40 20 0 ppm

Lignin model compounds:

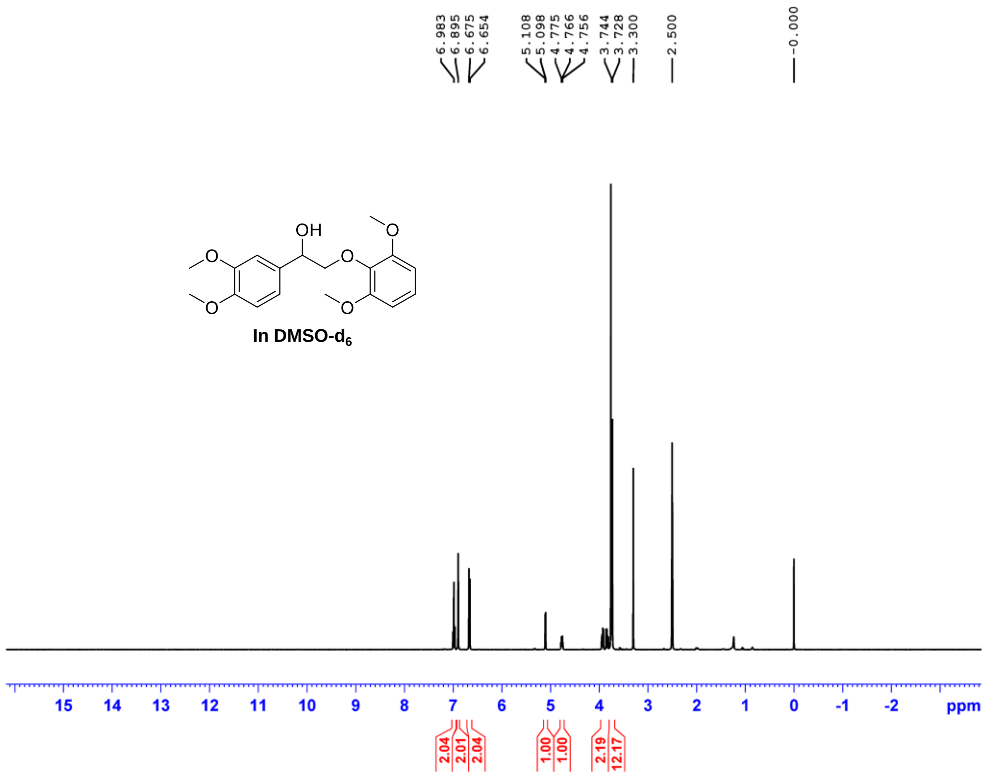

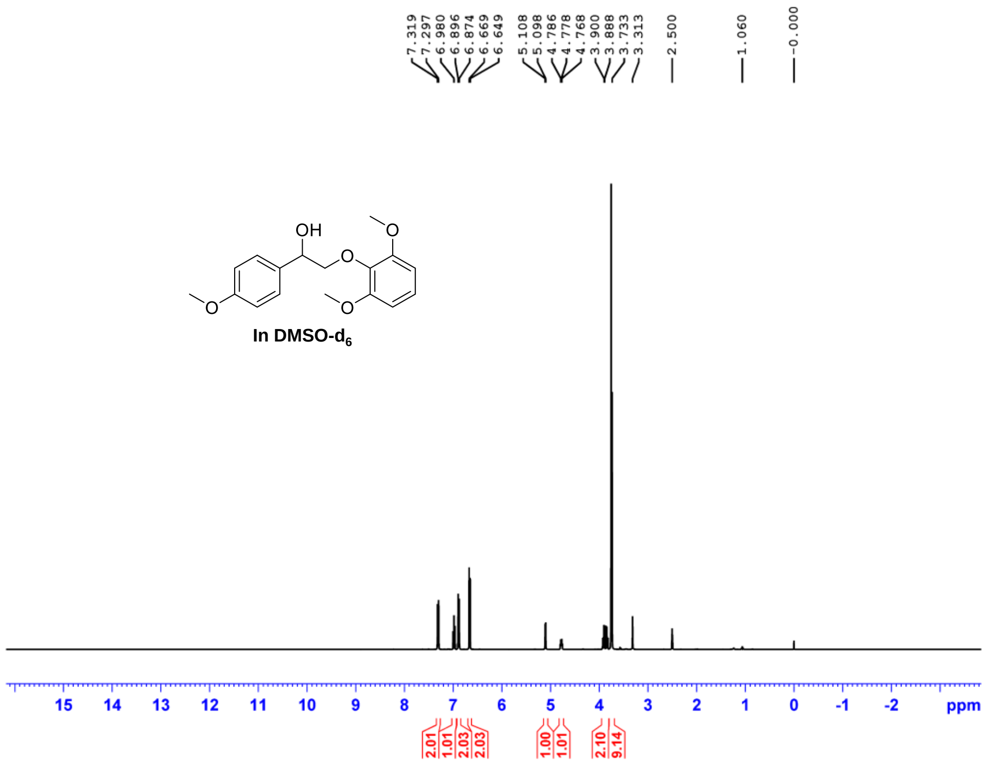

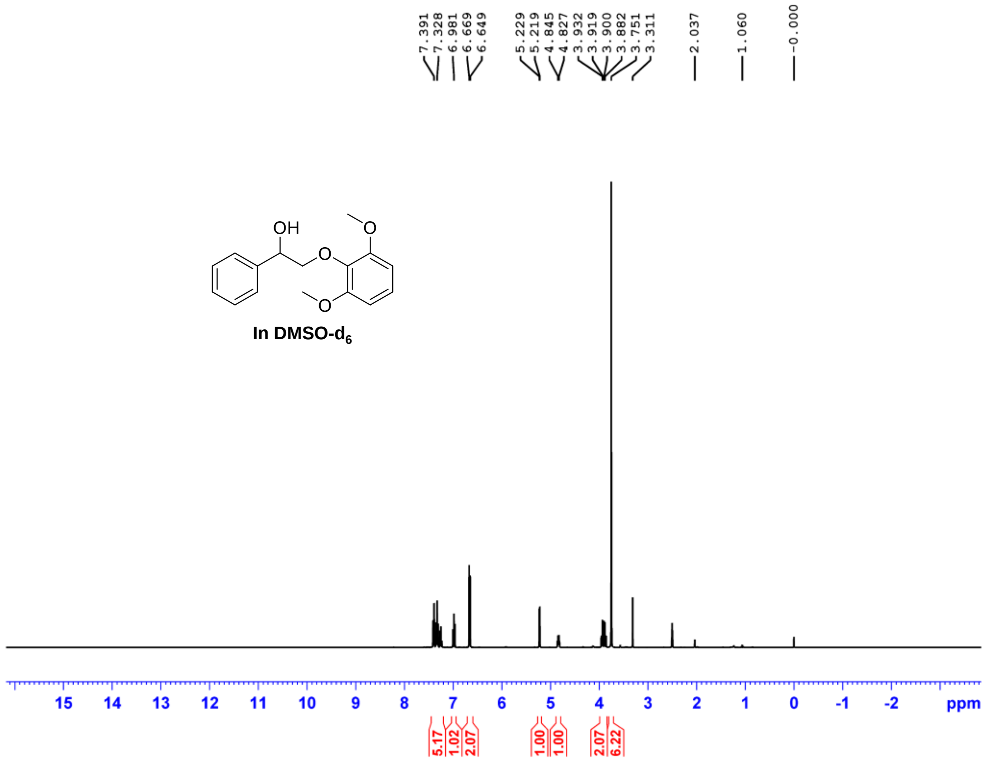
7.391
7.328
6.981
6.669
6.649
5.229
5.219
4.845
4.827
3.932
3.919
3.900
3.882
3.751
3.311
2.037
1.060
-0.000
OH
O
O
O
In DMSO-d6
15 14 13 12 11 10 9 8 7 6 5 4 3 2 1 0 -1 -2 ppm
5.17
1.02
2.07
1.00
1.00
2.07
6.22

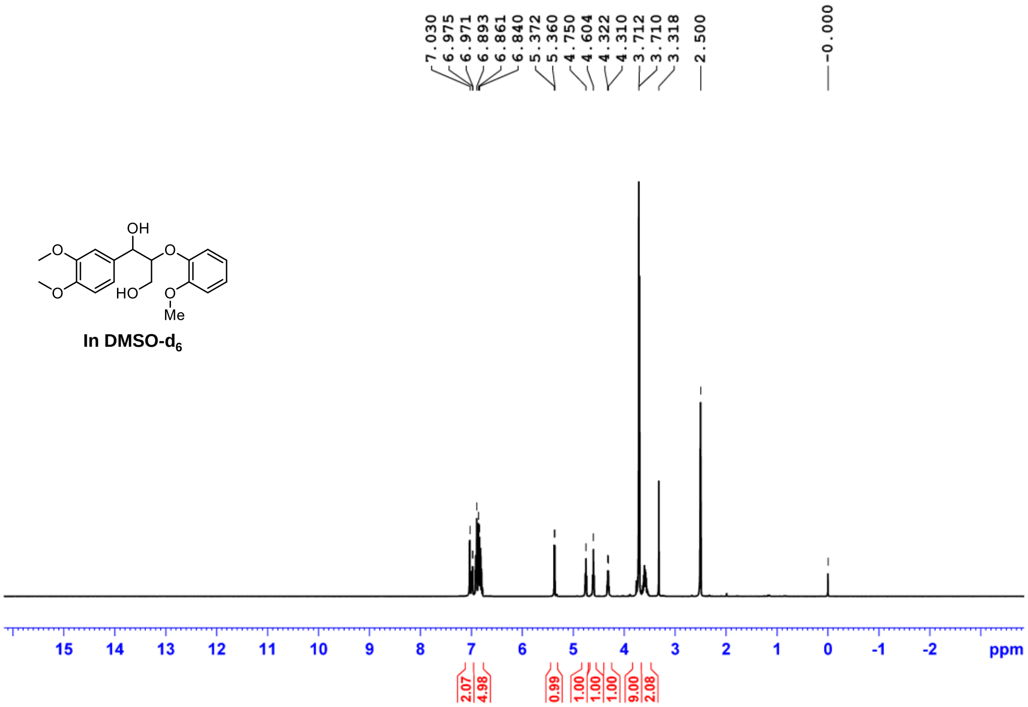
7.030
6.975
6.971
6.893
6.861
6.840
5.372
5.360
4.750
4.604
4.322
4.310
3.712
3.710
3.318
2.500
-0.000
OH
O
O
O
HO
O
Me
In DMSO-d6
15 14 13 12 11 10 9 8 7 6 5 4 3 2 1 0 -1 -2 ppm
2.07
4.98
0.99
1.00
1.00
1.00
9.00
2.08

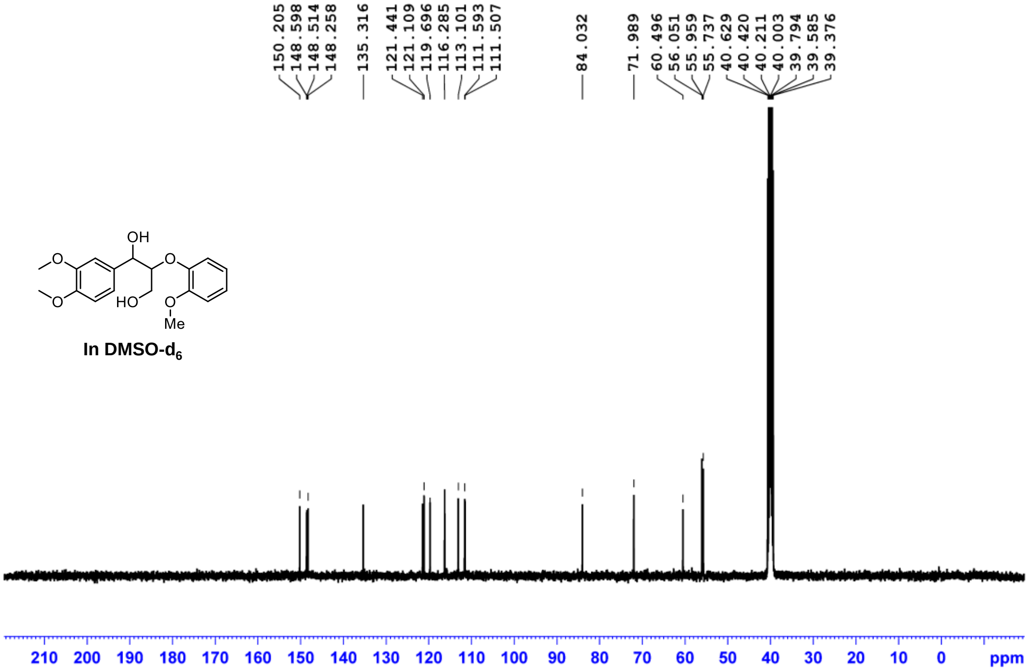

150.205
148.598
148.514
148.258
135.316
121.441
121.109
119.696
116.285
113.101
111.593
111.507
84.032
71.989
60.496
56.051
55.959
55.737
40.629
40.420
40.211
40.003
39.794
39.585
39.376
OH
O
O
O
HO
O
Me
In DMSO-d6
210 200 190 180 170 160 150 140 130 120 110 100 90 80 70 60 50 40 30 20 10 0 ppm

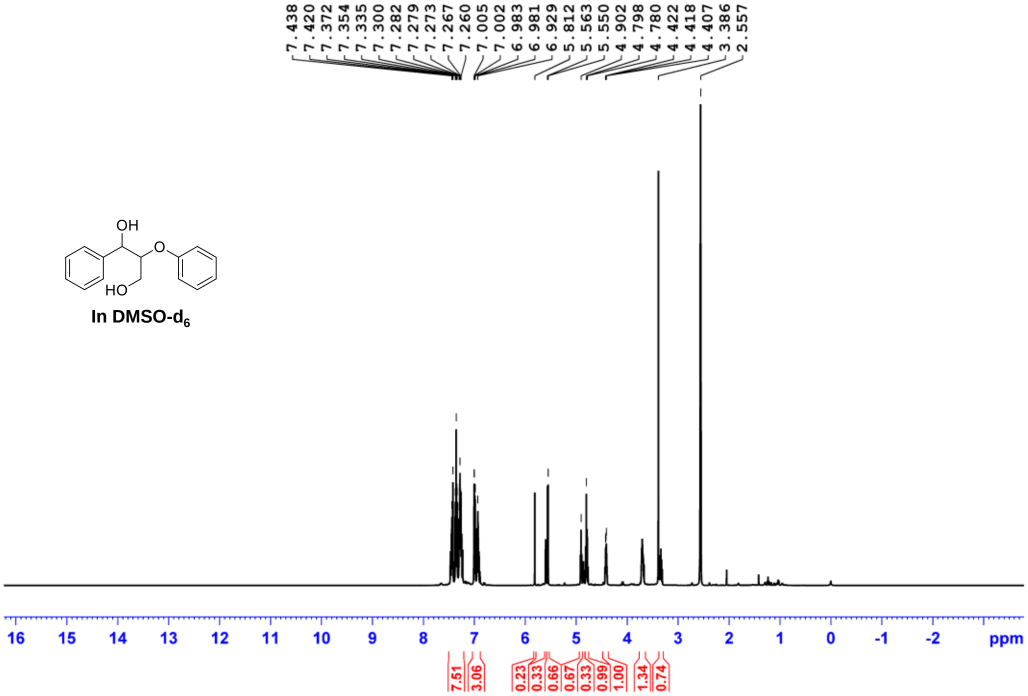

7.438
7.420
7.372
7.354
7.335
7.300
7.282
7.279
7.273
7.267
7.260
7.005
7.002
6.983
6.981
6.929
5.812
5.563
5.550
4.902
4.798
4.780
4.422
4.418
4.407
3.386
2.557
OH
O
HO
In DMSO-d6
16 15 14 13 12 11 10 9 8 7 6 5 4 3 2 1 0 -1 -2 ppm
7.51
3.06
0.23
0.33
0.66
0.67
0.33
0.99
1.00
1.34
0.74

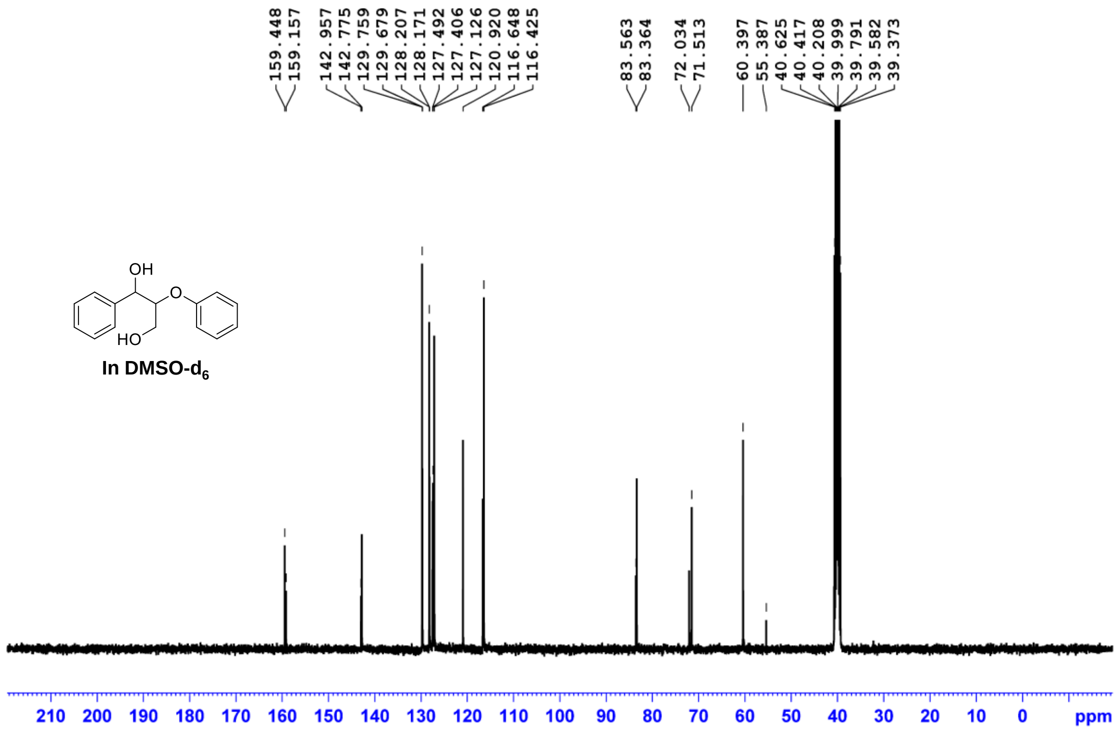
159.448
159.157
142.957
142.775
129.759
129.679
128.207
128.171
127.492
127.406
127.126
120.920
116.648
116.425
83.563
83.364
72.034
71.513
60.397
55.387
40.625
40.417
40.208
39.999
39.791
39.582
39.373
OH
O
HO
In DMSO-d6
210 200 190 180 170 160 150 140 130 120 110 100 90 80 70 60 50 40 30 20 10 0 ppm

Ester products:

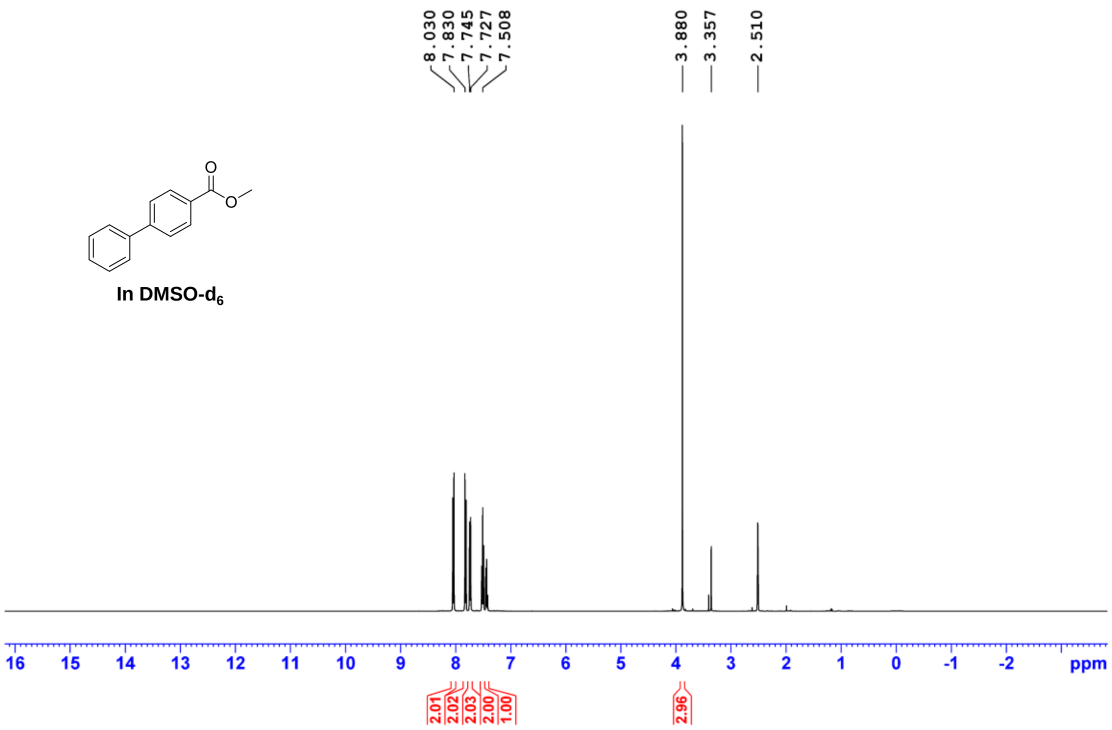

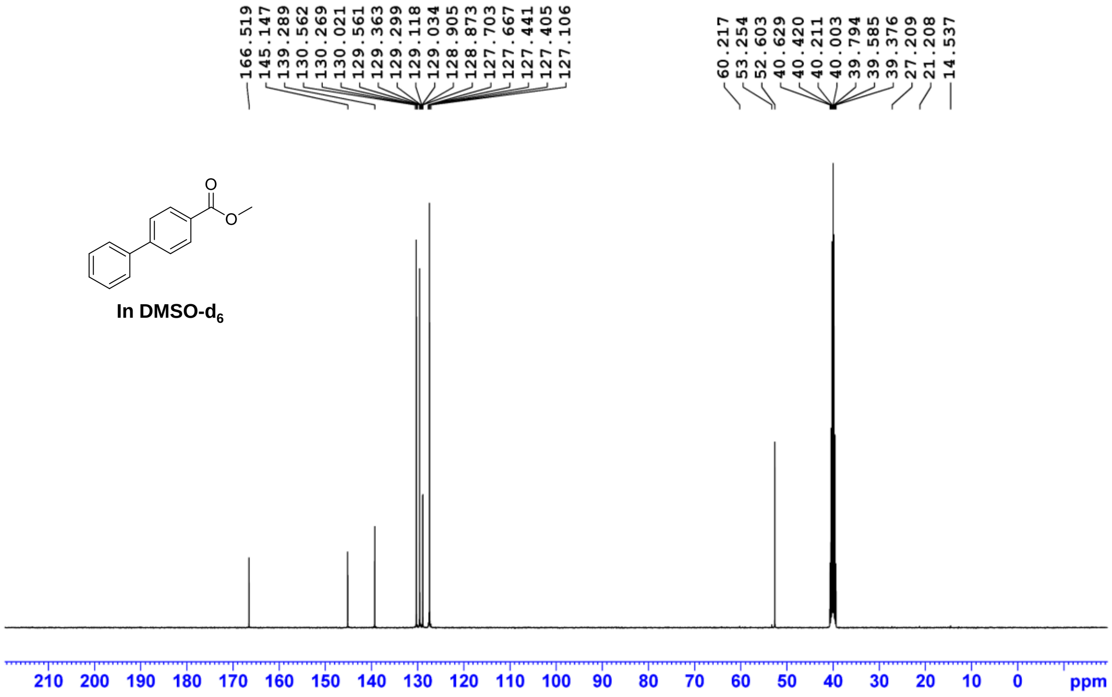

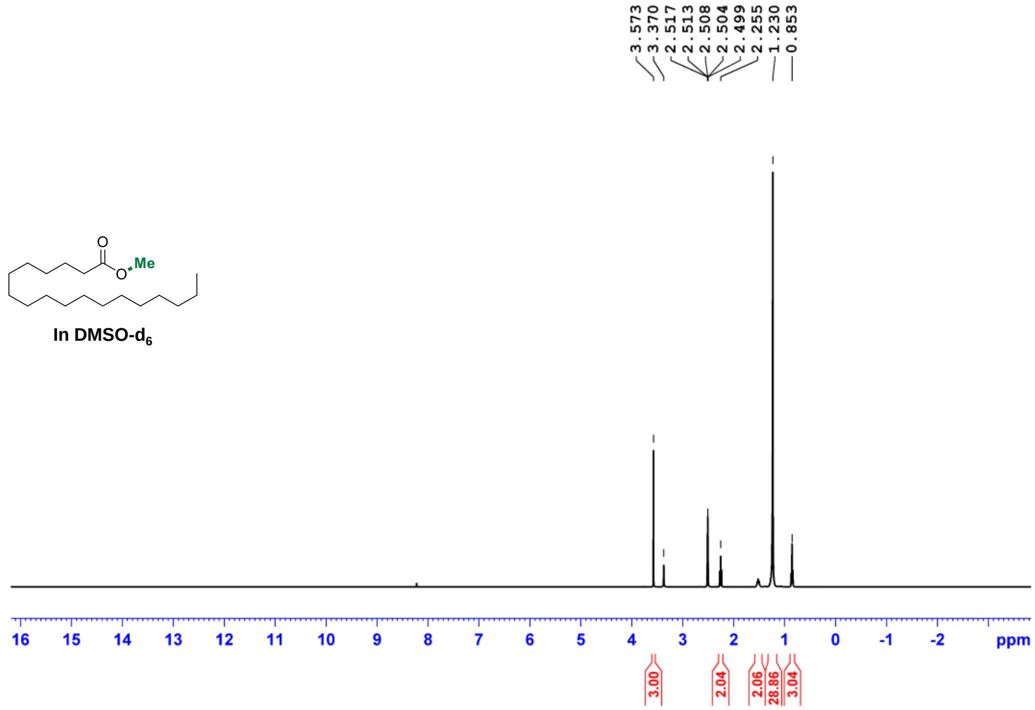
3.573
3.370
2.517
2.513
2.508
2.504
2.499
2.255
1.230
0.853
O
O
Me
In DMSO-d6
16 15 14 13 12 11 10 9 8 7 6 5 4 3 2 1 0 -1 -2 ppm
3.00
2.04
2.06
28.86
3.04

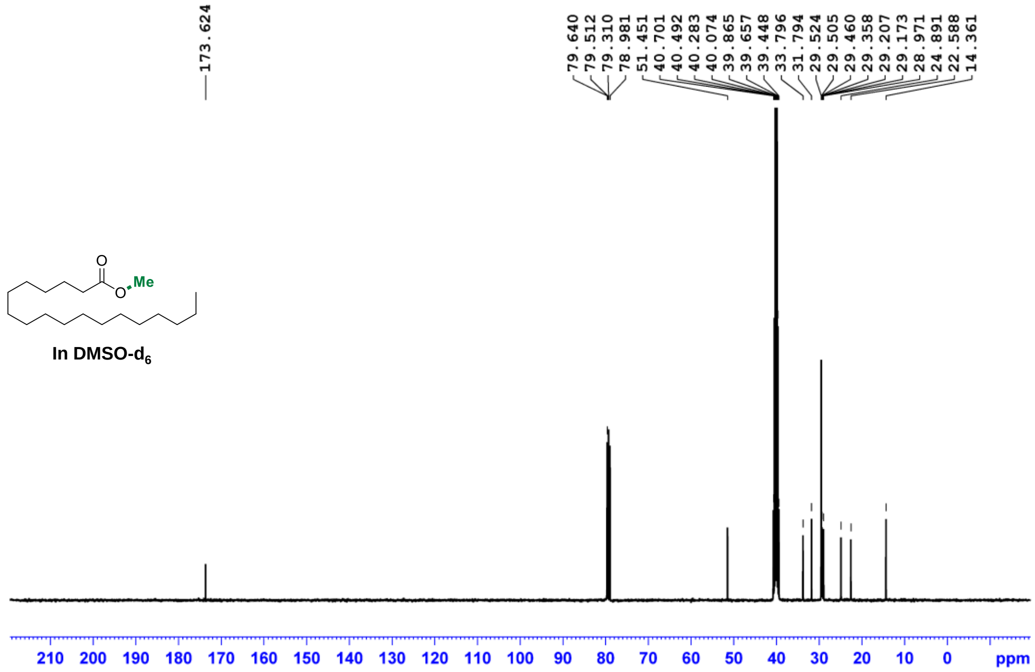
173.624
79.640
79.512
79.310
78.981
51.451
40.701
40.492
40.283
40.074
39.865
39.657
39.448
33.796
31.794
29.524
29.505
29.460
29.358
29.207
29.173
28.971
24.891
22.588
14.361
O
O
Me
In DMSO-d6
210 200 190 180 170 160 150 140 130 120 110 100 90 80 70 60 50 40 30 20 10 0 ppm

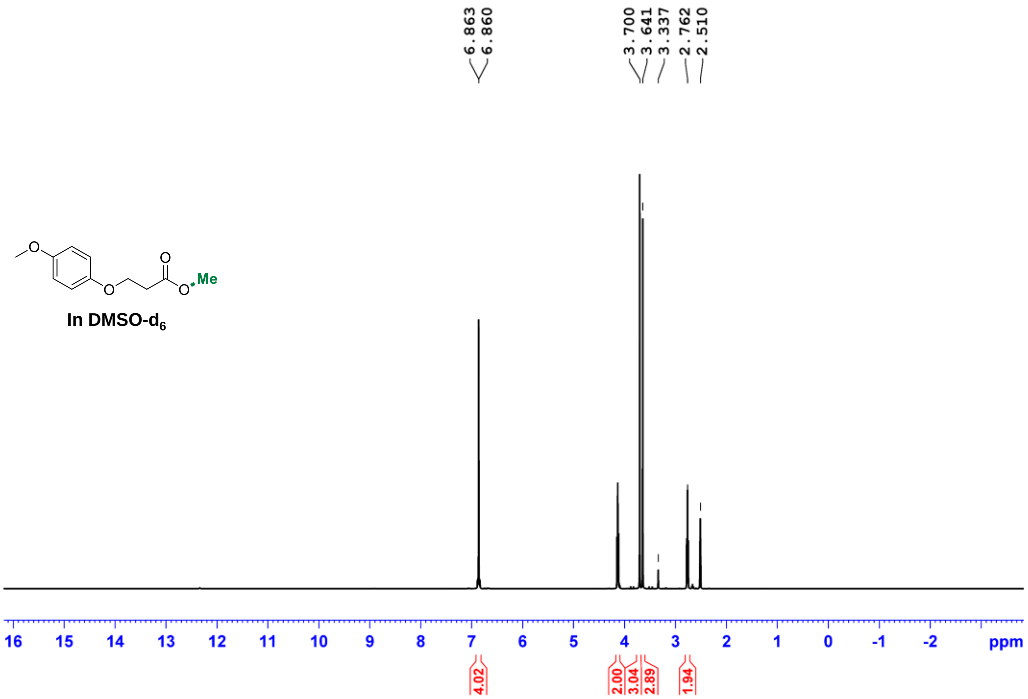

6.863
6.860
3.700
3.641
3.337
2.762
2.510
O
O
Me
In DMSO-d6
16 15 14 13 12 11 10 9 8 7 6 5 4 3 2 1 0 -1 -2 ppm
4.02
2.00
3.04
2.89
1.94

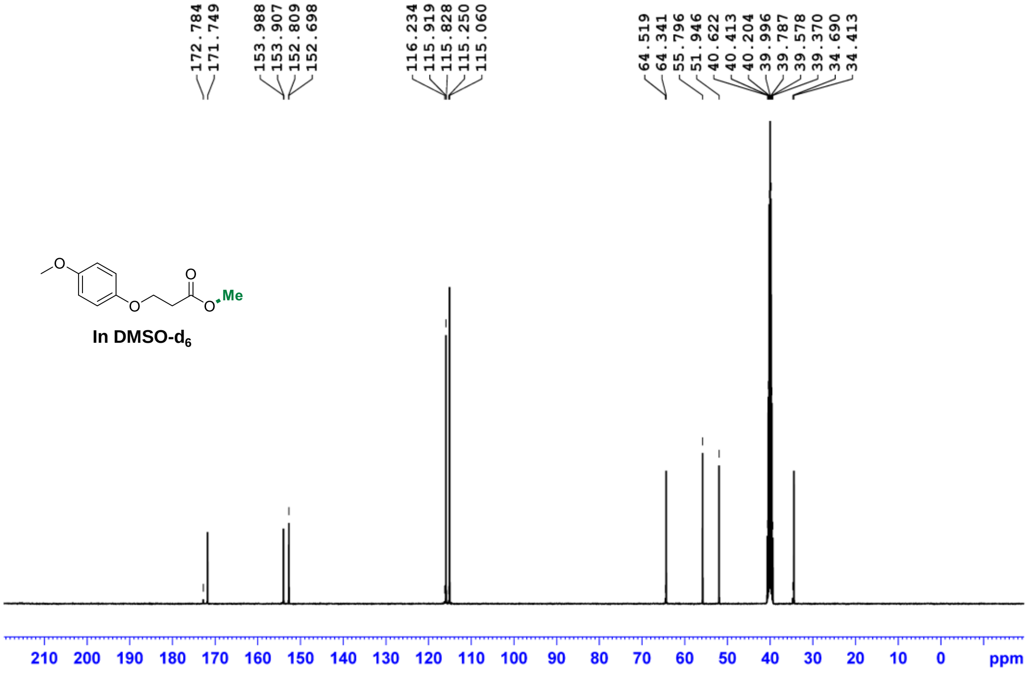

172.784
171.749
153.988
153.907
152.809
152.698
116.234
115.919
115.828
115.250
115.060
64.519
64.341
55.796
51.946
40.622
40.413
40.204
39.996
39.787
39.578
39.370
34.690
34.413
O
O
Me
In DMSO-d6
210 200 190 180 170 160 150 140 130 120 110 100 90 80 70 60 50 40 30 20 10 0 ppm

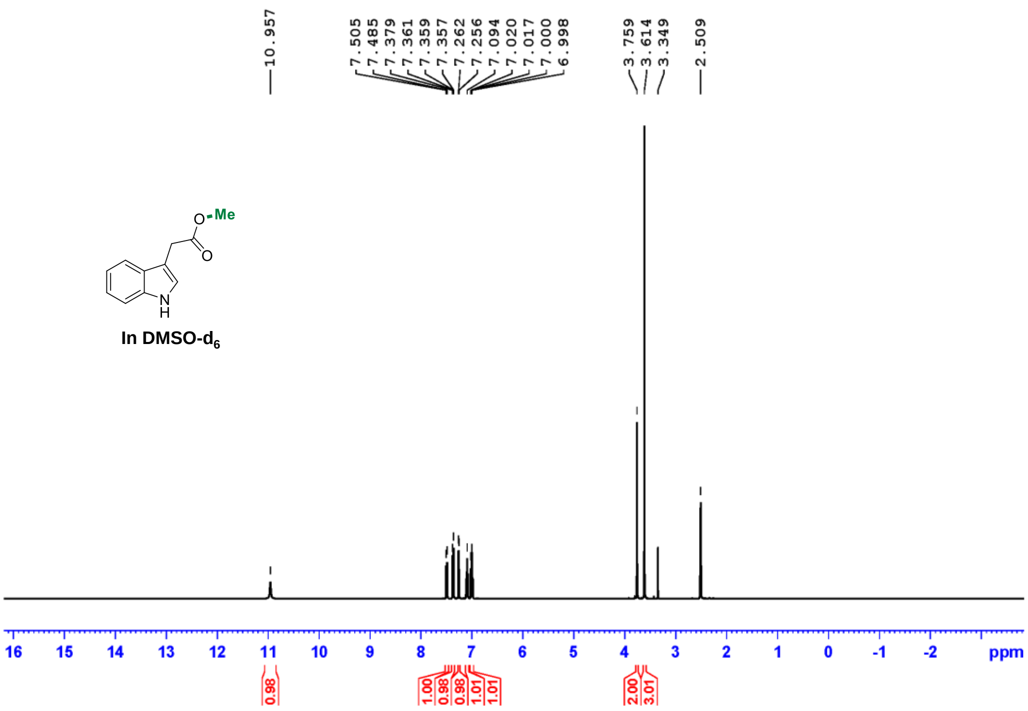
10.957
7.505
7.485
7.379
7.361
7.359
7.357
7.262
7.256
7.094
7.020
7.017
7.000
6.998
3.759
3.614
3.349
2.509
O
Me
O
N
H
In DMSO-d6
0.98
1.00
0.98
0.98
1.01
1.01
2.00
3.01
ppm

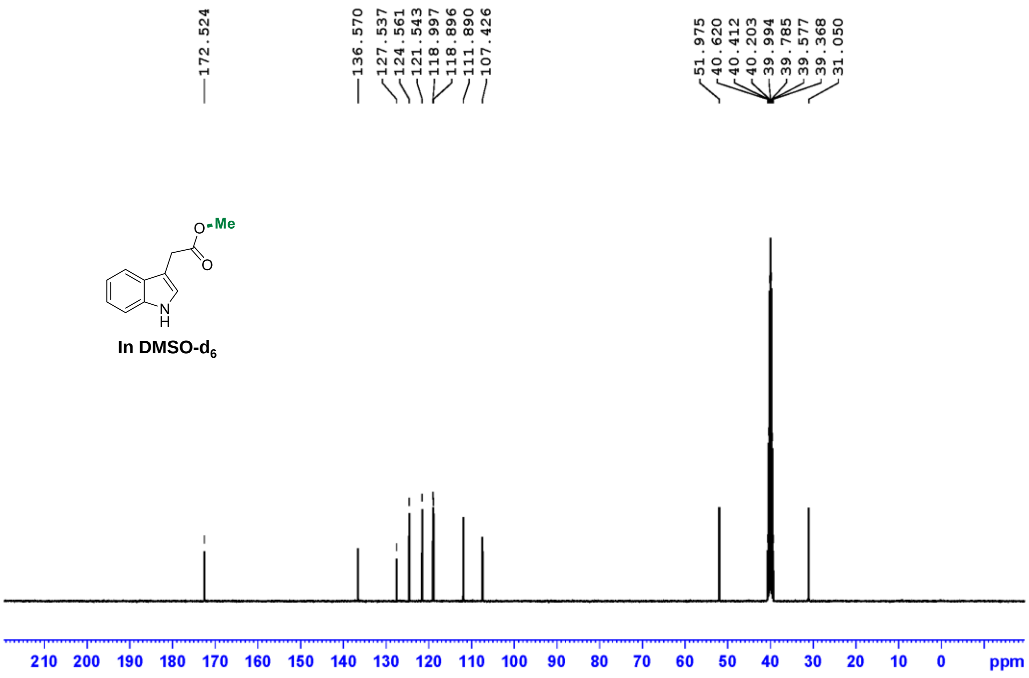
172.524
136.570
127.537
124.561
121.543
118.997
118.896
111.890
107.426
51.975
40.620
40.412
40.203
39.994
39.785
39.577
39.368
31.050
O
Me
O
N
H
In DMSO-d6
ppm

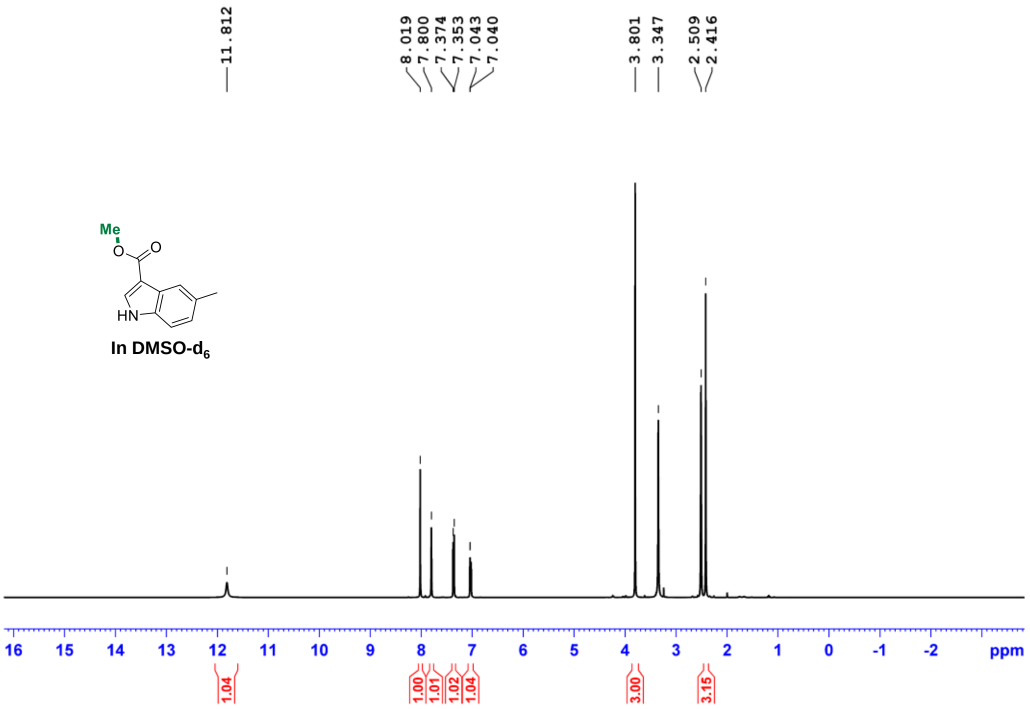
11.812
8.019
7.800
7.374
7.353
7.043
7.040
3.801
3.347
2.509
2.416
Me
O
O
HN
In DMSO-d6
16 15 14 13 12 11 10 9 8 7 6 5 4 3 2 1 0 -1 -2 ppm
1.04
1.00
1.01
1.02
1.04
3.00
3.15

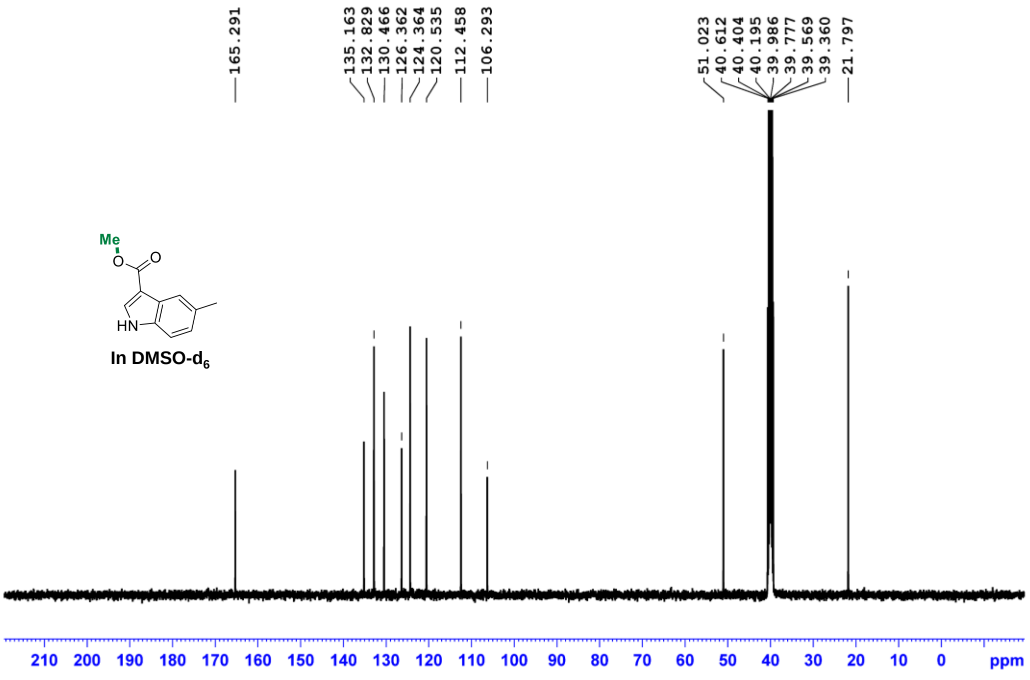
165.291
135.163
132.829
130.466
126.362
124.364
120.535
112.458
106.293
51.023
40.612
40.404
40.195
39.986
39.777
39.569
39.360
21.797
Me
O
O
HN
In DMSO-d6
210 200 190 180 170 160 150 140 130 120 110 100 90 80 70 60 50 40 30 20 10 0 ppm

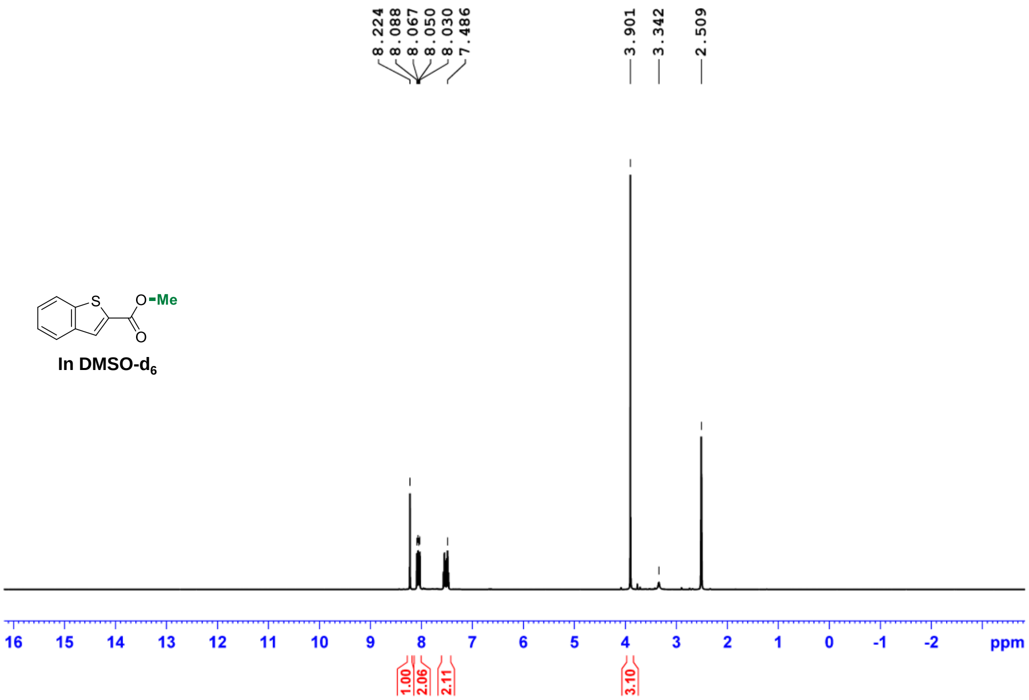

8.224
8.088
8.067
8.050
8.030
7.486
3.901
3.342
2.509
O
Me
S
O
In DMSO-d6
16
15
14
13
12
11
10
9
8
7
6
5
4
3
2
1
0
-1
-2
ppm
1.00
2.06
2.11
3.10

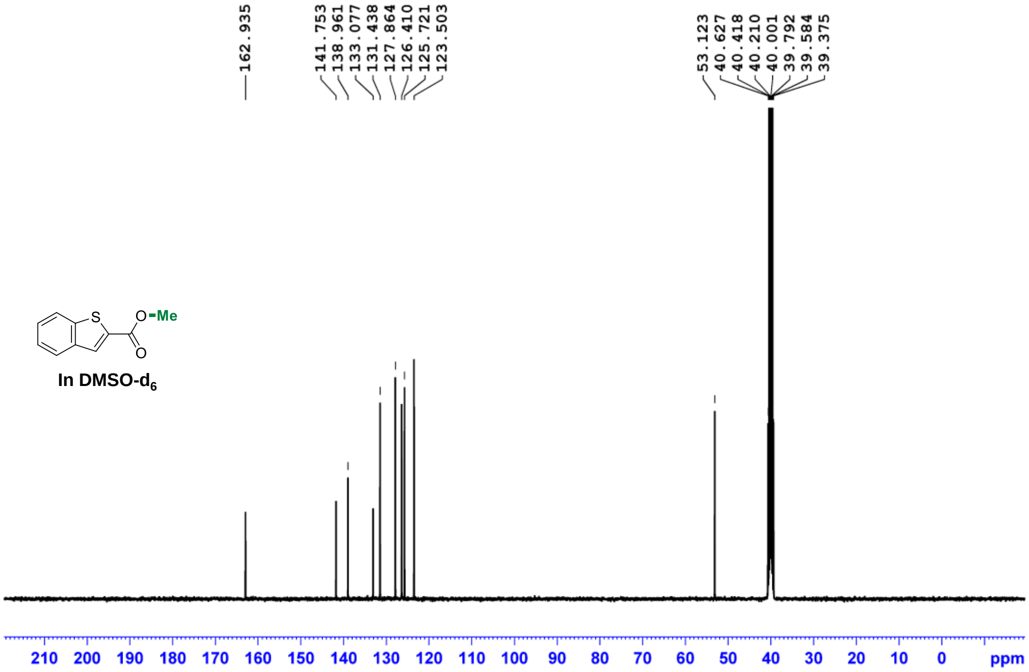

162.935
141.753
138.961
133.077
131.438
127.864
126.410
125.721
123.503
53.123
40.627
40.418
40.210
40.001
39.792
39.584
39.375
O
Me
S
O
In DMSO-d6
210
200
190
180
170
160
150
140
130
120
110
100
90
80
70
60
50
40
30
20
10
0
ppm

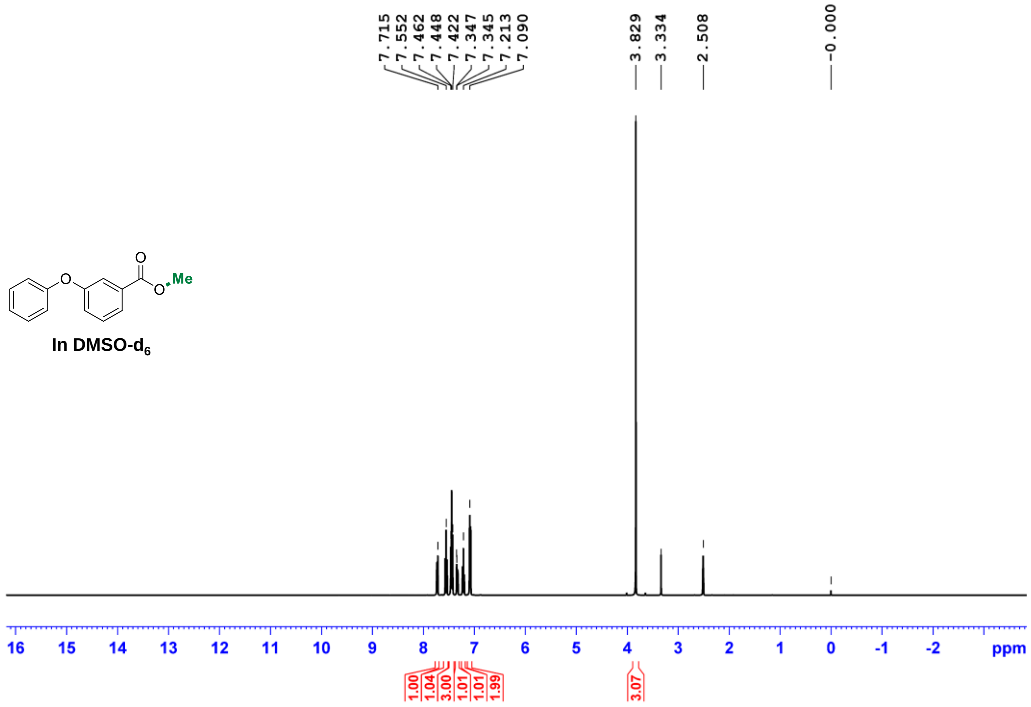

7.715
7.552
7.462
7.448
7.422
7.347
7.345
7.213
7.090
3.829
3.334
2.508
-0.000
O
O
Me
In DMSO-d6
16 15 14 13 12 11 10 9 8 7 6 5 4 3 2 1 0 -1 -2 ppm
1.00
1.04
3.00
1.01
1.01
1.99
3.07

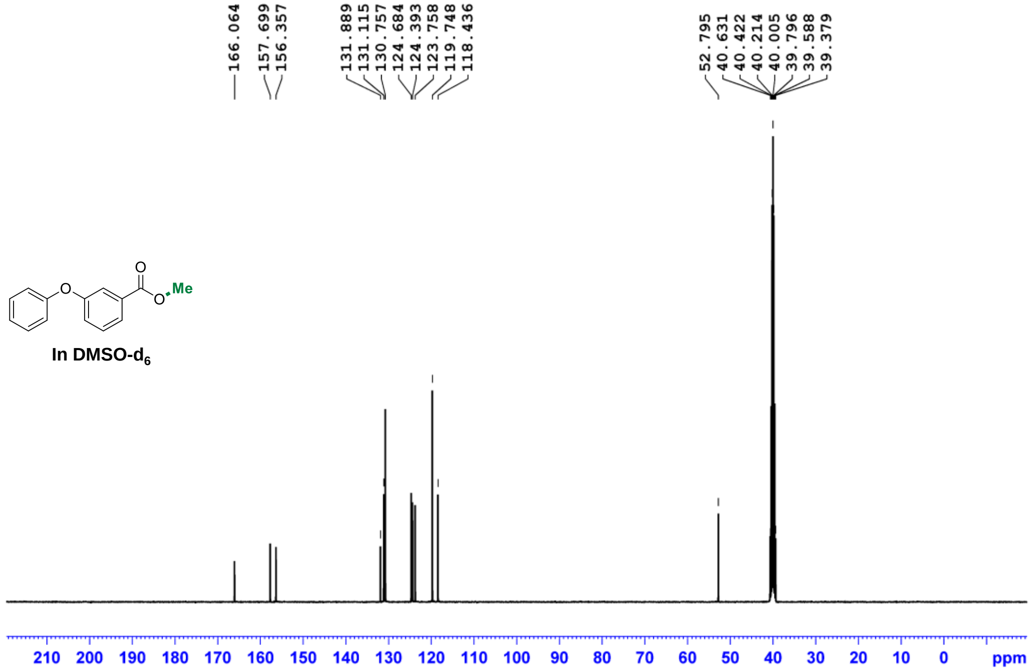

166.064
157.699
156.357
131.889
131.115
130.757
124.684
124.393
123.758
119.748
118.436
52.795
40.631
40.422
40.214
40.005
39.796
39.588
39.379
O
O
Me
In DMSO-d6
210 200 190 180 170 160 150 140 130 120 110 100 90 80 70 60 50 40 30 20 10 0 ppm

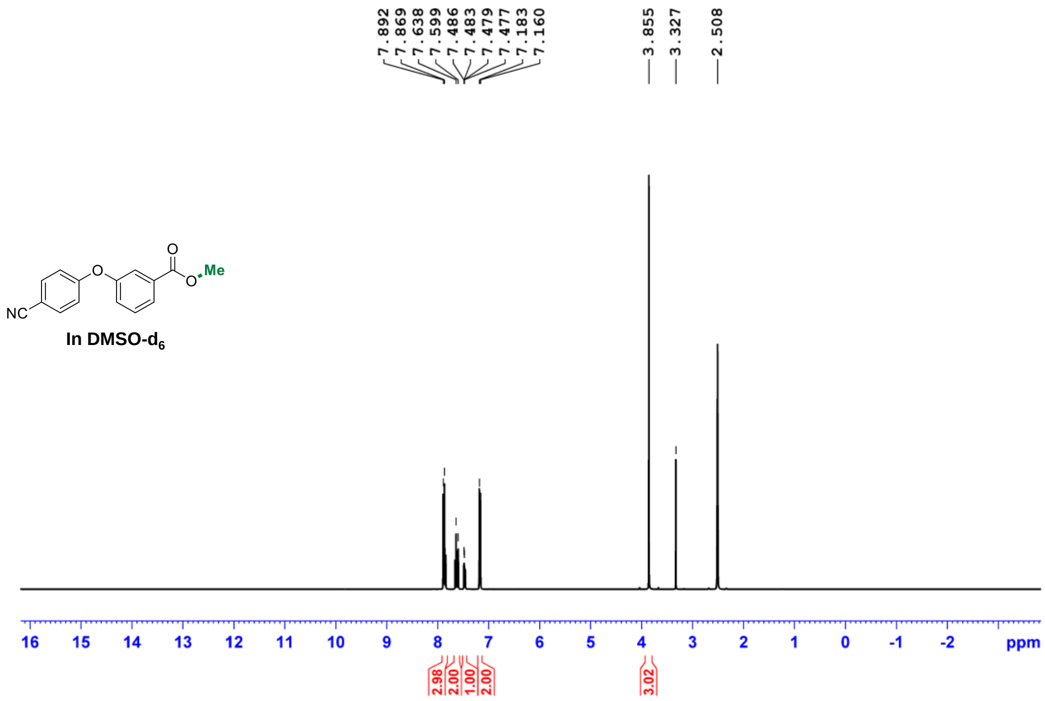

7.892
7.869
7.638
7.599
7.486
7.483
7.479
7.477
7.183
7.160
3.855
3.327
2.508
NC
O
O
O
Me
In DMSO-d6
16 15 14 13 12 11 10 9 8 7 6 5 4 3 2 1 0 -1 -2 ppm
2.98
2.00
1.00
2.00
3.02

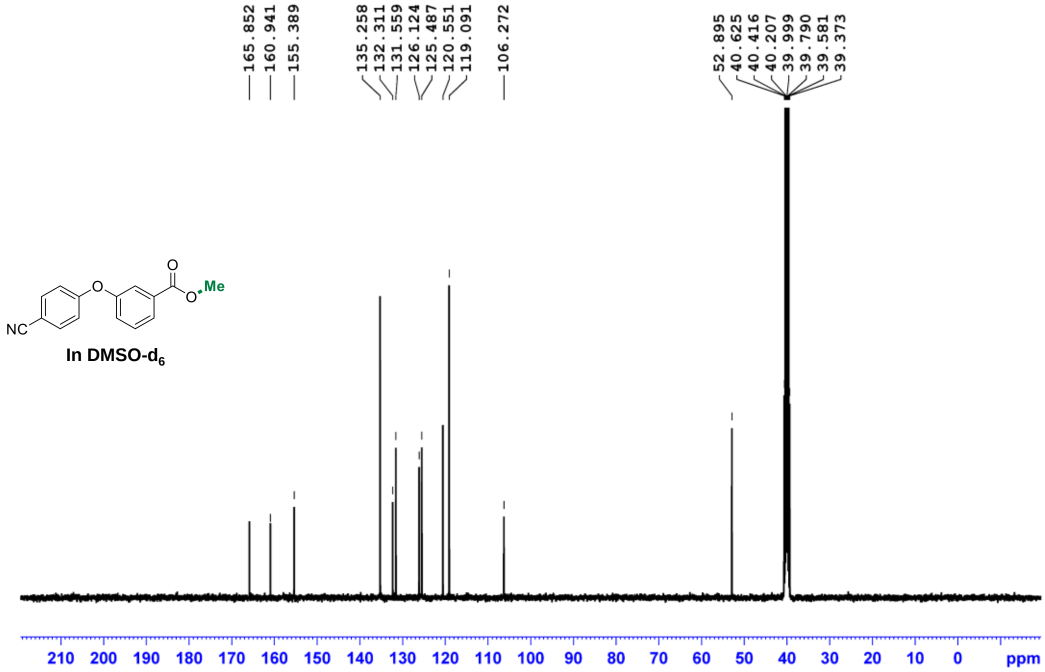

165.852
160.941
155.389
135.258
132.311
131.559
126.124
125.487
120.551
119.091
106.272
52.895
40.625
40.416
40.207
39.999
39.790
39.581
39.373
NC
O
O
O
Me
In DMSO-d6
210 200 190 180 170 160 150 140 130 120 110 100 90 80 70 60 50 40 30 20 10 0 ppm

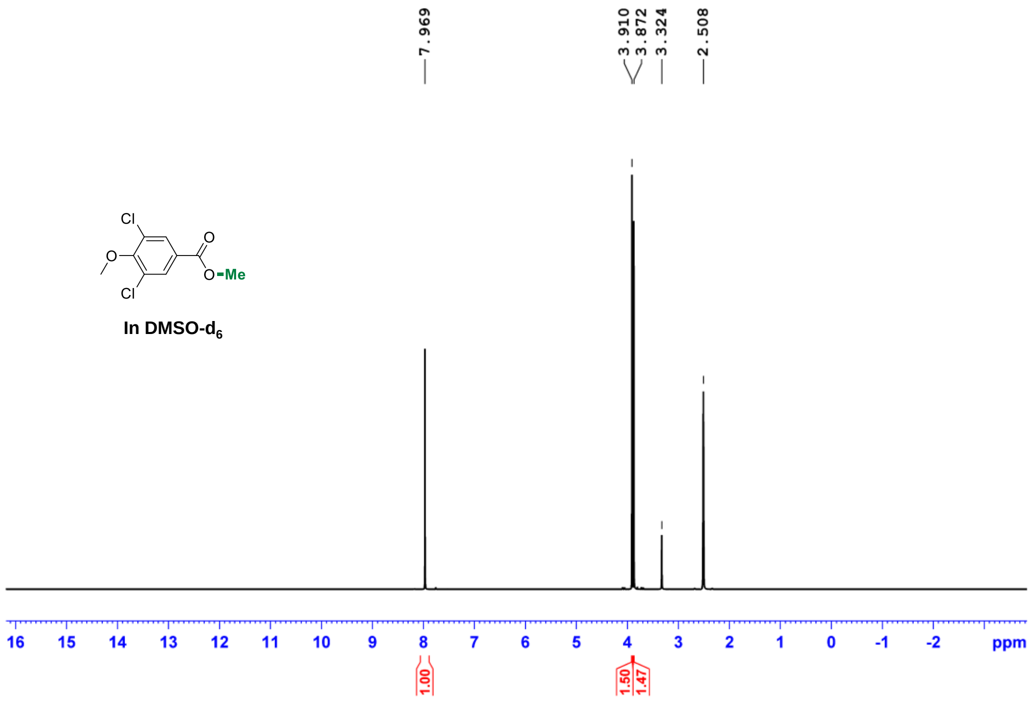
7.969
3.910
3.872
3.324
2.508
Cl
O
O
O
Me
Cl
In DMSO-d_6
16 15 14 13 12 11 10 9 8 7 6 5 4 3 2 1 0 -1 -2 ppm
1.00
1.50
1.47

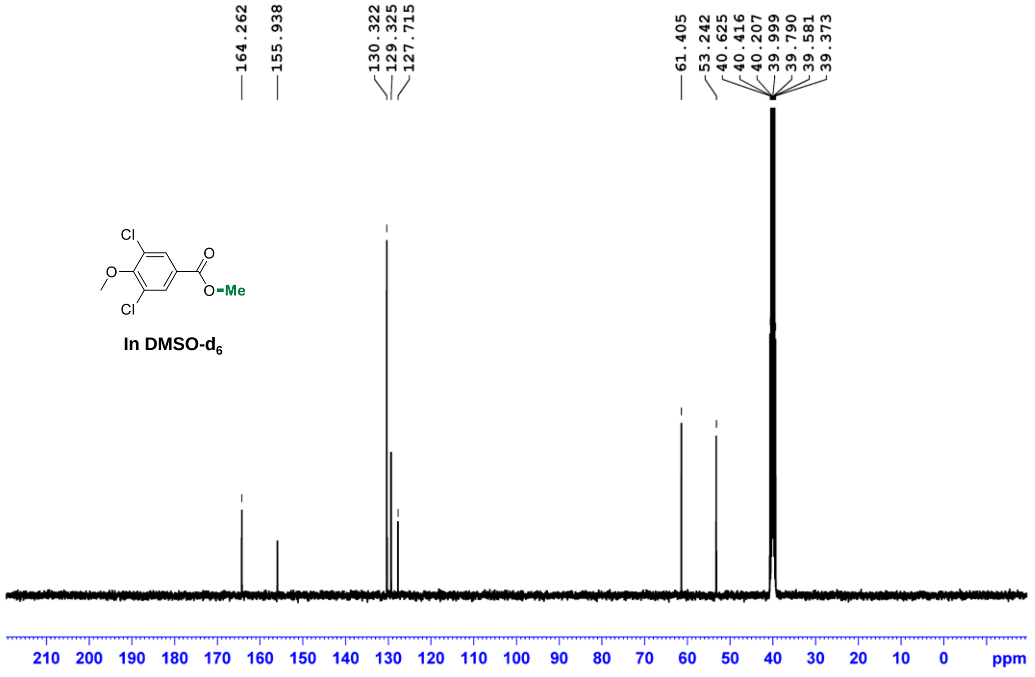
164.262
155.938
130.322
129.325
127.715
61.405
53.242
40.625
40.416
40.207
39.999
39.790
39.581
39.373
Cl
O
O
O
Me
Cl
In DMSO-d_6
210 200 190 180 170 160 150 140 130 120 110 100 90 80 70 60 50 40 30 20 10 0 ppm

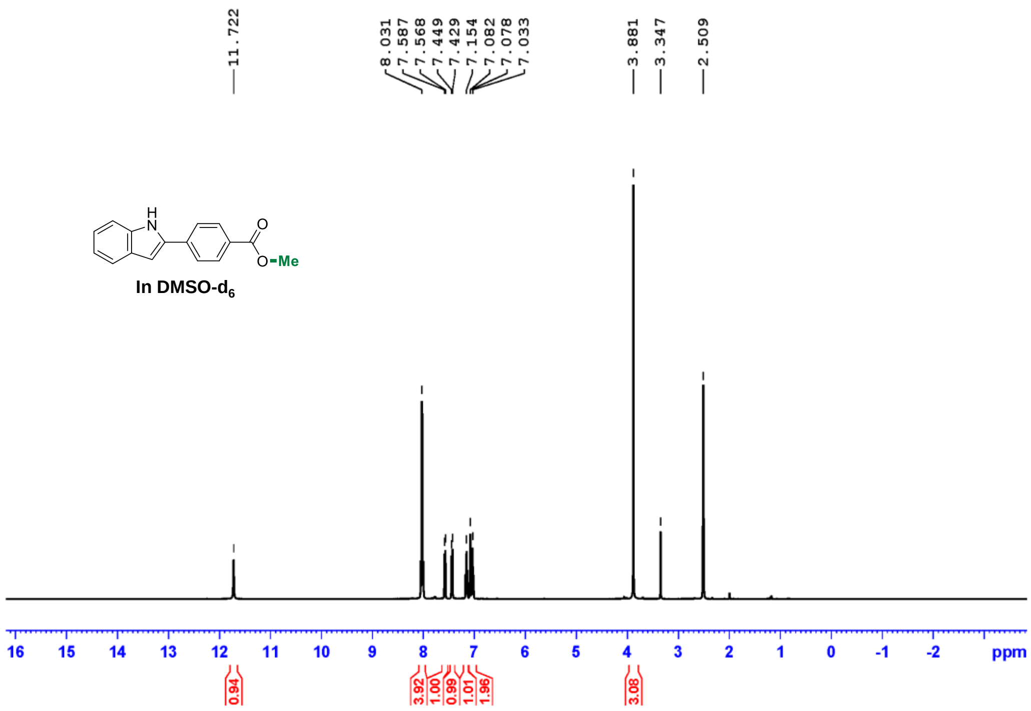
11.722
8.031
7.587
7.568
7.449
7.429
7.154
7.082
7.078
7.033
3.881
3.347
2.509
H
N
O
O
Me
In DMSO-d6
16 15 14 13 12 11 10 9 8 7 6 5 4 3 2 1 0 -1 -2 ppm
0.94
3.92
1.00
0.99
1.01
1.96
3.08

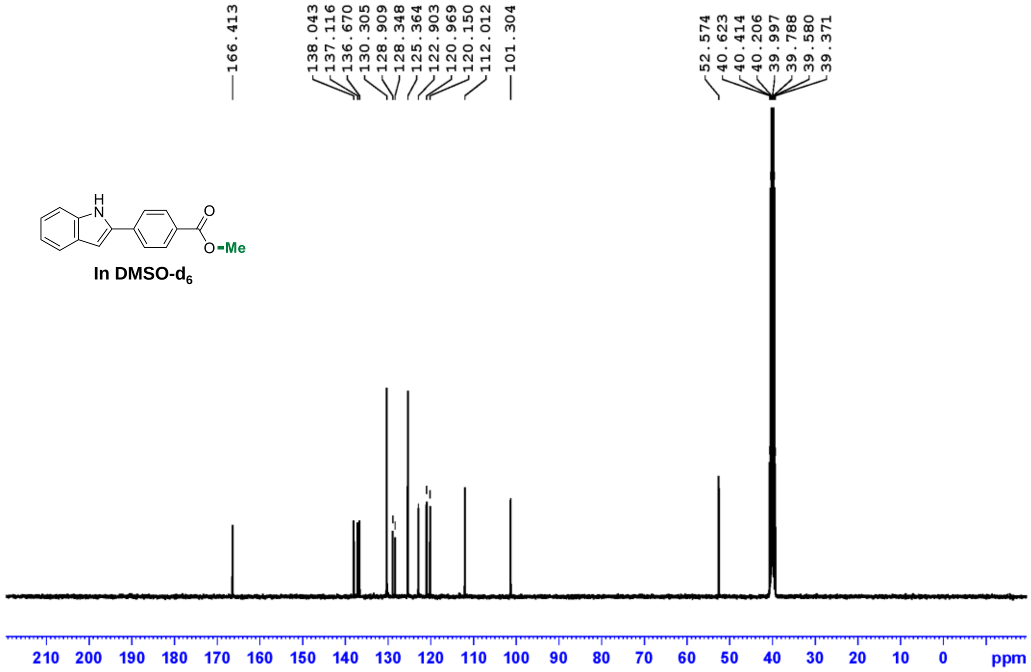
166.413
138.043
137.116
136.670
130.305
128.909
128.348
125.364
122.903
120.969
120.150
112.012
101.304
52.574
40.623
40.414
40.206
39.997
39.788
39.580
39.371
H
N
O
O
Me
In DMSO-d6
210 200 190 180 170 160 150 140 130 120 110 100 90 80 70 60 50 40 30 20 10 0 ppm

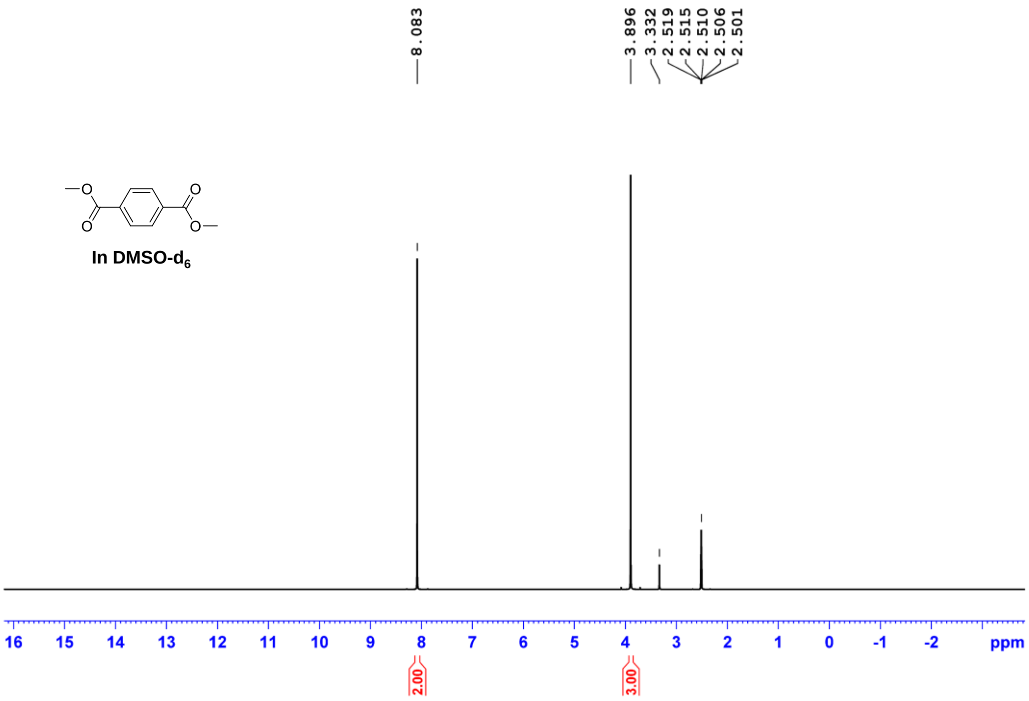
8.083
3.896
3.332
2.519
2.515
2.510
2.506
2.501
In DMSO-d6
16 15 14 13 12 11 10 9 8 7 6 5 4 3 2 1 0 -1 -2 ppm
2.00
3.00

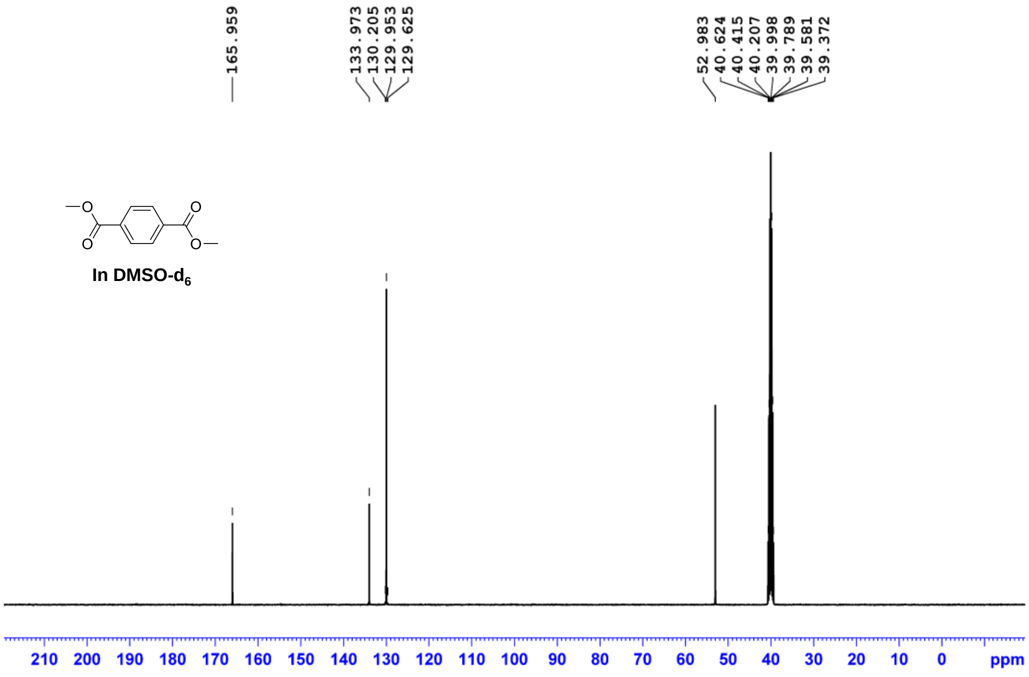
165.959
133.973
130.205
129.953
129.625
52.983
40.624
40.415
40.207
39.998
39.789
39.581
39.372
In DMSO-d6
210 200 190 180 170 160 150 140 130 120 110 100 90 80 70 60 50 40 30 20 10 0 ppm

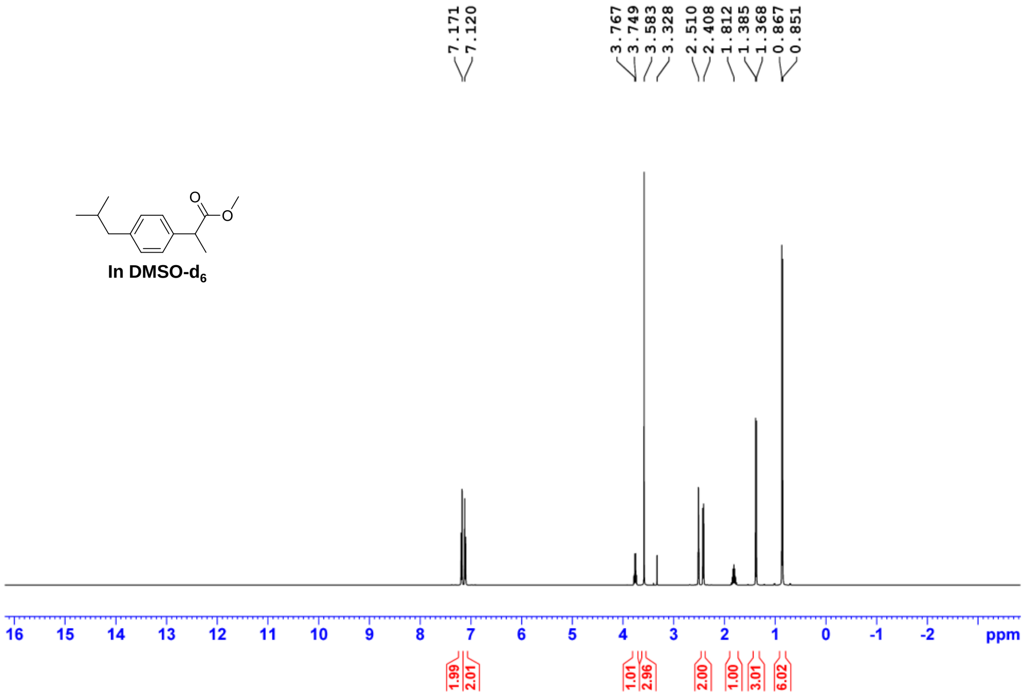
7.171
7.120
3.767
3.749
3.583
3.328
2.510
2.408
1.812
1.385
1.368
0.867
0.851
In DMSO-d6
16 15 14 13 12 11 10 9 8 7 6 5 4 3 2 1 0 -1 -2 ppm
1.99
2.01
1.01
2.96
2.00
1.00
3.01
6.02

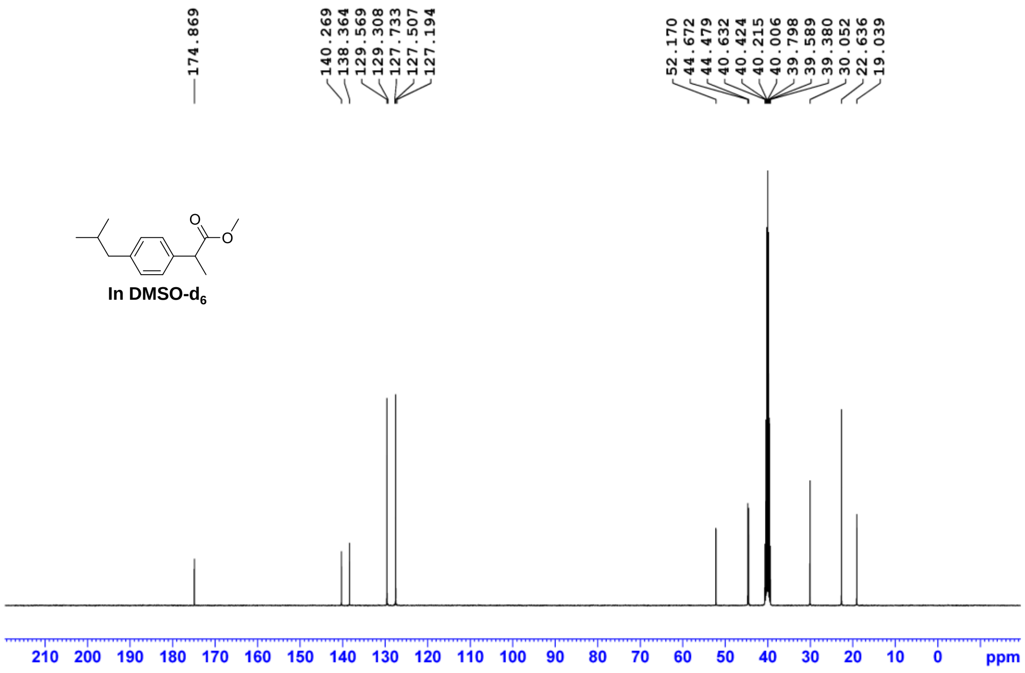
174.869
140.269
138.364
129.569
129.308
127.733
127.507
127.194
52.170
44.672
44.479
40.632
40.424
40.215
40.006
39.798
39.589
39.380
30.052
22.636
19.039
In DMSO-d6
210 200 190 180 170 160 150 140 130 120 110 100 90 80 70 60 50 40 30 20 10 0 ppm

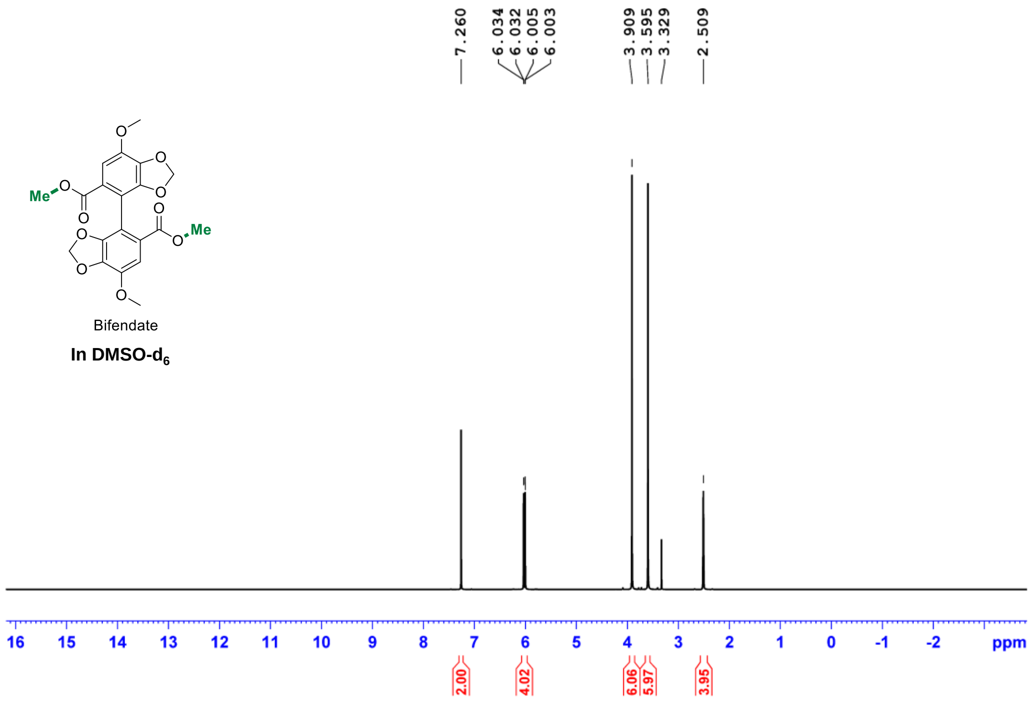
7.260
6.034
6.032
6.005
6.003
3.909
3.595
3.329
2.509
Me
O
O
O
O
O
O
O
O
Me
Bifendate
In DMSO-d6
16 15 14 13 12 11 10 9 8 7 6 5 4 3 2 1 0 -1 -2 ppm
2.00
4.02
6.06
5.97
3.95

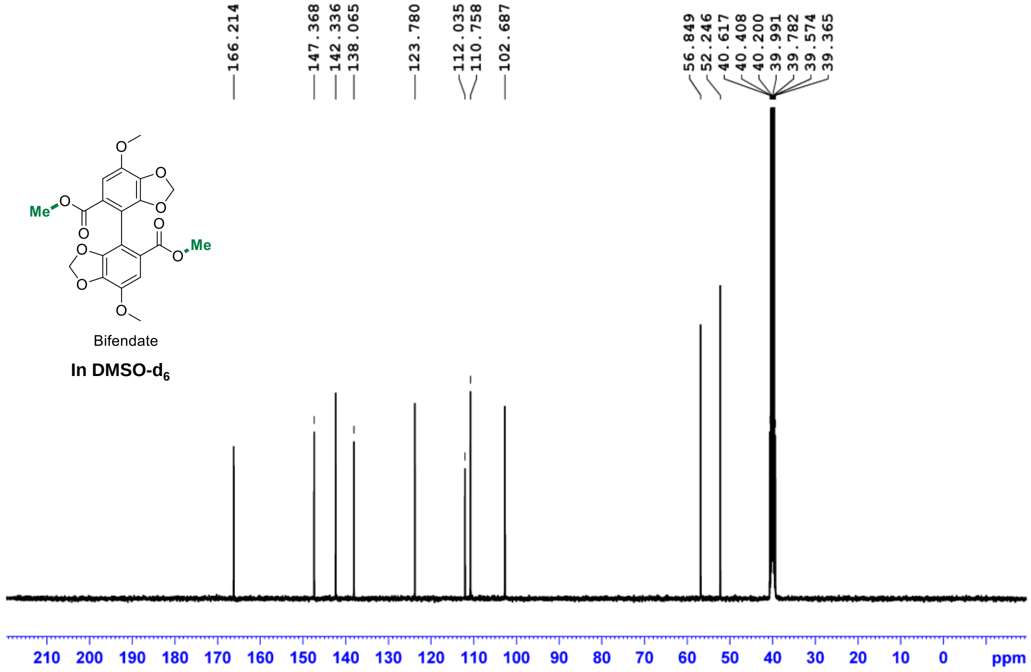
166.214
147.368
142.336
138.065
123.780
112.035
110.758
102.687
56.849
52.246
40.617
40.408
40.200
39.991
39.782
39.574
39.365
Me
O
O
O
O
O
O
O
O
Me
Bifendate
In DMSO-d6
210 200 190 180 170 160 150 140 130 120 110 100 90 80 70 60 50 40 30 20 10 0 ppm

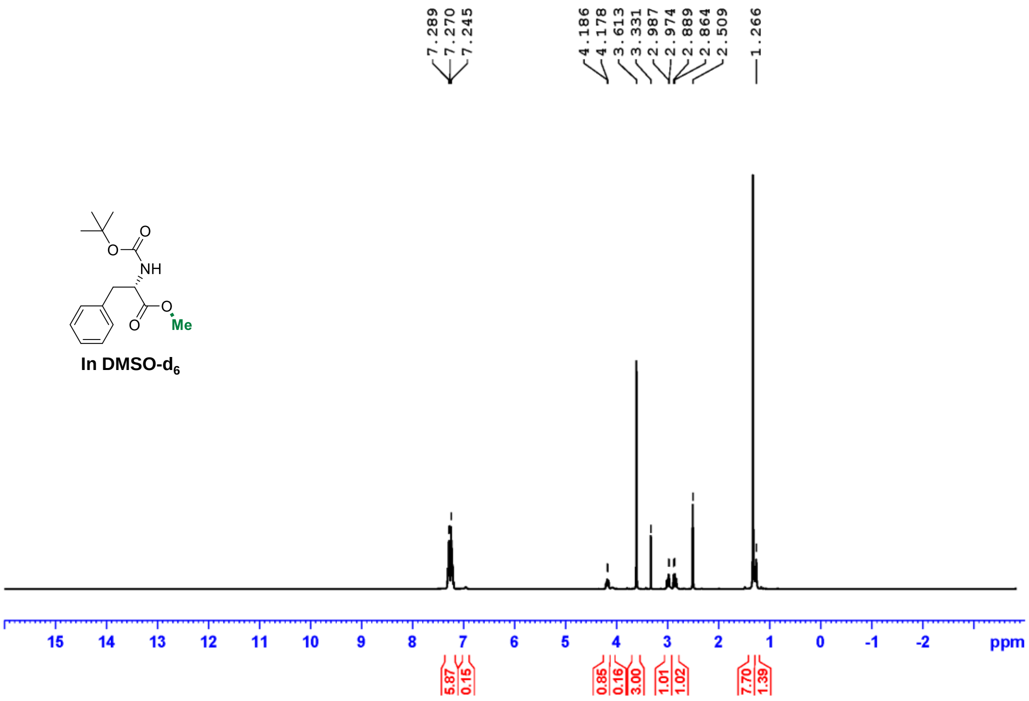
7.289
7.270
7.245
4.186
4.178
3.613
3.331
2.987
2.974
2.889
2.864
2.509
1.266
NH
O
O
O
O
Me
In DMSO-d_6
15 14 13 12 11 10 9 8 7 6 5 4 3 2 1 0 -1 -2 ppm
5.87
0.15
0.85
0.16
3.00
1.01
1.02
7.70
1.39

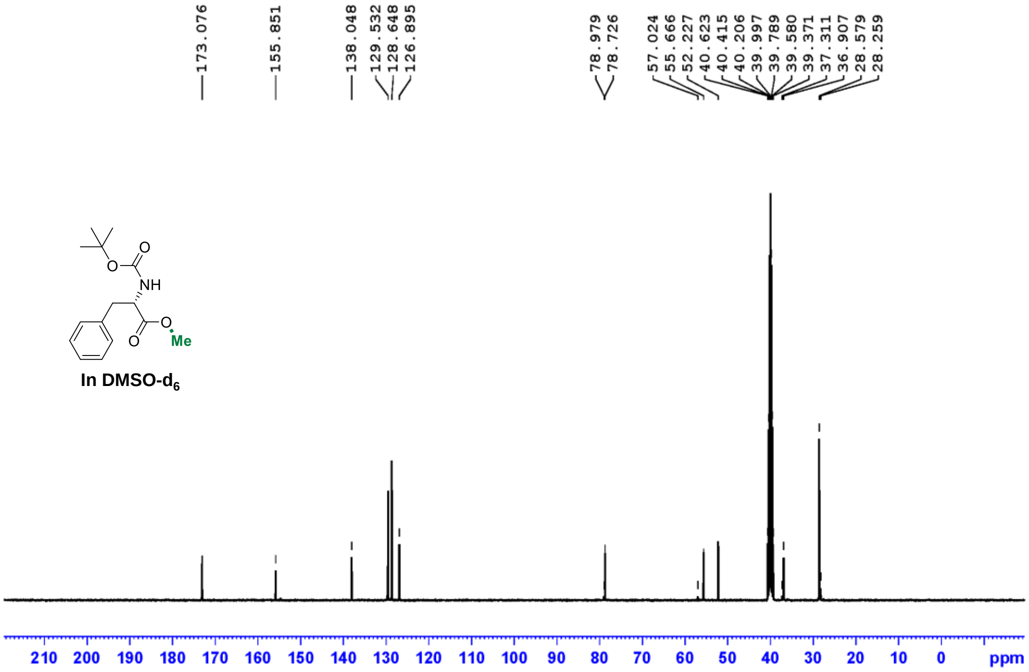
173.076
155.851
138.048
129.532
128.648
126.895
78.979
78.726
57.024
55.666
52.227
40.623
40.415
40.206
39.997
39.789
39.580
39.371
37.311
36.907
28.579
28.259
NH
O
O
O
O
Me
In DMSO-d_6
210 200 190 180 170 160 150 140 130 120 110 100 90 80 70 60 50 40 30 20 10 0 ppm

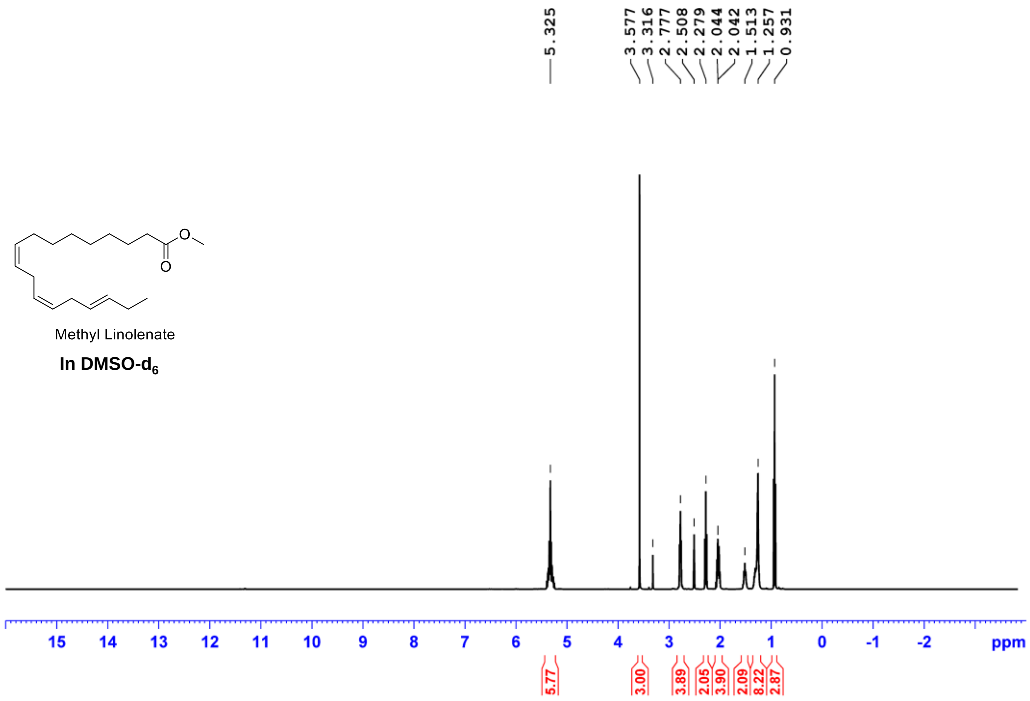
5.325
3.577
3.316
2.777
2.508
2.279
2.044
2.042
1.513
1.257
0.931
Methyl Linolenate
In DMSO-d6
15 14 13 12 11 10 9 8 7 6 5 4 3 2 1 0 -1 -2 ppm
5.77
3.00
3.89
2.05
3.90
2.09
8.22
2.87

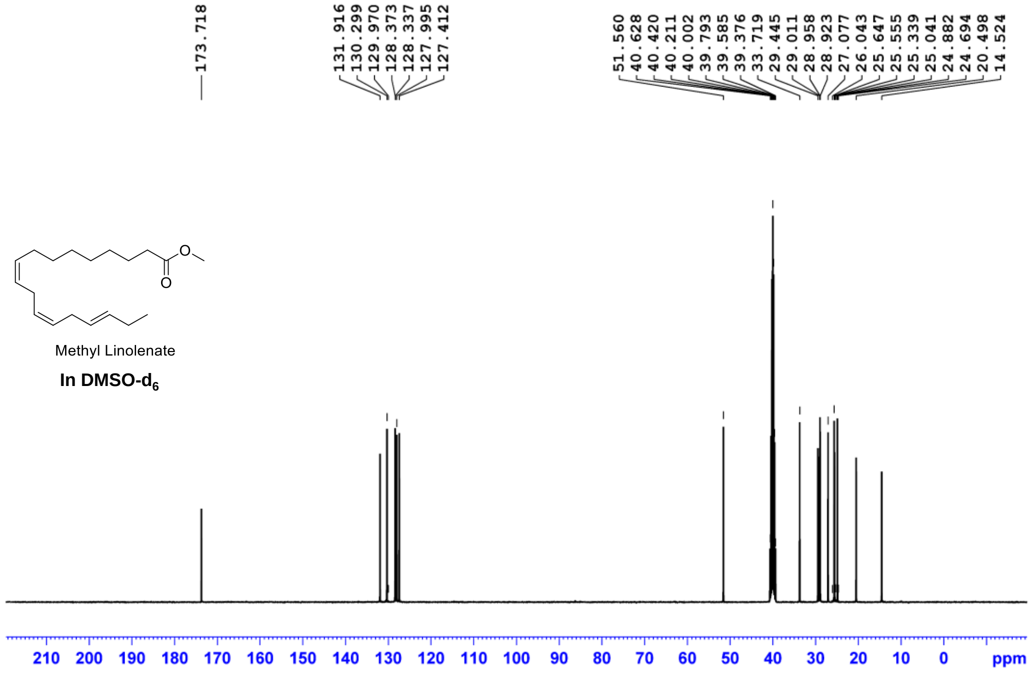
173.718
131.916
130.299
129.970
128.373
128.337
127.995
127.412
51.560
40.628
40.420
40.211
40.002
39.793
39.585
39.376
33.719
29.445
29.011
28.958
28.923
27.077
26.043
25.647
25.555
25.339
25.041
24.882
24.694
20.498
14.524
Methyl Linolenate
In DMSO-d6
210 200 190 180 170 160 150 140 130 120 110 100 90 80 70 60 50 40 30 20 10 0 ppm

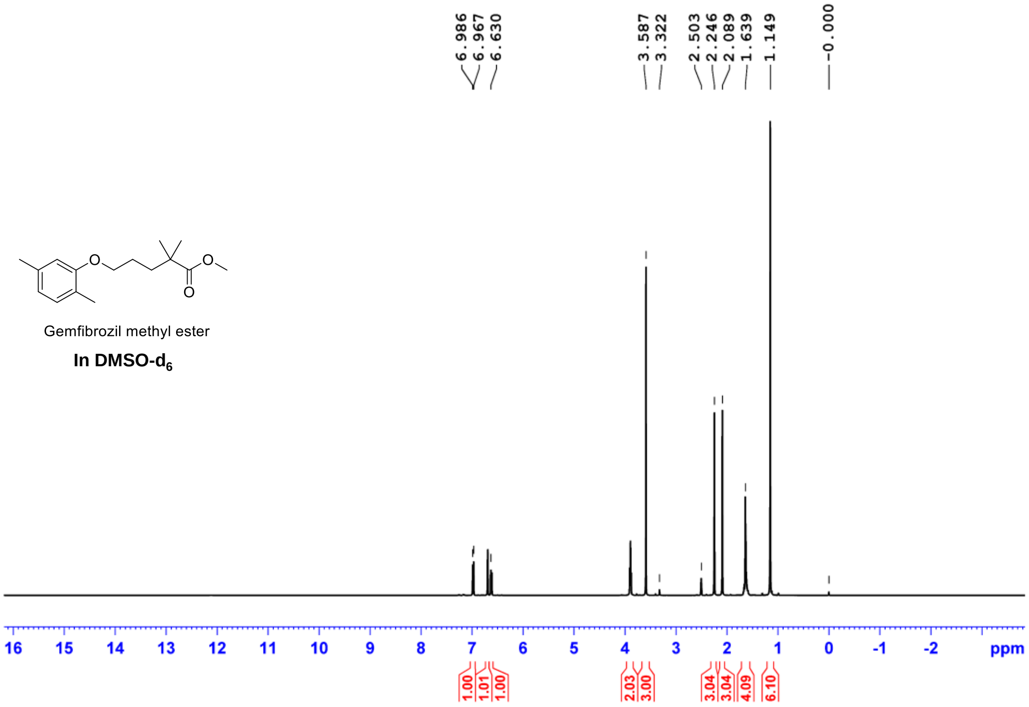

6.986
6.967
6.630
3.587
3.322
2.503
2.246
2.089
1.639
1.149
-0.000
Gemfibrozil methyl ester
In DMSO-d6
16 15 14 13 12 11 10 9 8 7 6 5 4 3 2 1 0 -1 -2 ppm
1.00
1.01
1.00
2.03
3.00
3.04
3.04
4.09
6.10

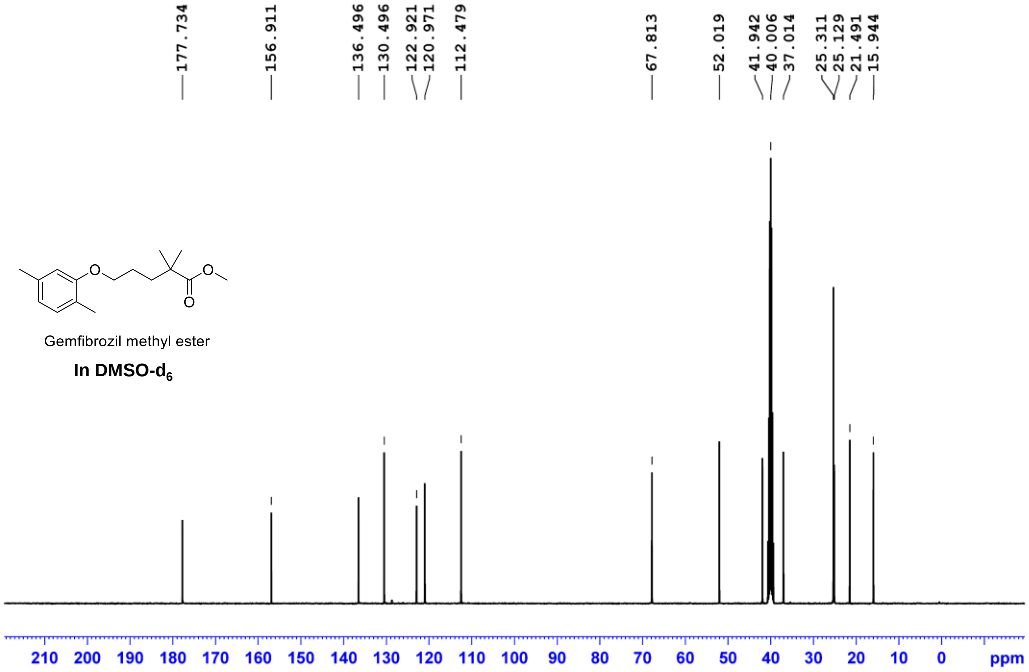

177.734
156.911
136.496
130.496
122.921
120.971
112.479
67.813
52.019
41.942
40.006
37.014
25.311
25.129
21.491
15.944
Gemfibrozil methyl ester
In DMSO-d6
210 200 190 180 170 160 150 140 130 120 110 100 90 80 70 60 50 40 30 20 10 0 ppm

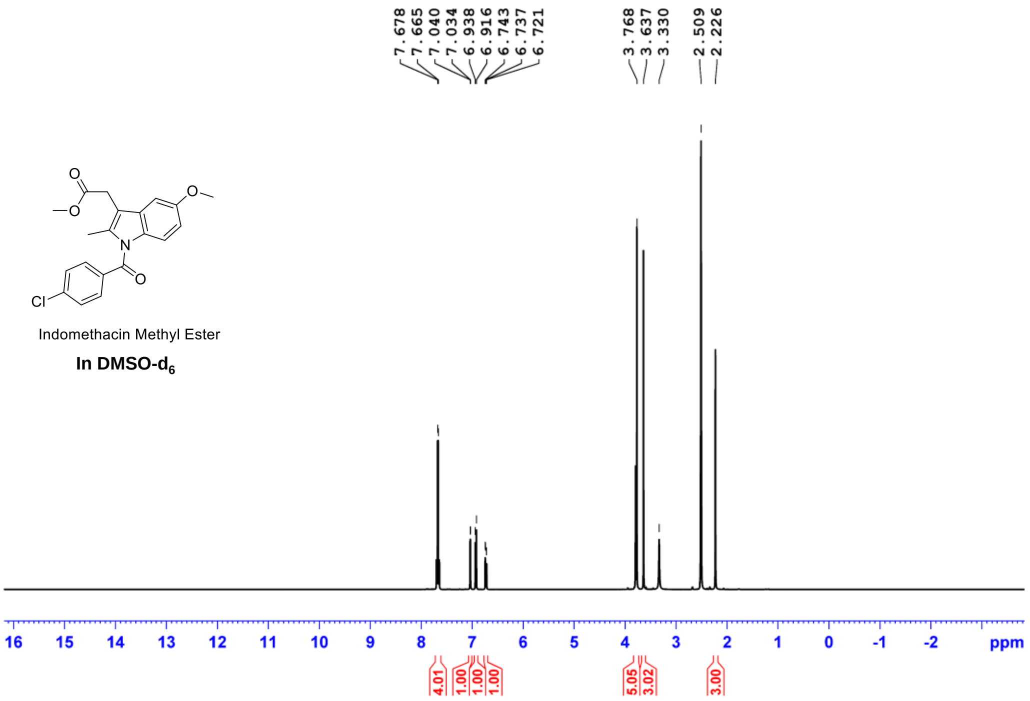

7.678
7.665
7.040
7.034
6.938
6.916
6.743
6.737
6.721
3.768
3.637
3.330
2.509
2.226
Indomethacin Methyl Ester
In DMSO-d6
4.01
1.00
1.00
1.00
1.00
5.05
3.02
3.00
16 15 14 13 12 11 10 9 8 7 6 5 4 3 2 1 0 -1 -2 ppm

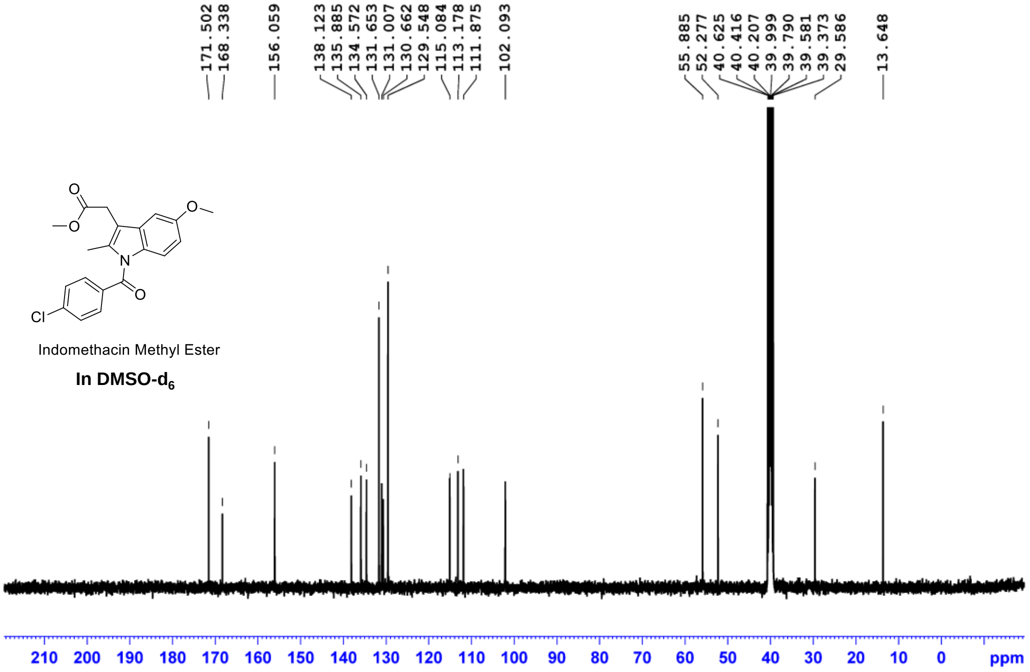

171.502
168.338
156.059
138.123
135.885
134.572
131.653
131.007
130.662
129.548
115.084
113.178
111.875
102.093
55.885
52.277
40.625
40.416
40.207
39.999
39.790
39.581
39.373
29.586
13.648
Indomethacin Methyl Ester
In DMSO-d6
210 200 190 180 170 160 150 140 130 120 110 100 90 80 70 60 50 40 30 20 10 0 ppm

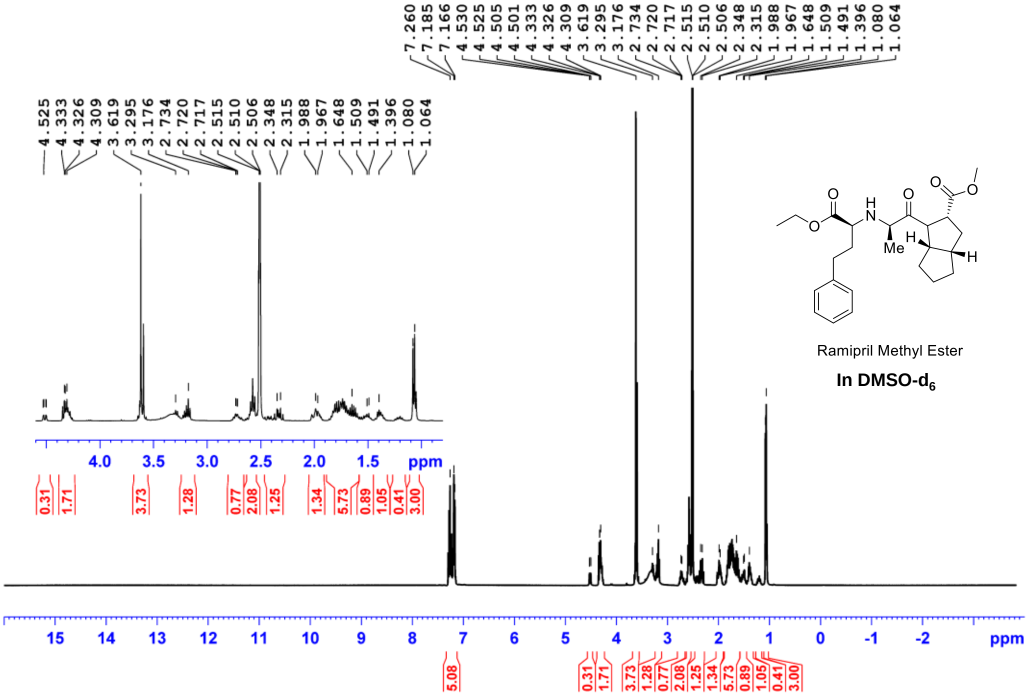

Ramipril Methyl Ester
In DMSO-d6

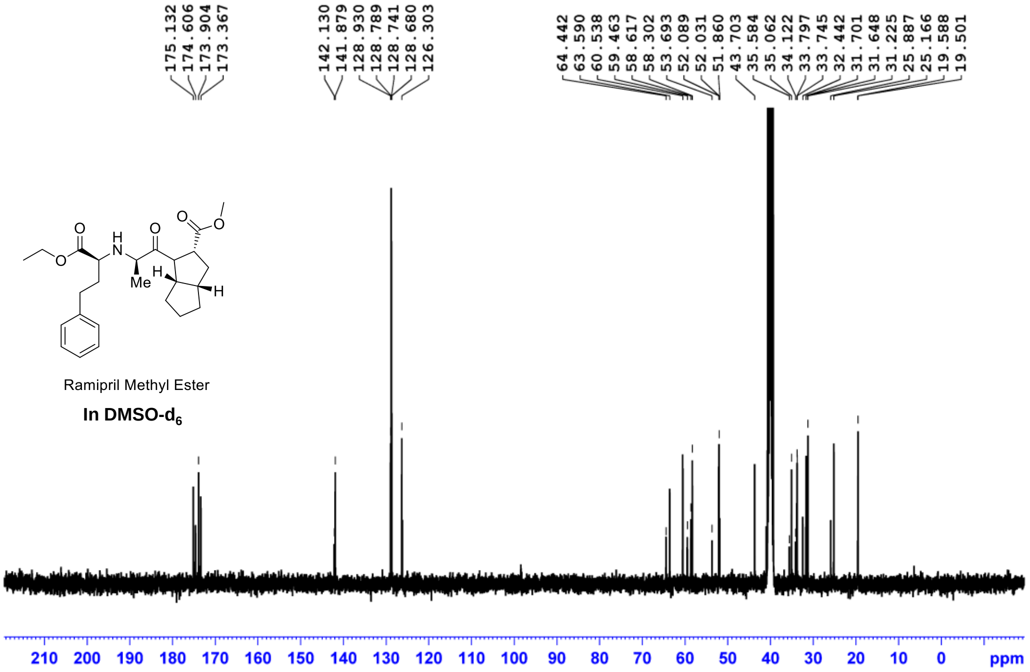

Ramipril Methyl Ester
In DMSO-d6

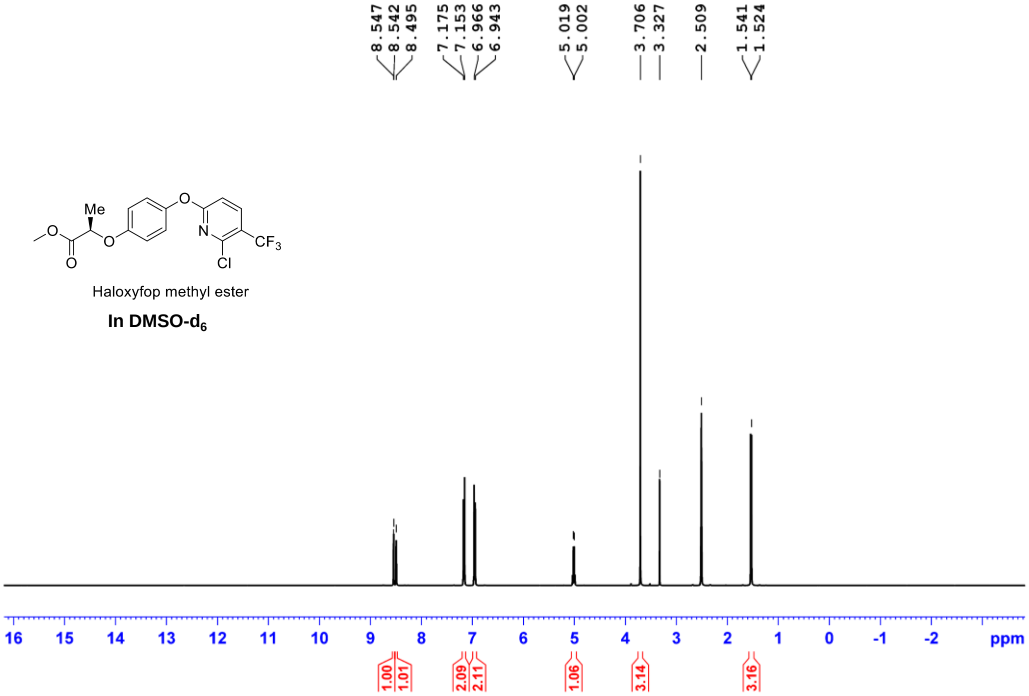
8.547
8.542
8.495
7.175
7.153
6.966
6.943
5.019
5.002
3.706
3.327
2.509
1.541
1.524
Me
O
N
CF3
Cl
Haloxyfop methyl ester
In DMSO-d6
16 15 14 13 12 11 10 9 8 7 6 5 4 3 2 1 0 -1 -2 ppm
1.00
1.01
2.09
2.11
1.06
3.14
3.16

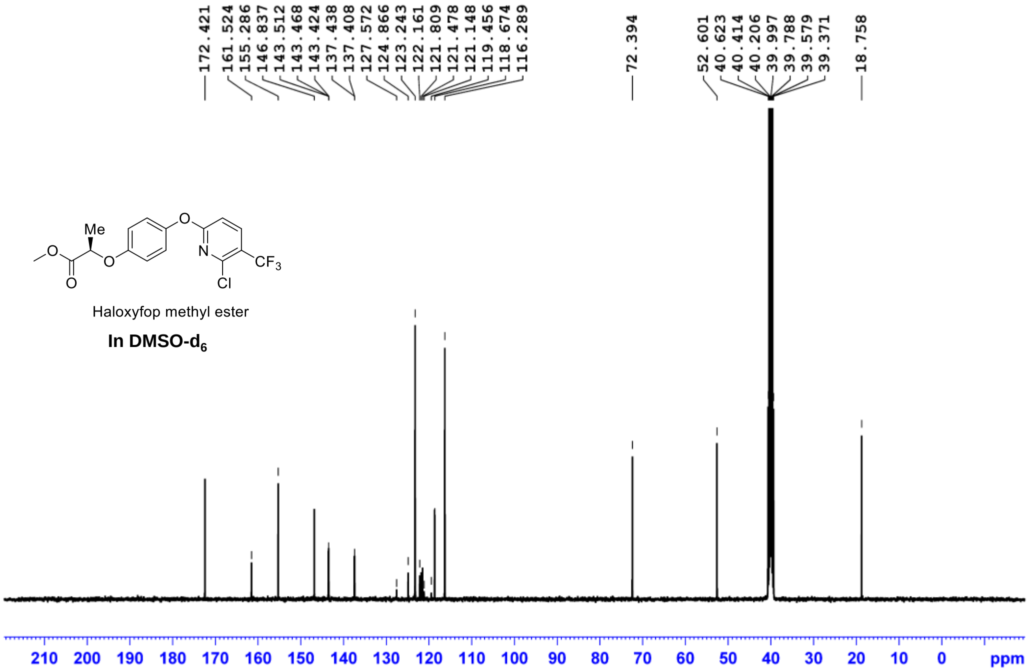
172.421
161.524
155.286
146.837
143.512
143.468
143.424
137.438
137.408
127.572
124.866
123.243
122.161
121.809
121.478
121.148
119.456
118.674
116.289
72.394
52.601
40.623
40.414
40.206
39.997
39.788
39.579
39.371
18.758
Me
O
N
CF3
Cl
Haloxyfop methyl ester
In DMSO-d6
210 200 190 180 170 160 150 140 130 120 110 100 90 80 70 60 50 40 30 20 10 0 ppm

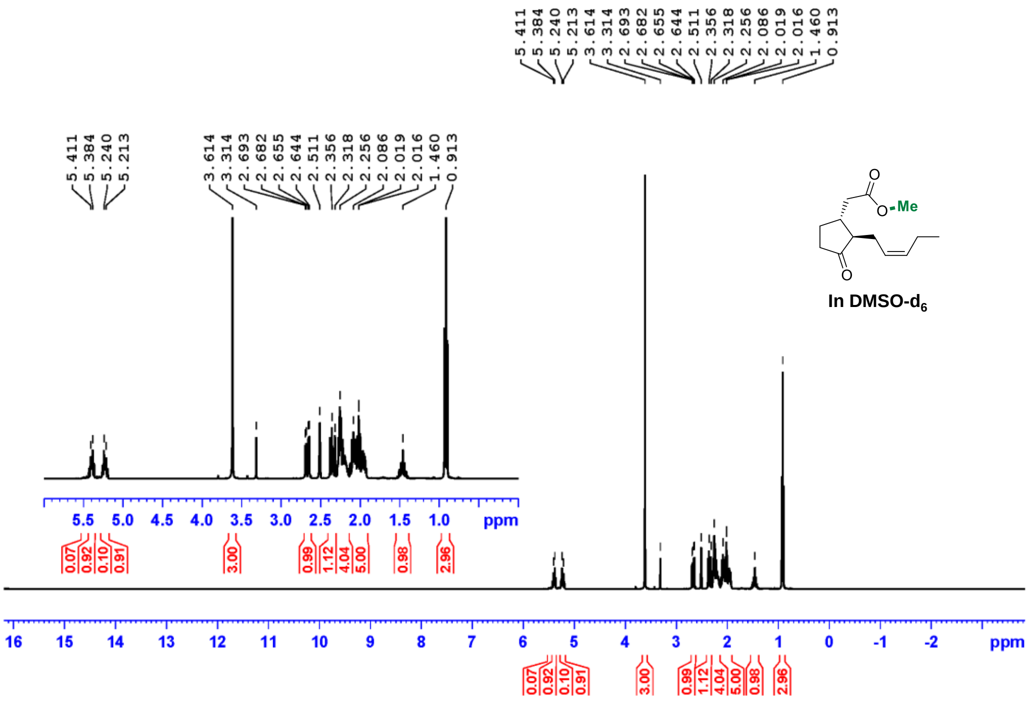
5.411
5.384
5.240
5.213
3.614
3.314
2.693
2.682
2.655
2.644
2.511
2.356
2.318
2.256
2.086
2.019
2.016
1.460
0.913
O
O
Me
In DMSO-d_6
0.07
0.92
0.10
0.91
3.00
0.99
1.12
4.04
5.00
0.98
2.96
ppm

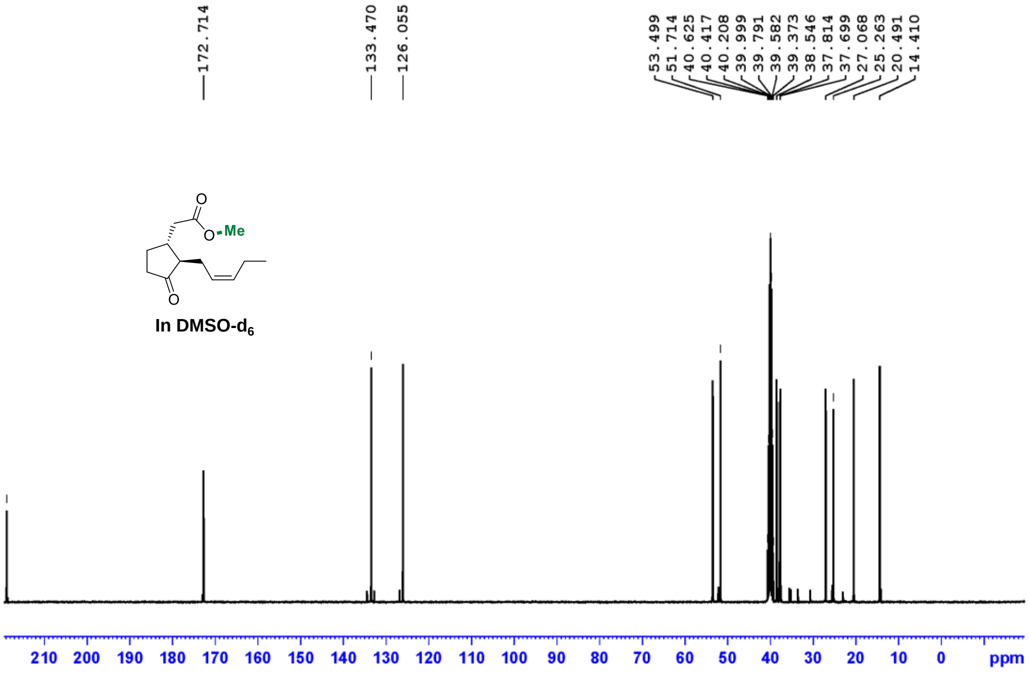
172.714
133.470
126.055
53.499
51.714
40.625
40.417
40.208
39.999
39.791
39.582
39.373
38.546
37.814
37.699
27.068
25.263
20.491
14.410
O
O
Me
In DMSO-d_6
ppm

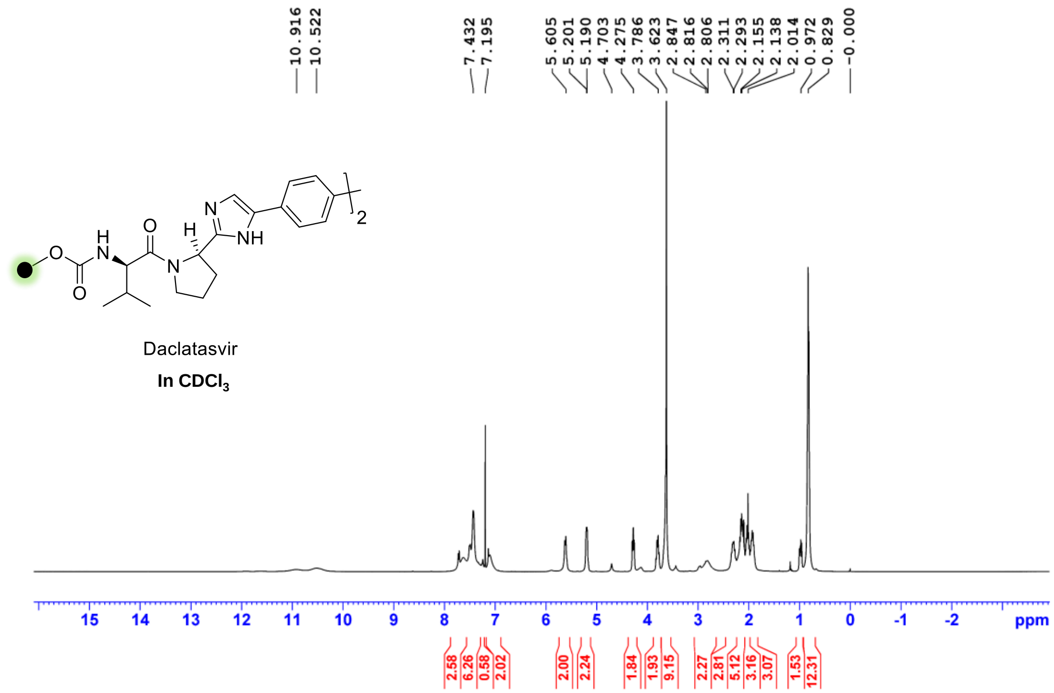
10.916
10.522
7.432
7.195
5.605
5.201
5.190
4.703
4.275
3.786
3.623
2.847
2.816
2.806
2.311
2.293
2.155
2.138
2.014
0.972
0.829
-0.000
Daclatasvir
In CDCl3
15 14 13 12 11 10 9 8 7 6 5 4 3 2 1 0 -1 -2 ppm
2.58 6.26 0.58 2.02 2.00 2.24 1.84 1.93 9.15 2.27 2.81 5.12 3.16 3.07 1.53 12.31

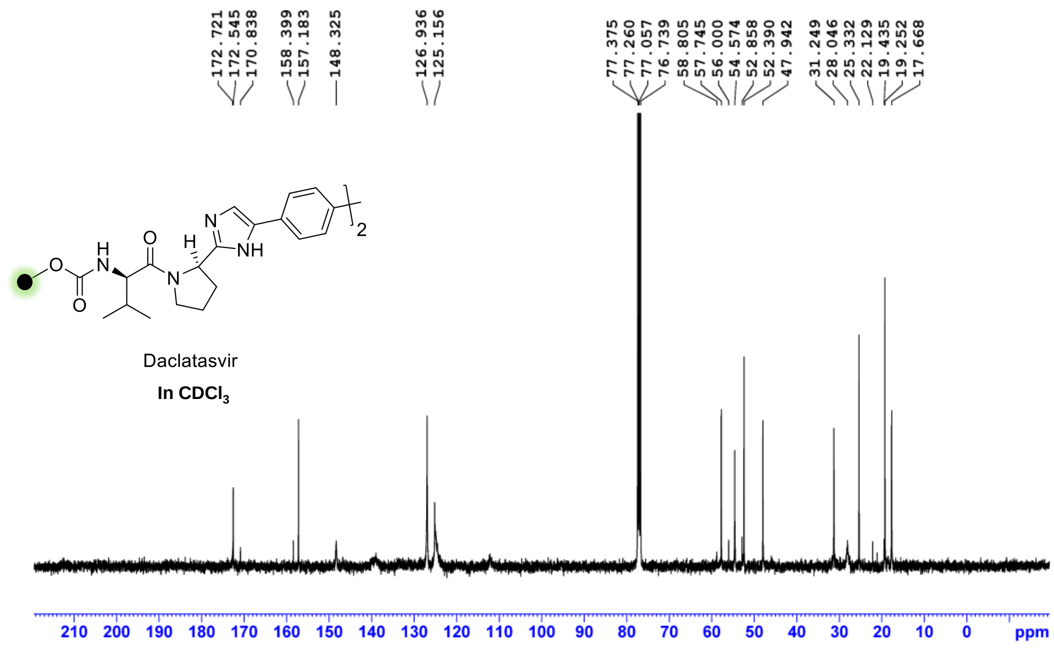
172.721
172.545
170.838
158.399
157.183
148.325
126.936
125.156
77.375
77.260
77.057
76.739
58.805
57.745
56.000
54.574
52.858
52.390
47.942
31.249
28.046
25.332
22.129
19.435
19.252
17.668
Daclatasvir
In CDCl3
210 200 190 180 170 160 150 140 130 120 110 100 90 80 70 60 50 40 30 20 10 0 ppm

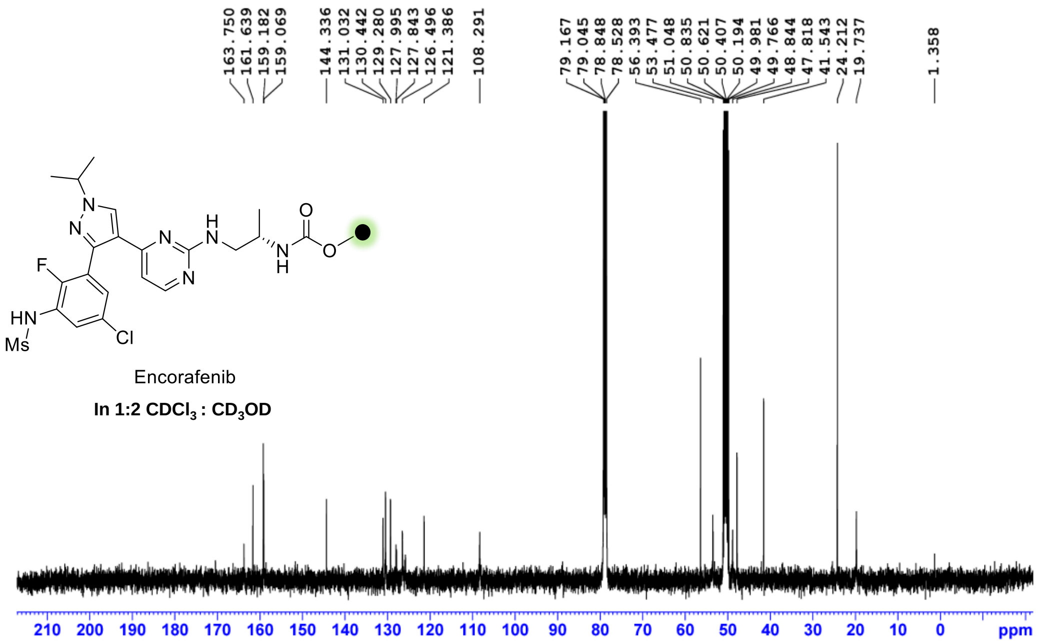
163.750
161.639
159.182
159.069
144.336
131.032
130.442
129.280
127.995
127.843
126.496
121.386
108.291
79.167
79.045
78.848
78.528
56.393
53.477
51.048
50.835
50.621
50.407
50.194
49.981
49.766
48.844
47.818
41.543
24.212
19.737
1.358
Encorafenib
In 1:2 CDCl3 : CD3OD
210 200 190 180 170 160 150 140 130 120 110 100 90 80 70 60 50 40 30 20 10 0 ppm

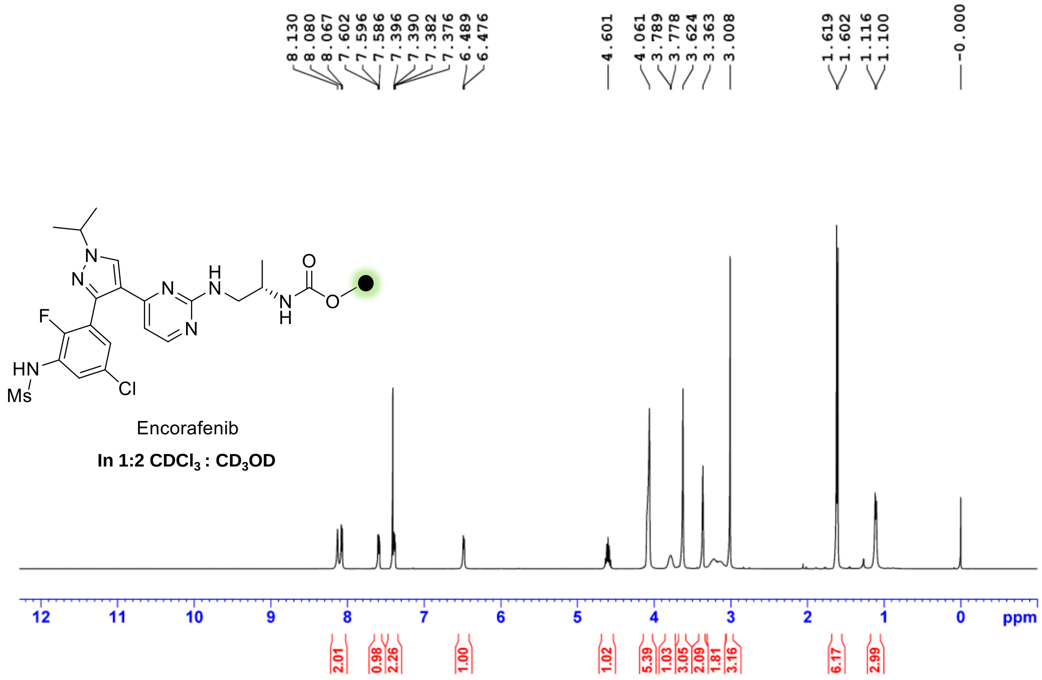
8.130
8.080
8.067
7.602
7.596
7.586
7.396
7.390
7.382
7.376
6.489
6.476
4.601
4.061
3.789
3.778
3.624
3.363
3.008
1.619
1.602
1.116
1.100
-0.000
Encorafenib
In 1:2 CDCl3 : CD3OD
12 11 10 9 8 7 6 5 4 3 2 1 0 ppm
2.01
0.98
2.26
1.00
1.02
5.39
1.03
3.05
2.09
1.81
3.16
6.17
2.99

References

[1] J. M. Nichols, L. M. Bishop, R. G. Bergman, J. A. Ellman, *J. Am. Chem. Soc.* **2010**, *132*, 12554-12555.

[2] C. S. Lancefield, O. S. Ojo, F. Tran, N. J. Westwood, *Angew. Chem. Int. Ed.* **2015**, *54*, 258-262.

[3] T. Ueda, H. Konishi, K. Manabe, *Org. Lett.* **2012**, *14*, 3100-3103.

[4] C. K. Lee, J. S. Yu, H.-J. Lee, *J. Heterocycl. Chem.* **2002**, *39*, 1207-1217.

[5] J. Ralph, Y. Zhang, R. M. Ede, *J. Chem. Soc. Perk. T. 1* **1998**, 2609-2614.

[6] D. Zhao, N. Wu, S. Zhang, P. Xi, X. Su, J. Lan, J. You, *Angew. Chem. Int. Ed.* **2009**, *48*, 8729-8732.

[7] a) L. Lu, S. Zou, B. Fang, *ACS Catal.* **2021**, *11*, 6020-6058; b) S. Vásquez-Céspedes, R. C. Betori, M. A. Cismesia, J. K. Kirsch, Q. Yang, *Org. Process Res. Dev.* **2021**, *25*, 740-753; c) C. Schröder, M. C. Schmidt, P. A. Haugg, A.-K. Baumann, J. Smyczek, S. Schauermann, *Angew. Chem. Int. Ed.* **2021**, *60*, 16349-16354; d) C. Schröder, A.-K. Baumann, M. C. Schmidt, J. Smyczek, P. A. Haugg, O.-C. Graap, S. Schauermann, *The Journal of Physical Chemistry C* **2022**, *126*, 4907-4920.

[8] Y. Izawa, I. Shimizu, A. Yamamoto, *Bull. Chem. Soc. Jpn.* **2004**, *77*, 2033-2045.

[9] Q. Liu, G. Li, J. He, J. Liu, P. Li, A. Lei, *Angew. Chem. Int. Ed.* **2010**, *49*, 3371-3374.

[10] D. M. Pearson, N. R. Conley, R. M. Waymouth, *Adv. Synth. Catal.* **2011**, *353*, 3007-3013.

[11] L. Ren, N. Jiao, *Chem. Asian J.* **2014**, *9*, 2411-2414.

[12] P. Tundo, M. Selva, *Acc. Chem.Res.* **2002**, *35*, 706-716.

[13] a) D. Gorbunov, M. Nenasheva, M. Terenina, Y. Kardasheva, A. Maksimov, E. Karakhanov, *ChemistrySelect* **2020**, *5*, 6407-6414; b) J. S. Lee, J. C. Kim, Y. G. Kim, *Appl. Catal.* **1990**, *57*, 1-30; c) M. S. Yalfani, G. Lolli, A. Wolf, L. Mleczko, T. E. Müller, W. Leitner, *Green Chem.* **2013**, *15*, 1146-1149.

[14] M. Liu, Z. Zhang, J. Yan, S. Liu, H. Liu, Z. Liu, W. Wang, Z. He, B. Han, *Chem* **2020**, *6*, 3288-3296.

[15] a) X. Li, X. Hong, *J. Organomet. Chem.* **2018**, *864*, 68-80; b) L. Hie, N. F. Fine Nathel, X. Hong, Y.-F. Yang, K. N. Houk, N. K. Garg, *Angew. Chem. Int. Ed.* **2016**, *55*, 2810-2814.

[16] a) H. Oshita, Y. Shimazaki, *Chem. Eur. J.* **2020**, *26*, 8324-8340; b) M. M. Whittaker, D. P. Ballou, J. W. Whittaker, *Biochemistry* **1998**, *37*, 8426-8436.

[17] F.-T. Du, J.-X. Ji, *Chem. Sci.* **2012**, *3*, 460-465.

[18] H. Inoue, Y. Liang, T. Yamada, N. Kimizuka, *Chem. Commun.* **2020**, *56*, 7013-7016.

[19] a) X. Zong, Q.-Z. Zheng, N. Jiao, *Org. Biomol. Chem.* **2014**, *12*, 1198-1202; b) A. Hassner, F. Boerwinkle, A. B. Lavy, *J. Am. Chem. Soc.* **1970**, *92*, 4879-4883; c) W. Wang, X. Li, X. Yang, L. Ai, Z. Gong, N. Jiao, S. Song, *Nat. Commun.* **2021**, *12*, 3873.

[20] Y.-F. Liang, N. Jiao, *Acc. Chem.Res.* **2017**, *50*, 1640-1653.

[21] A. S. K. Tsang, A. Kapat, F. Schoenebeck, *J. Am. Chem. Soc.* **2016**, *138*, 518-526.

[22] M. Liu, Z. Zhang, J. Song, S. Liu, H. Liu, B. Han, *Angew. Chem. Int. Ed.* **2019**, *58*, 17393-17398.

[23] U. S. Singh, R. T. Scannell, H. An, B. J. Carter, S. M. Hecht, *J. Am. Chem. Soc.* **1995**, *117*, 12691-12699.

[24] a) C. E. Jacobson, N. Martinez-Muñoz, D. J. Gorin, *J. Org. Chem.* **2015**, *80*, 7305-7310; b) S. Bénard, L. Neuville, J. Zhu, *Chem. Commun.* **2010**, *46*, 3393-3395; c) I. González, J. Mosquera, C. Guerrero, R. Rodríguez, J. Cruces, *Org. Lett.* **2009**, *11*, 1677-1680.

[25] a) H.-L. Fang, Q. Sun, R. Ye, J. Sun, Y. Han, C.-G. Yan, *New J. Chem.* **2019**, *43*, 13832-13836; b) B. L. Ryland, S. D. McCann, T. C. Brunold, S. S. Stahl, *J. Am. Chem. Soc.* **2014**, *136*, 12166-12173; c) S. D. McCann, S. S. Stahl, *Acc. Chem.Res.* **2015**, *48*, 1756-1766.
